# Supplementary material for: Implementing an Interactive Introduction to Complementary Medicine for Chronic Pain Management Into the Medical School Curriculum
Source: MedEdPORTAL. 2020 Dec 29;16:11056. doi: 10.15766/mep_2374-8265.11056 (PMC7780745; doi:10.15766/mep_2374-8265.11056)
Supplement: Supplementary file 1 — CAM Lecture.pptxStudent Perspective Script.docxFacilitator Guide.docxPresession Survey.docxPostSession Survey.docx [file mep_2374-8265.11056-s001.zip › _Educational_Summary_Report_11056.pdf]

MedEdPORTAL® | The AAMC Journal of Teaching and Learning Resources

Original Publication | Open Access

Implementing an Interactive Introduction to Complementary Medicine for Chronic Pain Management Into the Medical School Curriculum

Uttara Gadde*, Pravin Matthew, Raagni Kumar, Rashi Aggarwal, MD, Michelle Dalla Piazza, MD, Sangeeta Lamba, MD, MSHPE

*Corresponding author: ug26@njms.rutgers.edu

Abstract

Introduction: In the setting of the opioid crisis, chronic pain management requires new approaches and open dialogue between physicians and patients to explore evidence-based nonpharmacologic treatments. We developed an educational session on the role of complementary and alternative medicine (CAM) for chronic pain management as part of our larger curriculum on health equity and social justice. **Methods:** Students and faculty developed a novel educational session for second-year medical students consisting of a lecture and an experiential small-group session immersing the students in CAM. We conducted pre- and postsurveys to assess the students' self-reported learning and impressions of the session. **Results:** Over the academic years of 2018-2019 and 2019-2020, 345 second-year medical students participated in this mandatory session. In matched pre-and postsession surveys, students rated their knowledge of the evidence behind CAM practices, and reported statistically significant increases in their understanding. When asked about the importance of physician familiarity with common CAM practices, students noted both a high baseline agreement and a statistically significant increase after the session concluded. Familiarity with financial costs of each of the practices also saw statistically significant increases after the session. **Discussion:** Our results indicated that the session met the educational objectives. A critical part of improving our session between academic years involved gathering feedback and implementing changes based on these suggestions. Our model is easy to implement and replicate at medical schools across the country. Future studies should assess the effects of CAM-focused educational interventions on practices in the clinical setting.

Keywords
Pain, Pain Management, Pain Medicine, Opioid, Chronic Pain, Complementary Medicine, Alternative Medicine, Lecture, Integrative Medicine

Educational Objectives

By the end of this activity, learners will be able to:

1. Describe the evidence supporting the use of complementary treatment modalities in the management of chronic pain conditions.
2. Explain the importance of open communication between patients and physicians in discussing complementary therapies.
3. Examine how social inequity impacts patient access to pain management resources and complementary treatment.
4. Engage with a commonly employed complementary medicine modality for chronic pain management.

Citation:
Gadde U, Matthew P, Kumar R, Aggarwal R, Dalla Piazza M, Lamba S. Implementing an interactive introduction to complementary medicine for chronic pain management into the medical school curriculum. *MedEdPORTAL*. 2020;16:11056.
https://doi.org/10.15766/mep_2374-8265.11056

Introduction

Chronic pain represents a significant public health issue, affecting approximately one in five Americans. A debilitating disorder, chronic pain is also associated with depression and anxiety, poor perceived health or reduced quality of life, and restrictions in mobility and daily activities.[1] It is a condition that is not only devastating to the patient, but also has far-reaching consequences on the economy costing an estimated $560 billion annually in direct medical costs, disability programs, and lost productivity.[2] The management of chronic pain has become an especially important topic of discussion in recent years due to the ongoing opioid crisis. Initially intended for acute pain, opioids became a common treatment option of chronic pain by the 1990s. In 2016, 62 million patients had at least one opioid prescribed to them. Mirroring this rise in opioid prescriptions

Copyright © 2020 Gadde et al. This is an open-access publication distributed under the terms of the Creative Commons Attribution license.

was an unprecedented rise in deaths due to opioid overdose, a leading cause of death in Americans aged 15-24 years old.[3]

As data about the dangers of opioid use in the context of chronic pain management have become apparent, interest in nonpharmacologic approaches has increased. The National Center for Complementary and Alternative Medicine defines complementary and alternative medicine (CAM) as "a group of diverse medical and health care systems, practices, and products whose origins come from outside of mainstream medicine."[4] CAM can be utilized in conjunction with conventional medicine, leading to the emergence of integrative medicine as a specialty. In a survey of 831 patients who utilized both CAM and conventional medicine, 79% perceived the combination of both treatments to be superior to either one alone, indicating that Americans value a multifaceted approach to their care.[5] There are several metanalyses supporting the use of CAM and integrative medicine in the context of chronic pain.[6-9] Pain-related health conditions are the most commonly cited reason for using CAM, with 14 million Americans using CAM for back pain and 5 million using CAM for neck pain.[10] Approximately 33% of US adults use CAM, but despite its common use, 42% of CAM users do not discuss CAM with their primary care physicians. The 2012 National Center for Health Statistics annual survey illustrated that this disconnect could be due to physicians not asking about CAM use, or due to patient concerns about physician knowledge concerning CAM.[11] This is a striking indication that education about CAM modalities and patient-centered communication around CAM should be incorporated into medical school curricula, particularly in the context of pain, since CAM modalities are often recommended before initiating pharmacologic treatment. For example, the American College of Physicians clinical practice guidelines for chronic low back pain state that nonpharmacologic interventions (including massage and acupuncture) should be first-line treatments. Second line treatments include other nonpharmacologic treatments such as tai chi, yoga, and mindfulness-based stress reduction. Pharmacologic treatments are recommended only after nonpharmacologic treatments have been tried, yet these are rarely discussed in depth in the traditional medical school curriculum.[12]

Many have reported on the lack of formal exposure to CAM in medical school. The World Health Organization Congress on Traditional Medicine declared that one of its goals is to develop appropriate CAM training programs for biomedical and medical students.[13] Several studies have proposed models for incorporating integrative medicine into the current medical school curriculum.[14,15] In the context of pain medicine, a systematic review of 383 medical schools found that nonpharmacologic approaches to pain management were inconsistently taught and constituted a very limited part of the overall curriculum.[16] There are several major academic institutions currently changing this paradigm. For example, the Mayo Clinic created a 4-year medical school curriculum in integrative medicine, introducing the topic in the context of chronic pain through grand rounds and elective rotations in CAM methods such as acupuncture.[17] The University of Washington School of Medicine created a pain curriculum that included an observership in acupuncture and discussion about CAM pain treatments.[18]

When discussing CAM in the context of chronic pain, it is important to consider the associated health equity issues that arise. Studies suggest that chronic pain is more common in adults who have achieved lower levels of education and unemployed adults. Adults with these characteristics tend to have a more difficult time navigating the health care system, and often do not have the resources to explore CAM for pain relief.[1] Studies have shown that spending on CAM increases with higher family income, suggesting disparities in access to care based on socioeconomic status.[19] In 2012, Americans spent $30.2 billion on out-of-pocket costs for CAM treatments.[20] Only 32% of major insurance companies cover acupuncture, and other modalities (tai chi, yoga, etc.) are frequently not covered by insurance.[21] Future physicians should not only gain exposure to CAM as a component of chronic pain management through formalized education, but should also understand the social and financial barriers to accessing it.

In response, students and faculty at Rutgers New Jersey Medical School (NJMS) in Newark, New Jersey, collaborated to create a curriculum that addressed the evidence base for CAM in the context of chronic pain as well as inherent health equity issues as part of the Health Equity and Social Justice (HESJ) course. HESJ is a required longitudinal course spanning over the 2 preclerkship years of medical school that aims to make health equity content an explicit component of the medical school education. HESJ typically enrolls approximately 180 students per class per year. Sessions cover topics including unconscious bias, racial disparities in medicine, the social determinants of health, clinical empathy, religion/spirituality, and working with patients with disabilities, among others. We offered a 2.5-hour mandatory HESJ session to all second-year medical students in the academic year 2018-2019 (AY19) and 2019-2020 (AY20). The sessions consisted of a didactic lecture focused on CAM

Copyright © 2020 Gadde et al. This is an open-access publication distributed under the terms of the Creative Commons Attribution license.

and integrative medicine, followed by small-group sessions in which students signed up to experience a CAM modality of their choice, which included yoga, meditation, and tai chi (tai chi was only offered during AY20). Faculty or other local experts in their respective CAM field ran each group session.

Our approach and format was novel and unique. One *MedEdPORTAL* publication discusses using a team-based learning model to introduce students to pharmacologic pain management strategies.[22] and there is another publication discussing the use of standardized patients to teach about chronic pain patients at risk for drug abuse.[23] However, no other publication focuses on nonpharmacologic approaches to treating chronic pain, or addresses the topic using an experiential component. In addition, no other publication addresses the social justice issues associated with nonpharmacologic approaches to chronic pain management. As in other examples of health equity training, having students actively engaged in the organization of the lecture and curriculum design proved successful.[24]

Methods

Background Resources and Preparation

We decided to introduce students to this topic during their second year of medical school, after students had completed the musculoskeletal organ system course and prior to the start of the neurology course. With an introduction to anatomy and an understanding of the pathophysiology of musculoskeletal pain, we hoped that this introduction to CAM would allow students to put the pain pathways they would ultimately learn about during their neurology block into context. In addition, it was important to include the discussion about social justice issues related to access to CAM during the preclerkship years, so that students could keep this perspective in mind when interacting with patients during the clerkships in the third year.

To prepare for the session, we recommended that the students read a narrative piece by Kevin F. Boehnke.[25] The piece reflects on the writer's experience with chronic pain and CAM modalities, as well as his inspiration for a career choice to pursue chronic pain research. One week in advance of the session, we also sent out an online survey to ask students which immersive CAM experience they preferred. We then assigned the students to a small-group session based on availability and their preference, making every effort to accommodate the students' indicated interests.

This session also falls into the larger context of the HESJ course. Prior to this session we had introduced the ETHNIC framework to the students in the first year of the course;[26] a subsequent session in the second year revisits this framework in the context of motivational interviewing. As a guide to patient-centered interviewing, the ETHNIC framework integrates questions pertaining to the patient's explanation and expectations: E for their health concerns; T for treatments they've tried including home remedies and CAM; and H is for healers they have consulted. The "NIC" portion then asks students and providers to: N, negotiate a mutually acceptable treatment plan, design, and intervention; I, incorporate the patient's values and CAM as appropriate; and C, collaborate to determine who will be needed to enact the plan, including, family members, spiritual healers, and CAM practitioners.[26]

Didactic Lecture

The didactic lecture (Appendix A) required a large lecture hall and projection screen to accommodate the entire second-year class. We used PowerPoint slides and allotted 1.5 hours for this didactic portion. In the first part of the didactic session, we presented an overview of the current evidence base behind several commonly used CAM modalities, including acupuncture, yoga, meditation, tai chi, reiki, and spinal manipulation. A faculty member familiar with the literature surrounding these topics reviewed the existing literature (including major meta-analyses) to provide an overview of the evidence base as well as any contraindications for each modality. This presentation ensured that students were well informed about the body of scientific literature available.

Immediately after, we presented a patient case. In AY19, the case consisted of a personal story of a medical student who had experienced chronic pain and successfully used CAM to manage her pain. Based on student feedback requesting exploring patient stories in more depth, in AY20 we presented her story as a multiple-part guided discussion of an anonymous patient suffering from chronic pain. After presenting the history of present illness, the class was asked how they would advise this patient, who was also interested in exploring CAM modalities. This allowed students to consider how they would engage in an empathetic as well as evidence-based discussion with future patients. Students contributed to this discussion in a large-group setting by volunteering their answers to discussion questions. After the guided discussion, the student patient volunteered to share her story at the conclusion of the case (Appendix B). This allowed students to fully appreciate that chronic pain can affect anyone, and that learning about these modalities can be truly impactful, even within their own networks.

The final component of the session served as a reflection on the social inequities inherent in the lack of insurance coverage for and access to CAM modalities, including a discussion of the

Copyright © 2020 Gadde et al. This is an open-access publication distributed under the terms of the Creative Commons Attribution license.

financial burden of out-of-pocket expenses for CAM treatments. We asked students to reflect on how these inequities in access to care might affect the patient experience and outcomes, both in the context of our case patient, and in the context of the local patient population.

Small-Group Interactive Sessions

After completing the didactic portion of the session, students dispersed to their assigned small-group session. In AY19, CAM small-group sessions included yoga and meditation. In AY20, CAM small-group sessions expanded to include yoga, meditation, and tai chi. All small-group sessions were led by faculty or other community members with expertise in a specific CAM modality. Each small group consisted of approximately 15-30 students.

The small-group sessions lasted approximately 40-50 minutes. Small-group session facilitators led students in an active introduction to their assigned CAM method. Students in the yoga groups performed introductory yoga, with explanations about the effects of different postures on the mind and body. Students in the meditation sessions learned about different types of meditation and actively engaged in a mindfulness practice. Students in the tai chi session performed a tai chi sequence under supervision, with explanations corresponding to each of the movements. Facilitators followed the facilitator guide (Appendix C) for their respective CAM modality.

Evaluation

We asked all second-year students to complete both pre- and postsurveys (Appendices D and E respectively) in Qualtrics. The survey questions were developed by the study authors to measure if the sessions were able to meet the objectives outlined and as a way to elicit feedback. The surveys asked students to describe their interest, knowledge of evidence base, and familiarity with CAM techniques as measured on a 5-point Likert scale, both before and then within a week after completion of the didactic and small-group interactive sessions. Students were also asked to comment on the strengths of the session as well as suggestions for improvement in narrative form.

Data Analysis

After being granted institutional review board approval for analysis of the data collected, two of the authors (Uttara Gadde and Pravin Matthew) then independently analyzed the narrative data, with responses assigned to themes for comparison. Numeric data were exported from Qualtrics into Microsoft Excel for analysis, which included descriptive statistics and unpaired two sample *t* tests to check for statistical significance of any differences between the pre- and postresponses.

Results

Over both academic years, a total of 345 second-year medical students participated in the mandatory session. All students attended the mandatory didactic session. In AY19, 68% (99 out of 146) of students participated in a meditation/mindfulness session and 32% (47 of 146) of students participated in a yoga session. In AY20, 48% (56 of 117) of students participated in meditation/mindfulness session, 28% (33 of 117) of students participated in a yoga session, and 24% (28 of 117) participated in a tai chi session.

Presurvey Data

In AY19, 98% (175 of 178) of students, and in AY20 96% (170 of 178) of students completed the presurvey prior to their session. Interest level in learning more about CAM practices for managing chronic pain proved similar across both years, with mean interest measured as 3.6 and 3.6 for AY19 and AY20 respectively, on a 5-point Likert scale. Familiarity with common practices such as yoga, tai chi, meditation/mindfulness, and acupuncture also displayed similar trends, with students reporting more familiarity with yoga and meditation/mindfulness and less so with tai chi and acupuncture.

Of students, 59% (104 of 175) surveyed in AY19 and 44% (74 of 170) surveyed in AY20 reported witnessing at least one episode of bias against patients who practiced a CAM discipline by a member of the health care team in the past year. Reports of personal bias against patients who practiced a CAM discipline were consistent across years, with 66% (116 of 175) of students in AY19 and 68% (116 of 170) of students in AY20 reporting at least a small degree of bias.

Analysis of Postsurvey Data in Context

Of students, 146 (86%) in AY19 and 117 students (67%) in AY20 completed the postsurvey within 1 week of the completion of their sessions.

We asked students to rate their knowledge of the evidence behind CAM practices both before and after completion of the session. In matched pre-and postsurveys for both years, students reported statistically significant ($p < .01$) increases in their understanding of the evidence for all practices covered (Figure 1). We also asked students to assess their comfort in counseling patients about CAM practices for chronic pain and reported similarly significant ($p < .01$) increases in their comfort levels after the session concluded.

When asked about the importance of physician familiarity with common CAM practices, students noted both a high

Copyright © 2020 Gadde et al. This is an open-access publication distributed under the terms of the Creative Commons Attribution license.

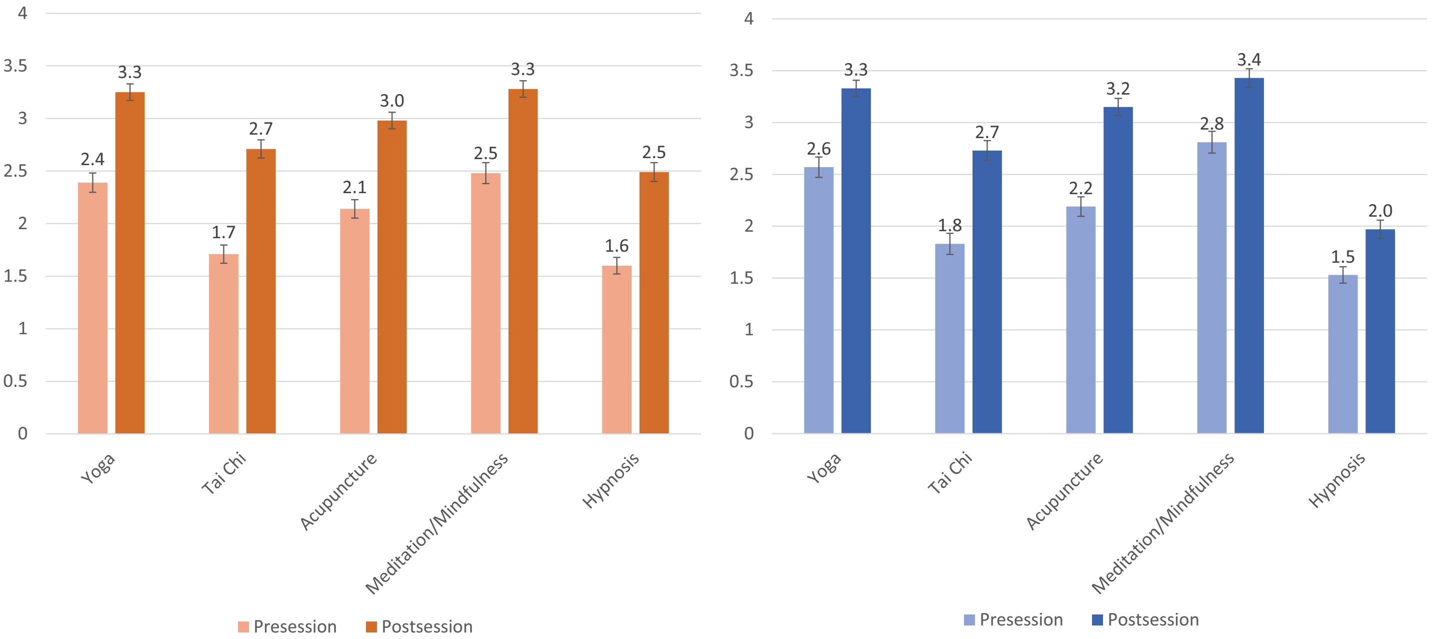

Figure 1. Left panel: 2018-2019 (AY19) second-year student responses to the survey question: **"**Rate your knowledge about the evidence base behind common complementary medicine practices for treating chronic pain,**"** (1 = *minimal knowledge*, 5 = *superior knowledge*). Values shown represent the mean; error bars represent standard error; all differences were significant ($p < .01$). Right panel: 2019-2020 (AY20) second-year student responses to the survey question: "Rate your knowledge about the evidence base behind common complementary medicine practices for treating chronic pain," (1 = *minimal knowledge*, 5 = *superior knowledge*). Values shown represent the mean; error bars represent standard error; all differences were significant ($p < .01$).

baseline agreement (means of 3.6 and 3.7, in AY19 and AY20, respectively) and a statistically significant ($p < .01$) increase after the session concluded (means increased to 4.0 and 4.0, in AY19 and AY20, respectively). Familiarity with financial costs of each of the practices also saw statistically significant ($p < .01$) increases after the session (Figure 2).

Students noted improvement in their ability to meet each learning objective after completion of the session, with the highest levels reported for explaining the importance of open communication between patients and physicians in discussing CAM therapies (Figure 3).

Noted strengths of the session were similar across years, but the AY20 students (Table) specifically noted strengths including the comprehensive nature of the lecture portion, the reliance on evidence, the student presenter's personal experience, and small-group sessions as both informative and engaging. Suggestions for improvement from the AY20 students centered on allowing students to try each of the modalities, the need for more information on the financial costs of each practice, providing more information on fewer modalities, and the chance to hear more personal accounts from patients who have employed these practices.

Discussion

To address the growing interest by the public in using CAM modalities to treat chronic pain, we developed a didactic and interactive session that reviewed the evidence base behind commonly used CAM modalities, addressed the social inequities in terms of access to CAM care, and allowed students to immerse themselves in a CAM modality of their choice. Our effort proved successful in providing students with a basic understanding of the evidence base available for CAM techniques for the management of chronic pain, the importance of patient-centered communication around CAM, and the disparities in access to the modalities to consider when counseling a patient about these methods. In addition, the hands-on small group sessions allowed students to experience CAM methods firsthand, so that they can be better versed in engaging in discussions about these modalities with their patients. This multipronged approach ensured that students received a comprehensive overview of the science, communication, social implications, and actual practice of common CAM modalities in an engaging format.

By integrating a discussion about the evidence base behind widely used CAM modalities into our medical school curriculum, we hoped to encourage future physicians to actively engage in conversations about the utility of CAM with their patients. A

Copyright © 2020 Gadde et al. This is an open-access publication distributed under the terms of the Creative Commons Attribution license.

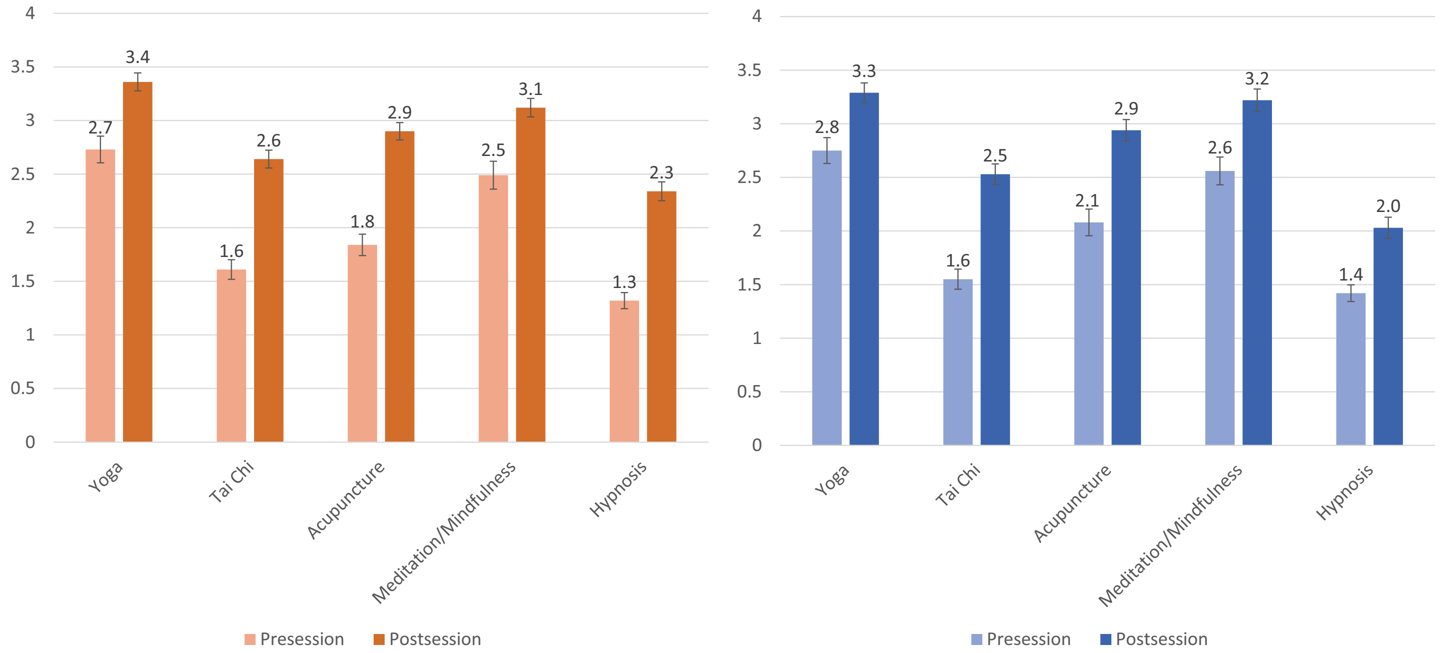

Figure 2. Left panel: 2018-2019 (AY19) second-year student responses to the survey question: "How familiar are you with the financial costs associated with these practices?" (1 = *not familiar at all*, 5 = *very familiar*). Values shown represent the mean; error bars represent standard error; all differences were significant ($p < .01$). Right panel: 2019-2020 (AY20) second-year student responses to the survey question: "How familiar are you with the financial costs associated with these practices?" (1 = *not familiar at all*, 5 = *very familiar*). Values shown represent the mean; error bars represent standard error; all differences were significant ($p < .01$).

greater understanding about the strengths and limitations of various modalities will allow future physicians to make informed recommendations and facilitate greater transparency and trust in the patient-physician relationship.

Given the first set of feedback from AY19, we implemented a number of changes for AY20, including shortening the lecture portion to have a more focused discussion on the most common CAM methods and introducing a case discussion to provide

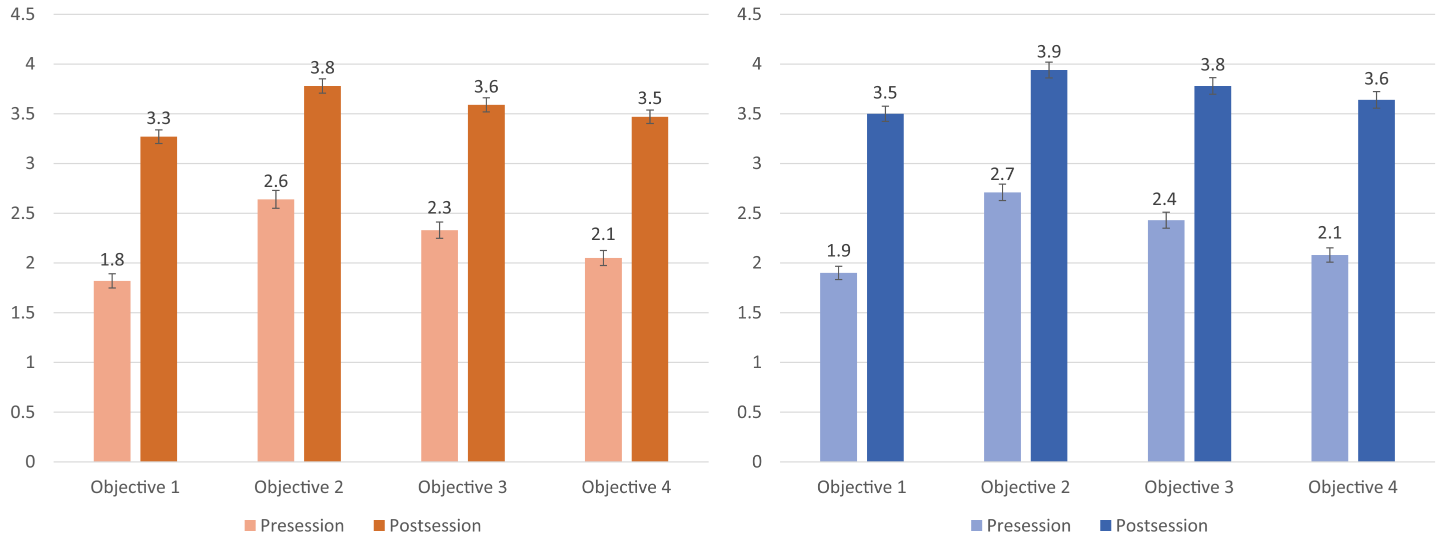

Figure 3. Left panel: 2018-2019 (AY19) second-year student responses to the survey question: "Rate your ability to accomplish the following after completing this educational session," (1 = *hardly at all*, 5 = *to a very high degree*). Values shown represent the mean; errors bars represent standard error; all differences were significant ($p < .01$). Right panel: 2019-2020 (AY20) second-year student responses to the survey question: "Rate your ability to accomplish the following after completing this educational session," (1 = *hardly at all*, 5 = *to a very high degree*). Values shown represent the mean; errors bars represent standard error; all differences were significant ($p < .01$).

Copyright © 2020 Gadde et al. This is an open-access publication distributed under the terms of the Creative Commons Attribution license.

Table. Commonly Cited Strengths (*N* = 114) and Suggestions for Improvement (*N* = 101) After Completing the AY20 Session

Common Themes	*n*[a]	Example
Strengths		
Small group sessions were a major highlight.	59	"It was a good idea to have each student participate in a method of CAM. Now we can all at least share our experience with a method of CAM."
Comprehensive introduction to the evidence base behind CAM techniques.	47	"The session did a great job of going through the most popular complementary medicine practices and listing the benefits of those practices for the treatment of chronic pain. It was a very comprehensive lecture."
Lecture was engaging and well organized.	22	"The lecturers were super approachable and made the material interesting."
Student perspective/case discussion was important for putting information in context.	14	"The PowerPoint was very informative, but the best part of the session was having a real person come in and talk about her experiences with this and how it helped her."
Suggestions for Improvement		
Ability to try more modalities/spend more time practicing relative to lecture.	35	"I think a variety of integration would be nice, 10 minutes for meditation, 10 for tai chi and another 20 for yoga, so everyone gets to experience variety"
Cover fewer modalities/go into greater depth during the lecture.	17	"I think the session should zero in on a common few (yoga, meditation, etc.) that are widely used instead of trying to cover such a broad range of practices that a lot of students had not even heard of. This would at least provide us with a strong base, even if our knowledge isn't that broad."
More patients'/physicians' perspectives.	17	"It might be interesting to have practitioners of each modality come speak about their practice and how it's helped their patients"
More information on financial costs.	7	"Include more financial explanations as to what complementary medicine practices would actually be available under certain insurances. And talk about the financial barriers that patients might encounter when seeking such treatments."

Abbreviations: CAM, complementary and alternative medicine.
[a]The number of responses fitting a theme; some students listed multiple strengths or improvements in their responses.

students an opportunity to practice counseling patients in a real-world context. In addition, we held a tai chi session in AY20, which further broadened the experiential session. Students appreciated these changes, based on the narrative data we received from AY20.

Many students asked for more time to experience each of the different modalities we offered and even additional modalities that we were not able to offer due to lack of an instructor, such as acupuncture. Given the feedback, we reflected on whether it was better to have students experience one comprehensive experience in each modality versus shorter rotations so that each student could try multiple modalities. After discussing with our CAM instructors, given logistical constraints, we collectively agreed that having students go through a proper introduction to one modality would be more appropriate and feasible. We hope to introduce more modalities and organize students into smaller groups as instructors become available.

Another common suggestion asked for a wider presentation of CAM patients' perspectives. In both iterations of our course, we had a student present their personal story of how CAM modalities have been instrumental in their treatment program. Students consistently praised this presentation as a key reason they felt that CAM modalities should be further explored in medical education. Some students therefore asked to hear from patients/clients of our instructors to get a broader sense of how patients felt that these treatments helped them. While this does introduce another component to the session, this is something we plan to implement in future iterations.

It is important to note that our survey data relied on self-reported answers. We did not conduct any formalized assessment to objectively demonstrate how much information the students retained over time or how this education will impact their future practice. However, the results indicated that the session achieved its obejctives. This was particularly important because the format of our session is very easy to implement across all medical schools. It only requires one time slot, accessibility to a large lecture hall, and a few smaller classrooms. One potential challenge was that small-group facilitators for different CAM modalities must be identified. It may prove difficult to find a local qualified CAM practitioner that feels comfortable teaching in an academic setting. One way to address this challenge and offer a more standardized approach would be to provide video recordings of small-group sessions and distribute these recordings to interested medical schools to use as a guide or in lieu of a live facilitator. Another potential limiting factor in reviewing our data was the fact that we included a brief paragraph introducing students to the context for our session in our presurvey (Appendix D). This may have slightly skewed student responses prior to the session.

The opioid crisis coupled with an increasing interest among Americans in exploring CAM modalities to treat chronic pain are compelling reasons to integrate a discussion about CAM into

Copyright © 2020 Gadde et al. This is an open-access publication distributed under the terms of the Creative Commons Attribution license.

the medical school curriculum. In addition, it is important that students are aware of the financial burden of these practices and inequities in access to care. Our session addressed these needs and was effective and easy to replicate in medical schools nationally. The self-reported survey results showed significant improvements in baseline knowledge regarding CAM. Further studies are needed to assess the effects of this intervention on outcomes in practice. As patients increasingly explore CAM as part of therapeutic management for chronic pain, physicians must rise to the growing demand and be equipped with the tools to counsel their patients effectively and compassionately.

Appendices

A. CAM Lecture.pptx

B. Student Perspective Script.docx

C. Facilitator Guide. docx

D. Presession Survey.docx

E. PostSession Survey.docx

All appendices are peer reviewed as integral parts of the Original Publication.

Uttara Gadde: Medical Student, Rutgers New Jersey Medical School; ORCID: https://orcid.org/0000-0002-2615-5794

Pravin Matthew: Medical Student, Rutgers New Jersey Medical School; ORCID: https://orcid.org/0000-0001-5067-4458

Raagni Kumar: Medical Student, Rutgers New Jersey Medical School

Rashi Aggarwal, MD: Associate Professor and Program Director of Residency Training, Department of Psychiatry, Rutgers New Jersey Medical School

Michelle Dalla Piazza, MD: Assistant Professor, Department of Medicine, Division of Infectious Diseases, Rutgers New Jersey Medical School

Sangeeta Lamba, MD, MSHPE: Vice Chancellor for Diversity and Inclusion, Rutgers Biomedical and Health Sciences

Disclosures
None to report.

Funding/Support
None to report.

Ethical Approval
The Newark Health Sciences Institutional Review Board approved this study.

References

1. Dahlhamer J, Lucas J, Zelaya C, et al. Prevalence of chronic pain and high-impact chronic pain among adults—United States, 2016. *MMWR Morb Mortal Wkly Rep*. 2018;67(36):1001-1006. https://doi.org/10.15585/mmwr.mm6736a2
2. Institute of Medicine (US) Committee on Advancing Pain Research, Care, and Education. *Relieving Pain in America: A Blueprint for Transforming Prevention, Care, Education, and Research*. National Academies Press; 2011.
3. Rummans TA, Burton MC, Dawson NL. How good intentions contributed to bad outcomes: the opioid crisis. *Mayo Clin Proc*. 2018;93(3):344-350. https://doi.org/10.1016/j.mayocp.2017.12.020
4. Are you considering a complementary health approach? National Center for Complementary and Integrative Health. Updated September 2016. Accessed May 19, 2020. https://www.nccih.nih.gov/health/are-you-considering-a-complementary-health-approach
5. Eisenberg DM, Kessler RC, Van Rompay MI, et al. Perceptions about complementary therapies relative to conventional therapies among adults who use both: results from a national survey. *Ann Intern Med*. 2001;135(5):344-351. https://doi.org/10.7326/0003-4819-135-5-200109040-00011
6. Nahin RL, Boineau R, Khalsa PS, Stussman BJ, Weber WJ. Evidence-based evaluation of complementary health approaches for pain management in the United States. *Mayo Clin Proc*. 2016;91(9):1292-1306. https://doi.org/10.1016/j.mayocp.2016.06.007
7. Veehof MM, Trompetter HR, Bohlmeijer ET, Schreurs KMG. Acceptance- and mindfulness-based interventions for the treatment of chronic pain: a meta-analytic review. *Cogn Behav Ther*. 2016;45(1):5-31. https://doi.org/10.1080/16506073.2015.1098724
8. Holtzman S, Beggs RT. Yoga for chronic low back pain: a meta-analysis of randomized controlled trials. *Pain Res Manage*. 2013;18(5):267-272. https://doi.org/10.1155/2013/105919
9. Wilkinson J, Faleiro R. Acupuncture in pain management. *Continuing Educ Anaesth Crit Care Pain*. 2007;7(4):135-138. https://doi.org/10.1093/bjaceaccp/mkm021
10. Barnes PM, Bloom B, Nahin RL. Complementary and alternative medicine use among adults and children: United States, 2007. National Health Statistics Report. Published December 10, 2008. Accessed September 25, 2020. https://www.cdc.gov/nchs/data/nhsr/nhsr012.pdf
11. Jou J, Johnson PJ. Nondisclosure of complementary and alternative medicine use to primary care physicians. *JAMA Intern Med*. 2016;176(4):545-546. https://doi.org/10.1001/jamainternmed.2015.8593
12. Qaseem A, Wilt TJ, McLean RM, Forciea MA; Clinical Guidelines Committee of the American College of Physicians. Noninvasive

Copyright © 2020 Gadde et al. This is an open-access publication distributed under the terms of the Creative Commons Attribution license.

treatments for acute, subacute, and chronic low back pain: a clinical practice guideline from the American College of Physicians. *Ann Intern Med*. 2017;166(7):514-530. https://doi.org/10.7326/M16-2367

13. Quartey NK, Ma PHX, Chung VCH, Griffiths SM. Complementary and alternative medicine education for medical profession: systematic review. *Evid Based Complement Alternat Med*. 2012;2012:656812. https://doi.org/10.1155/2012/656812
14. Shipton EE, Bate F, Garrick R, Steketee C, Visser EJ. Pain medicine content, teaching and assessment in medical school curricula in Australia and New Zealand. *BMC Med Educ*. 2018;18:110. https://doi.org/10.1186/s12909-018-1204-4
15. Loeser JD, Schatman ME. Chronic pain management in medical education: a disastrous omission. *PostgradMed*. 2017;129(3): 332-335. https://doi.org/10.1080/00325481.2017.1297668
16. Shipton EE, Bate F, Garrick R, Steketee C, Shipton EA, Visser EJ. Systematic review of pain medicine content, teaching, and assessment in medical school curricula internationally. *Pain Ther*. 2018;7(2):139-161. https://doi.org/10.1007/s40122-018-0103-z
17. Cutshall SM, Khalsa TK, Chon TY, et al. Curricular development and implementation of a longitudinal integrative medicine education experience for trainees and health-care professionals at an academic medical center. *Global Adv Health Med*. 2019;8:1-7. https://doi.org/10.1177/2164956119837489
18. Tauben DJ, Loeser JD. Pain education at the University of Washington School of Medicine. *J Pain*. 2013;14(5):431-437. https://doi.org/10.1016/j.jpain.2013.01.005
19. Nahin RL, Barnes PM, Stussman BJ. Expenditures on complementary health approaches: United States, 2012. CDC National Health Statistics Report no. 95. Published June 22, 2016. Accessed September 25, 2020. https://www.cdc.gov/nchs/data/nhsr/nhsr095.pdf
20. Paying for complementary and integrative health approaches. National Center for Complementary and Integrative Health. Updated June 2016. Accessed May 19, 2020. https://www.nccih.nih.gov/health/paying-for-complementary-and-integrative-health-approaches
21. Prindle L. Your guide to alternative medicine and health insurance. QuoteWizard. Published June 6, 2018. Accessed May 19, 2020. https://quotewizard.com/health-insurance/complementary-and-alternative-medicine-and-health-insurance
22. Zgheib N, Kanazi G, Saasouh W. Management of nociceptive pain for third-year medical students-team based learning (TBL) format. *MedEdPORTAL*. 2012;8:9221. https://doi.org/10.15766/mep_2374-8265.9221
23. Yelen M, Anderson L, Wright K, Fleming M. Using SPs to teach about patients at risk for drug abuse. *MedEdPORTAL*. 2014;10:9907. https://doi.org/10.15766/mep_2374-8265.9907
24. DallaPiazza M, Padilla-Register M, Dwarakanath M, Obamedo E, Hill J, Soto-Greene ML. Exploring racism and health: an intensive interactive session for medical students. *MedEdPORTAL*. 2018; 14:10783. https://doi.org/10.15766/mep_2374-8265.10783
25. Boehnke KF. Pain management: assembling a tool kit, building a life. *JAMA*. 2018;320(21):2201-2202. https://doi.org/10.1001/jama.2018.17722
26. Levin SJ, Like RC, Gottlieb JE. ETHNIC: a framework for culturally competent clinical practice. In: Appendix: useful clinical interviewing mnemonics. *Patient Care*. 2000;34(9):188-189.

Received: May 29, 2020
Accepted: August 31, 2020
Published: December 29, 2020

Copyright © 2020 Gadde et al. This is an open-access publication distributed under the terms of the Creative Commons Attribution license.
