# Supplementary material for: Implementing an Interactive Introduction to Complementary Medicine for Chronic Pain Management Into the Medical School Curriculum
Source: MedEdPORTAL. 2020 Dec 29;16:11056. doi: 10.15766/mep_2374-8265.11056 (PMC7780745; doi:10.15766/mep_2374-8265.11056)
Supplement: Supplementary file 1 — CAM Lecture.pptxStudent Perspective Script.docxFacilitator Guide.docxPresession Survey.docxPostSession Survey.docx [file mep_2374-8265.11056-s001.zip › A. CAM Lecture.pptx]

## Slide 1
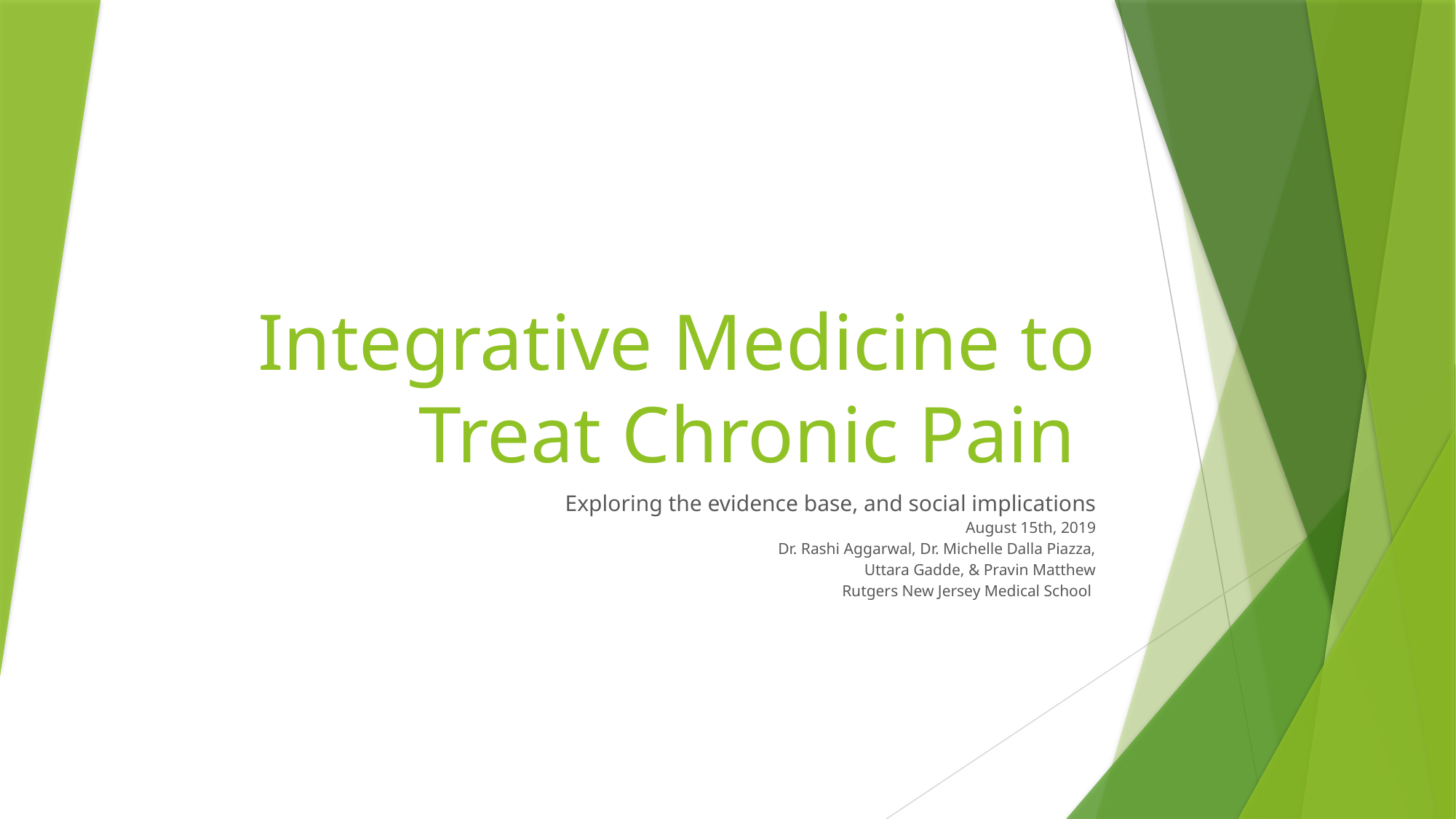

# Integrative Medicine to Treat Chronic Pain
Exploring the evidence base, and social implications
August 15th, 2019
Dr. Rashi Aggarwal, Dr. Michelle Dalla Piazza,
Uttara Gadde, & Pravin Matthew
Rutgers New Jersey Medical School

## Slide 2
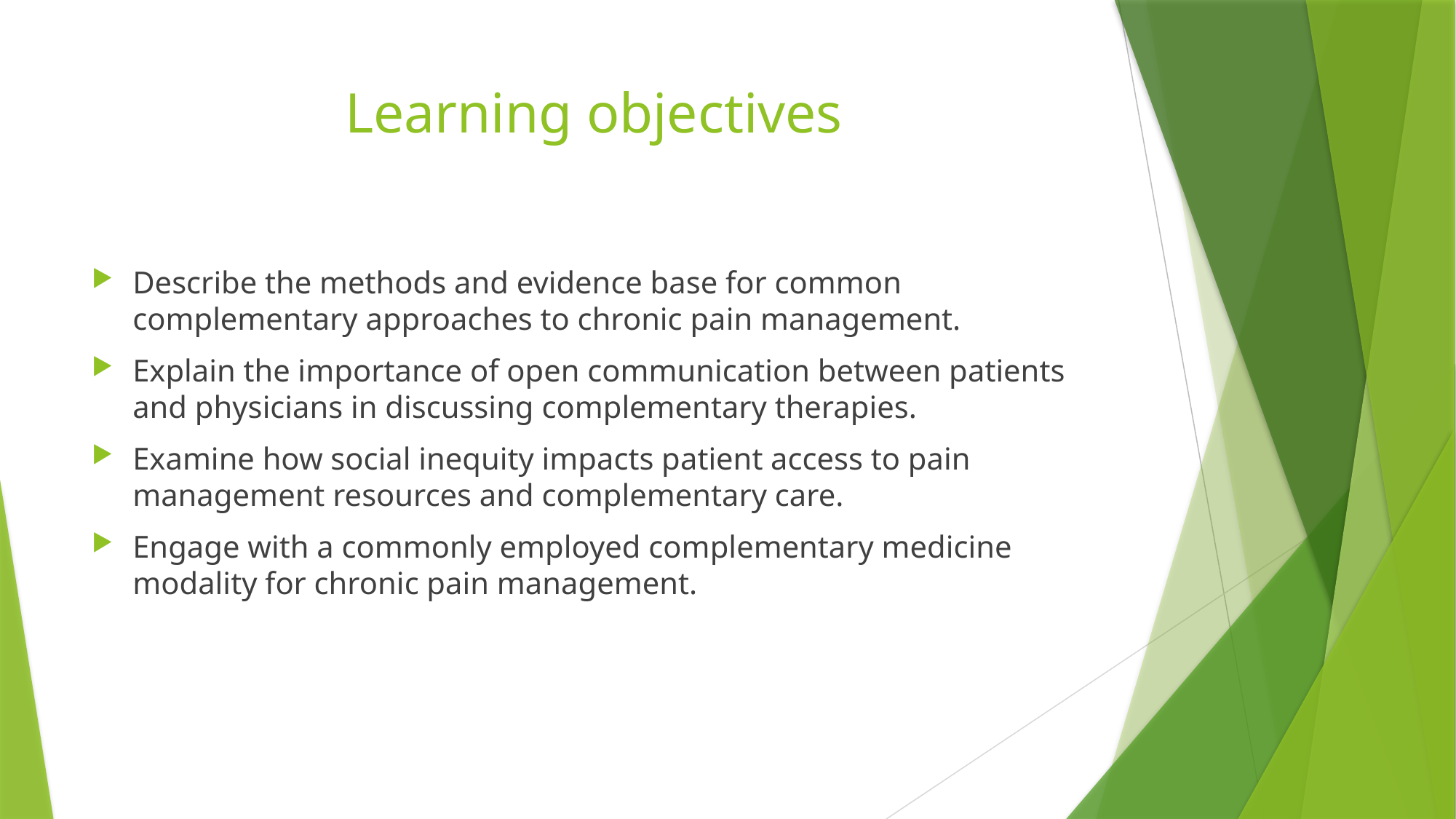

# Learning objectives
Describe the methods and evidence base for common complementary approaches to chronic pain management.
Explain the importance of open communication between patients and physicians in discussing complementary therapies.
Examine how social inequity impacts patient access to pain management resources and complementary care.
Engage with a commonly employed complementary medicine modality for chronic pain management.

## Slide 3
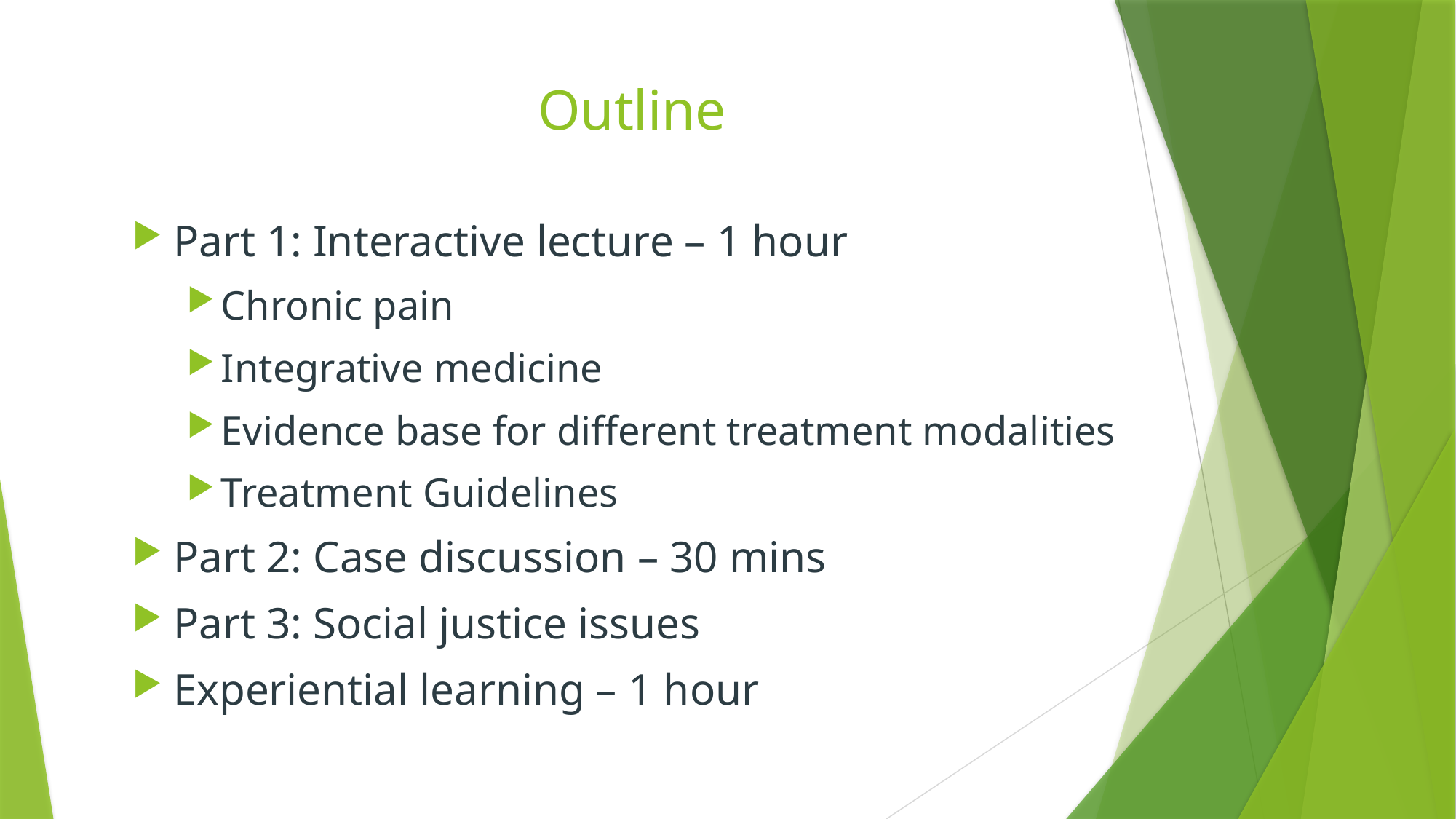

# Outline
Part 1: Interactive lecture – 1 hour
Chronic pain
Integrative medicine
Evidence base for different treatment modalities
Treatment Guidelines
Part 2: Case discussion – 30 mins
Part 3: Social justice issues
Experiential learning – 1 hour

## Slide 4
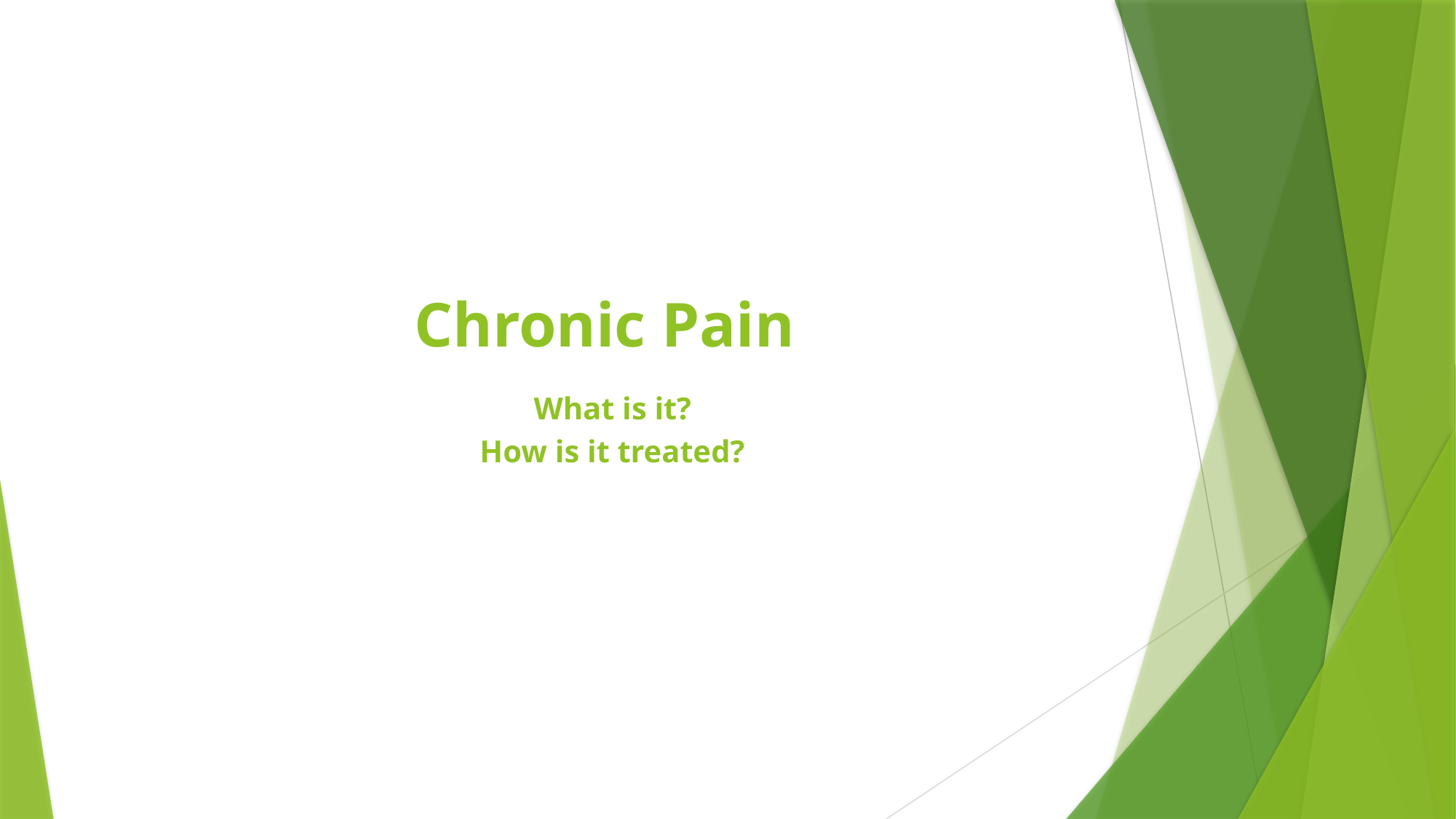

# Chronic Pain What is it? How is it treated?

## Slide 5
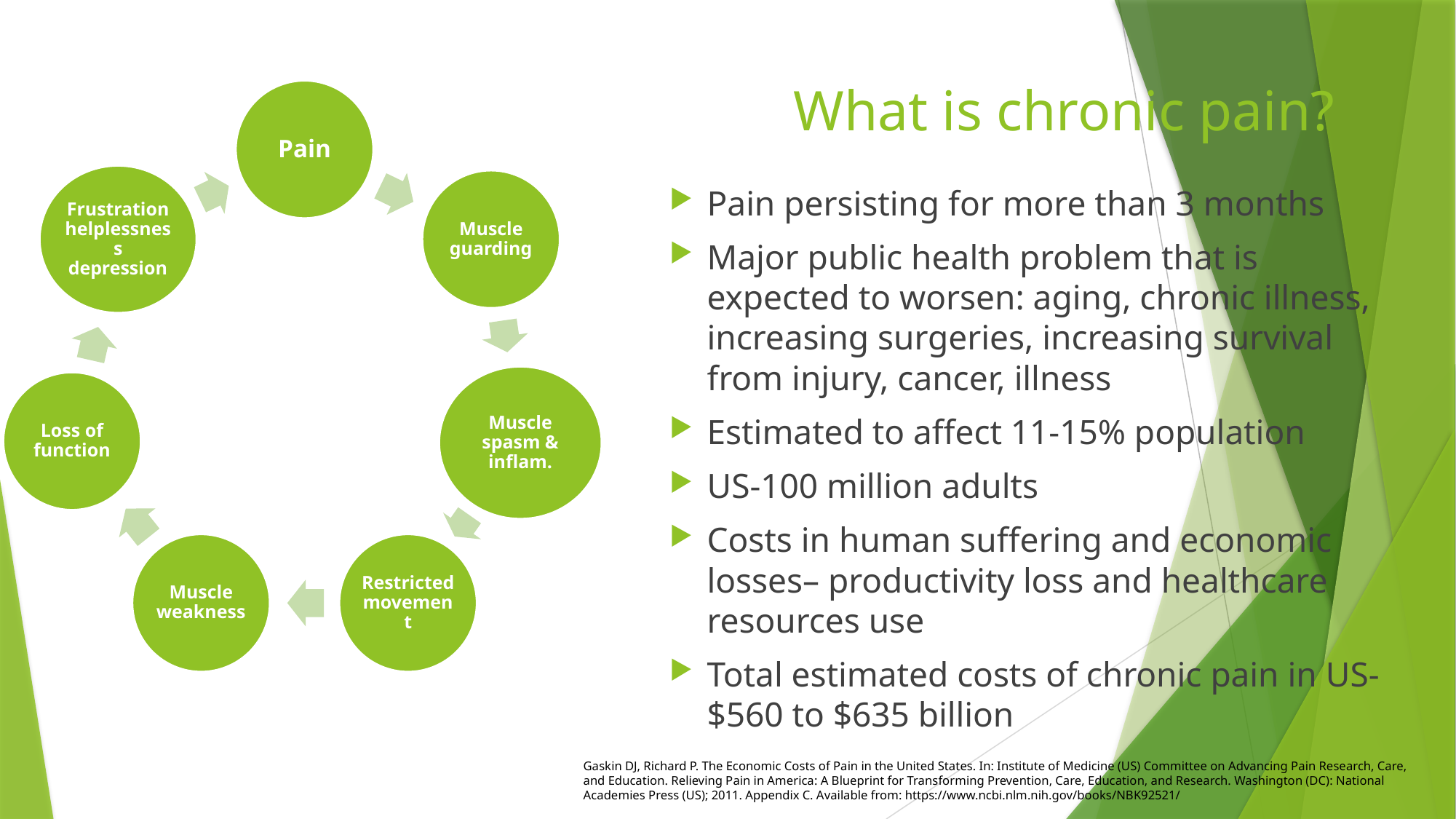

# What is chronic pain?
Pain persisting for more than 3 months
Major public health problem that is expected to worsen: aging, chronic illness, increasing surgeries, increasing survival from injury, cancer, illness
Estimated to affect 11-15% population
US-100 million adults
Costs in human suffering and economic losses– productivity loss and healthcare resources use
Total estimated costs of chronic pain in US- $560 to $635 billion
Gaskin DJ, Richard P. The Economic Costs of Pain in the United States. In: Institute of Medicine (US) Committee on Advancing Pain Research, Care, and Education. Relieving Pain in America: A Blueprint for Transforming Prevention, Care, Education, and Research. Washington (DC): National Academies Press (US); 2011. Appendix C. Available from: https://www.ncbi.nlm.nih.gov/books/NBK92521/

## Slide 6
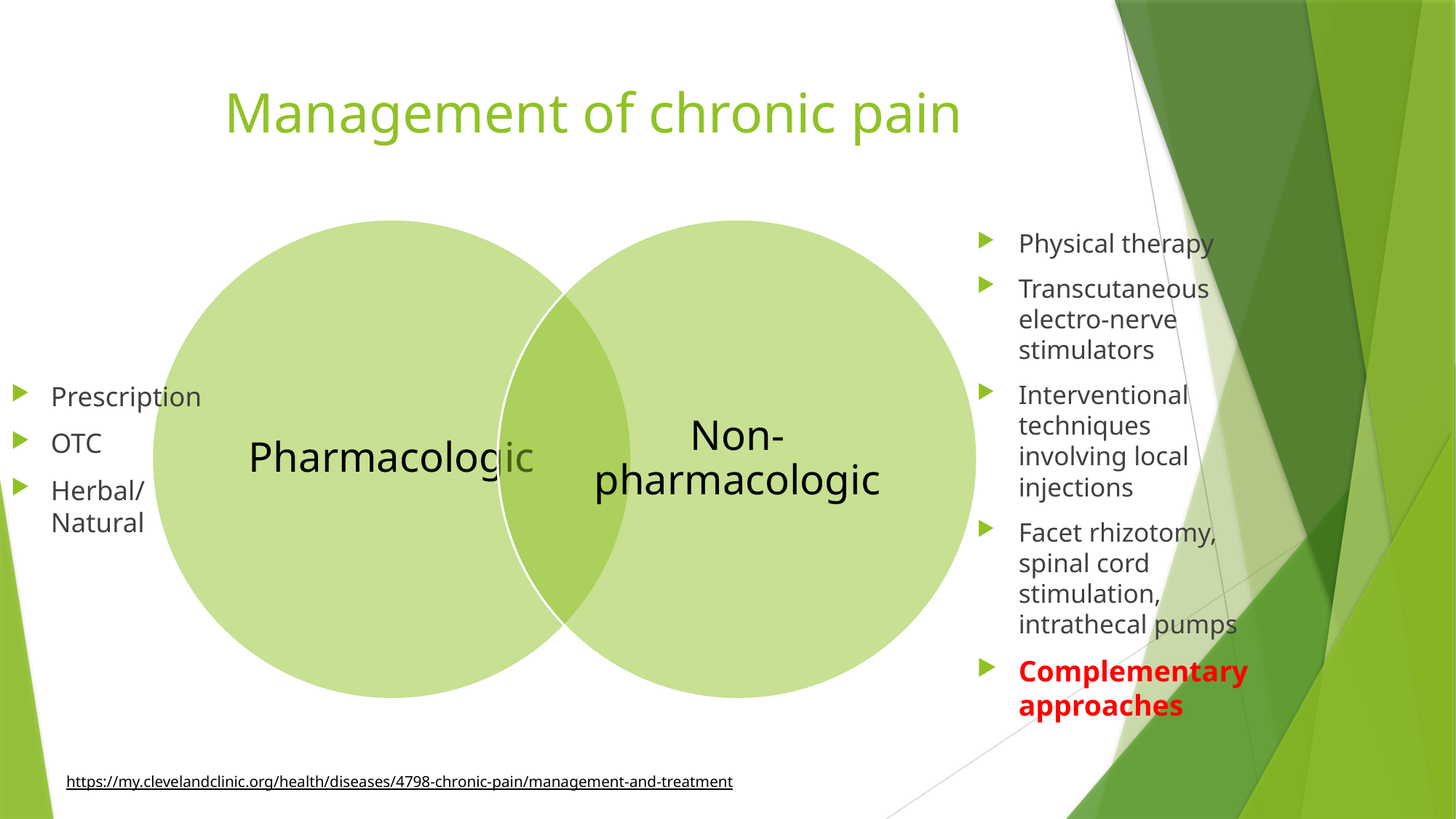

# Management of chronic pain
Physical therapy
Transcutaneous electro-nerve stimulators
Interventional techniques involving local injections
Facet rhizotomy, spinal cord stimulation, intrathecal pumps
Complementary approaches
Prescription
OTC
Herbal/
Natural
https://my.clevelandclinic.org/health/diseases/4798-chronic-pain/management-and-treatment

## Slide 7
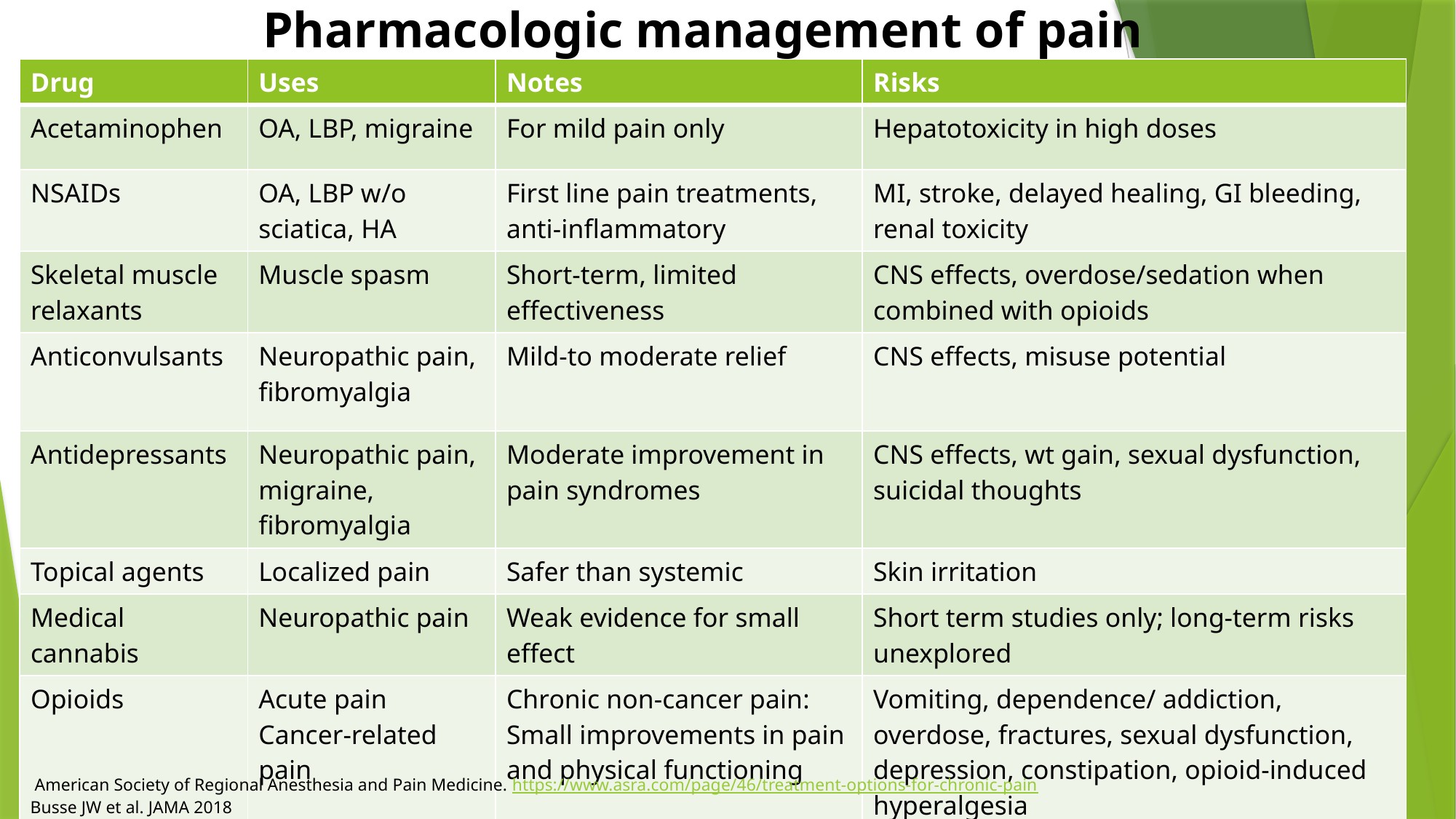

Pharmacologic management of pain
| Drug | Uses | Notes | Risks |
| --- | --- | --- | --- |
| Acetaminophen | OA, LBP, migraine | For mild pain only | Hepatotoxicity in high doses |
| NSAIDs | OA, LBP w/o sciatica, HA | First line pain treatments, anti-inflammatory | MI, stroke, delayed healing, GI bleeding, renal toxicity |
| Skeletal muscle relaxants | Muscle spasm | Short-term, limited effectiveness | CNS effects, overdose/sedation when combined with opioids |
| Anticonvulsants | Neuropathic pain, fibromyalgia | Mild-to moderate relief | CNS effects, misuse potential |
| Antidepressants | Neuropathic pain, migraine, fibromyalgia | Moderate improvement in pain syndromes | CNS effects, wt gain, sexual dysfunction, suicidal thoughts |
| Topical agents | Localized pain | Safer than systemic | Skin irritation |
| Medical cannabis | Neuropathic pain | Weak evidence for small effect | Short term studies only; long-term risks unexplored |
| Opioids | Acute pain Cancer-related pain | Chronic non-cancer pain: Small improvements in pain and physical functioning | Vomiting, dependence/ addiction, overdose, fractures, sexual dysfunction, depression, constipation, opioid-induced hyperalgesia |
 American Society of Regional Anesthesia and Pain Medicine. https://www.asra.com/page/46/treatment-options-for-chronic-pain
Busse JW et al. JAMA 2018

## Slide 8
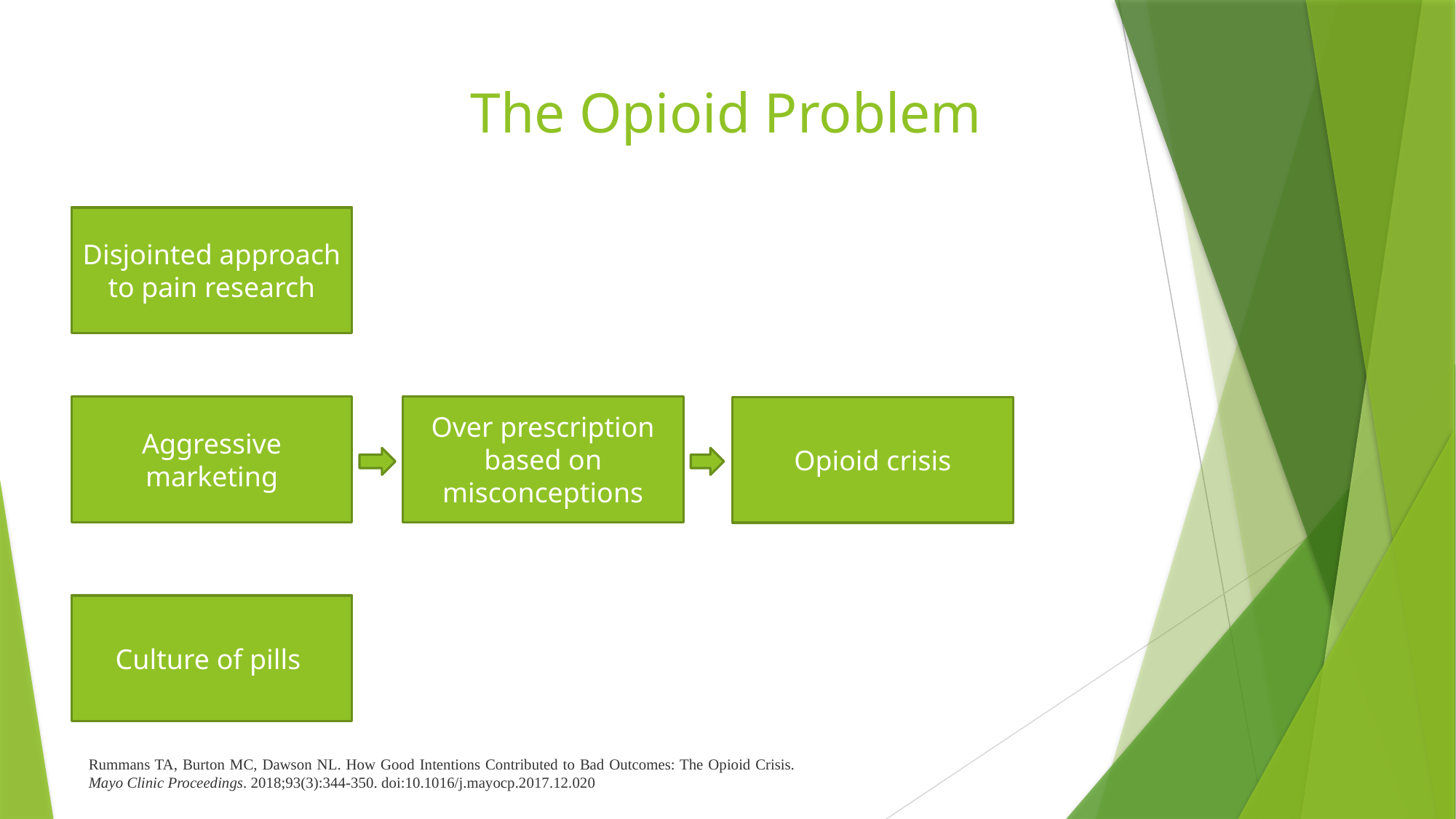

# The Opioid Problem
Disjointed approach to pain research
Aggressive marketing
Over prescription based on misconceptions
Opioid crisis
Culture of pills
Rummans TA, Burton MC, Dawson NL. How Good Intentions Contributed to Bad Outcomes: The Opioid Crisis. Mayo Clinic Proceedings. 2018;93(3):344-350. doi:10.1016/j.mayocp.2017.12.020

## Slide 9
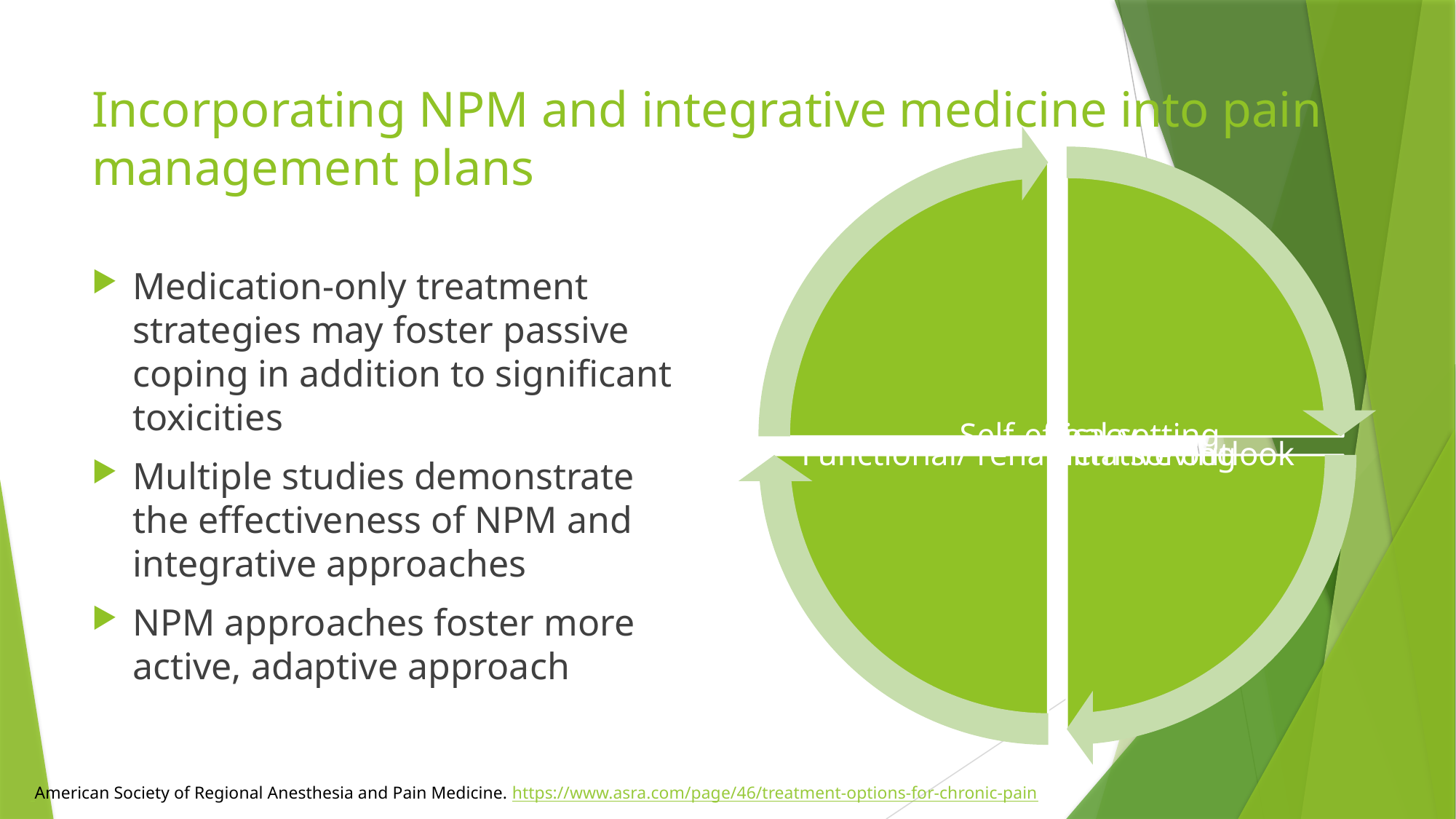

# Incorporating NPM and integrative medicine into pain management plans
Medication-only treatment
strategies may foster passive coping in addition to significant toxicities
Multiple studies demonstrate
the effectiveness of NPM and integrative approaches
NPM approaches foster more active, adaptive approach
 American Society of Regional Anesthesia and Pain Medicine. https://www.asra.com/page/46/treatment-options-for-chronic-pain

## Slide 10
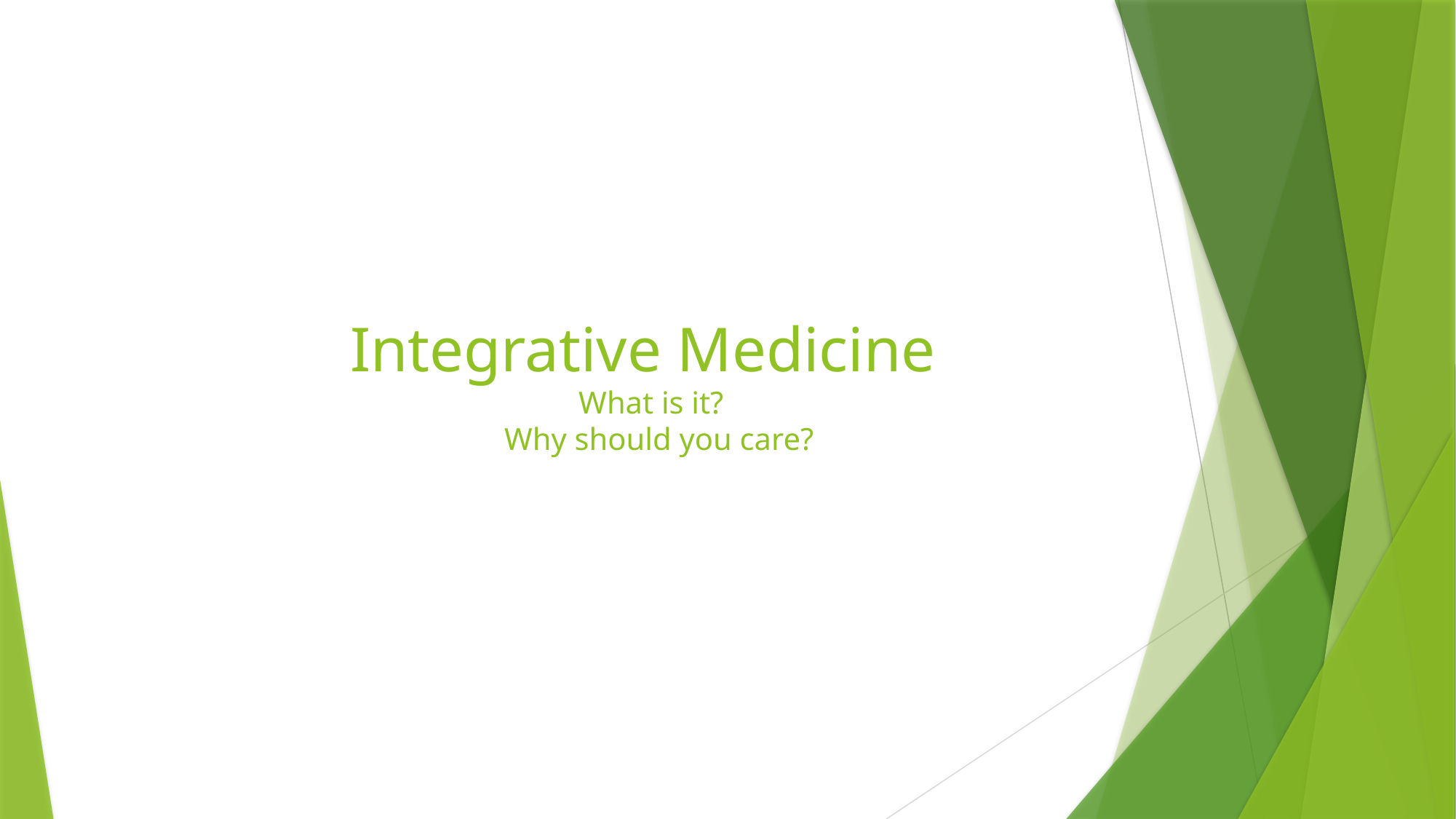

# Integrative Medicine What is it? Why should you care?

## Slide 11
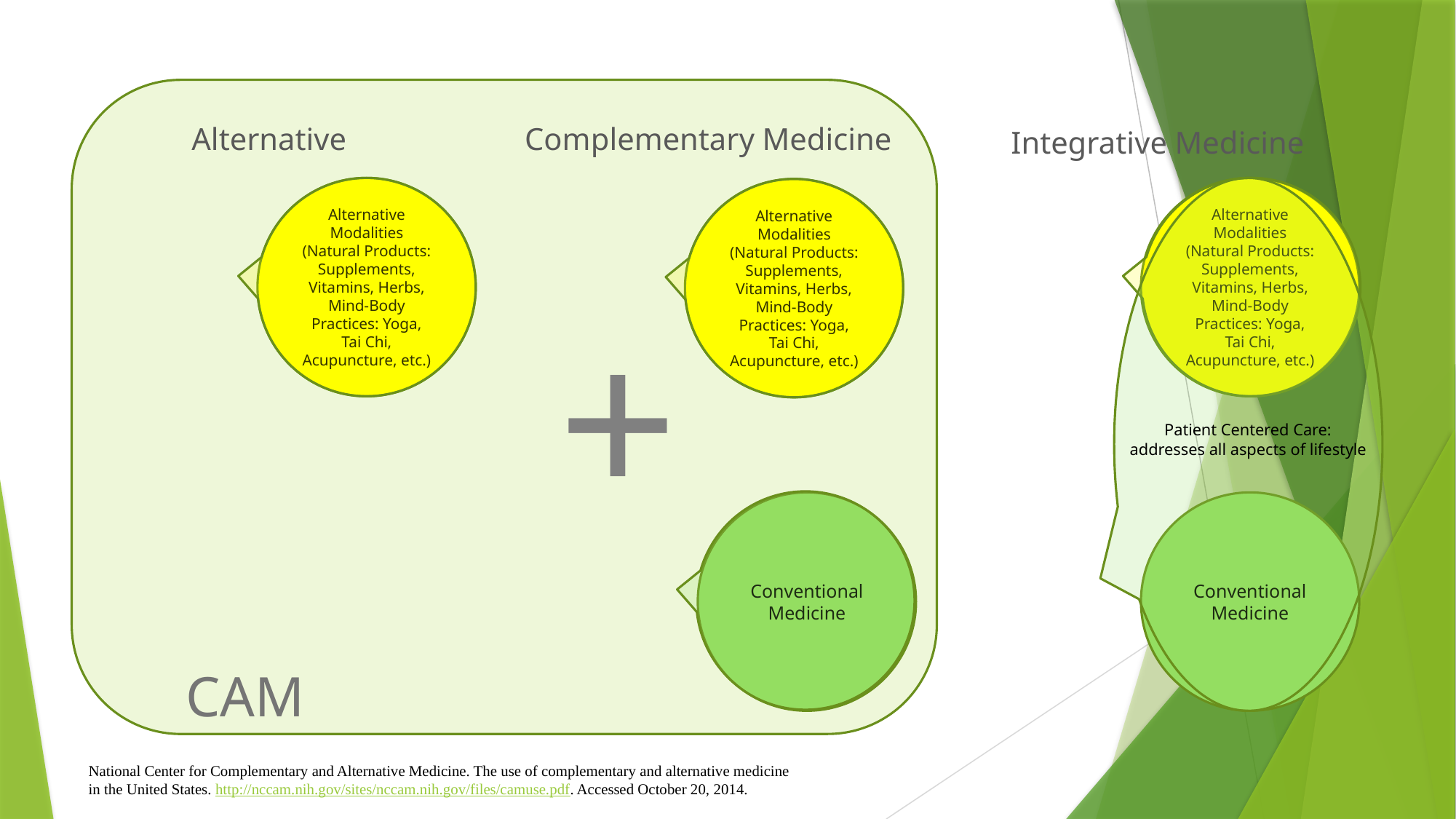

Alternative
Complementary Medicine
Integrative Medicine
Alternative Modalities (Natural Products: Supplements, Vitamins, Herbs, Mind-Body Practices: Yoga, Tai Chi, Acupuncture, etc.)
Alternative Modalities (Natural Products: Supplements, Vitamins, Herbs, Mind-Body Practices: Yoga, Tai Chi, Acupuncture, etc.)
Alternative Modalities (Natural Products: Supplements, Vitamins, Herbs, Mind-Body Practices: Yoga, Tai Chi, Acupuncture, etc.)
+
Patient Centered Care: addresses all aspects of lifestyle
Conventional Medicine
Conventional Medicine
CAM
National Center for Complementary and Alternative Medicine. The use of complementary and alternative medicine in the United States. http://nccam.nih.gov/sites/nccam.nih.gov/files/camuse.pdf. Accessed October 20, 2014.

## Slide 12
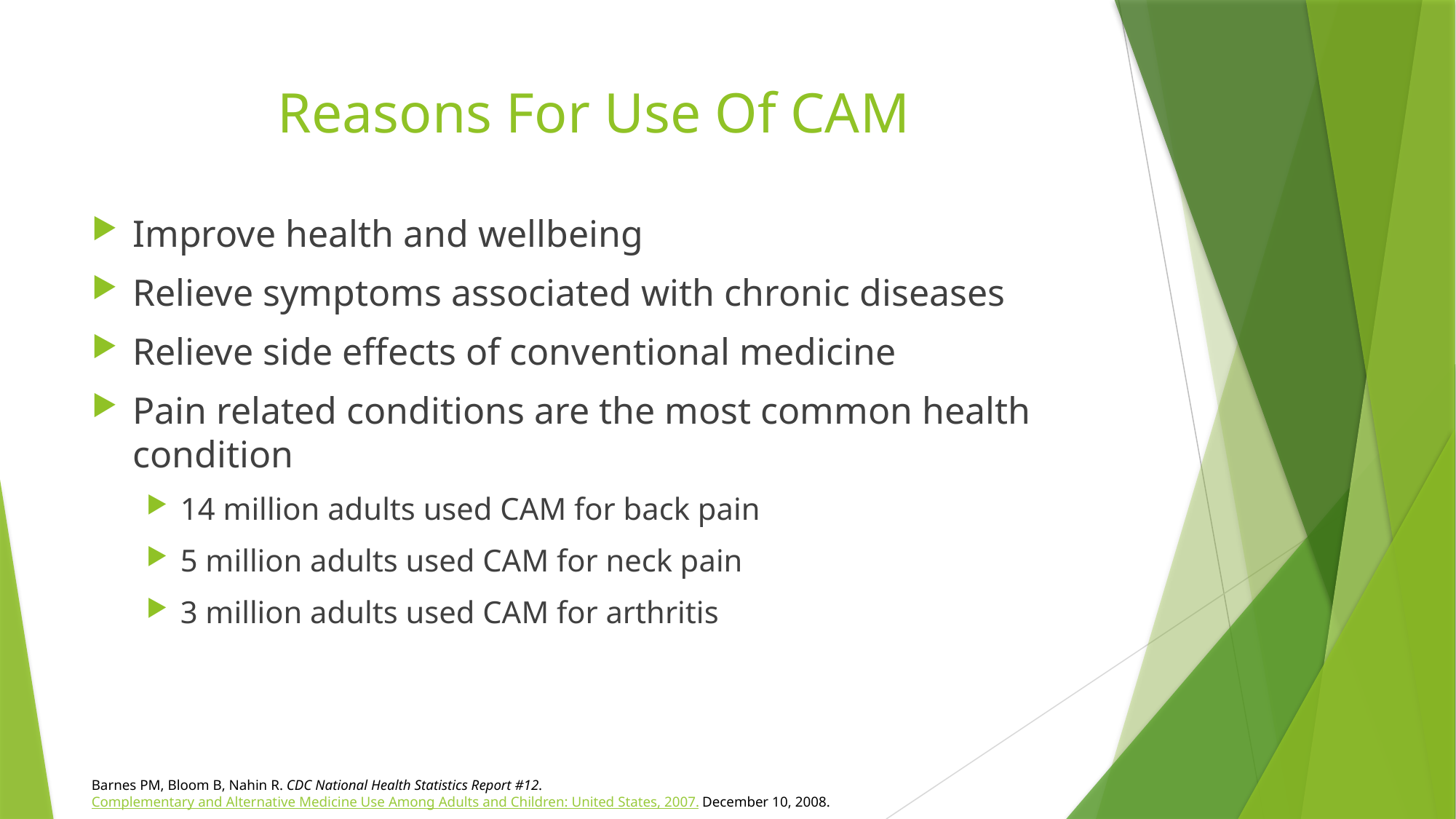

# Reasons For Use Of CAM
Improve health and wellbeing
Relieve symptoms associated with chronic diseases
Relieve side effects of conventional medicine
Pain related conditions are the most common health condition
14 million adults used CAM for back pain
5 million adults used CAM for neck pain
3 million adults used CAM for arthritis
Barnes PM, Bloom B, Nahin R. CDC National Health Statistics Report #12. Complementary and Alternative Medicine Use Among Adults and Children: United States, 2007. December 10, 2008.

## Slide 13
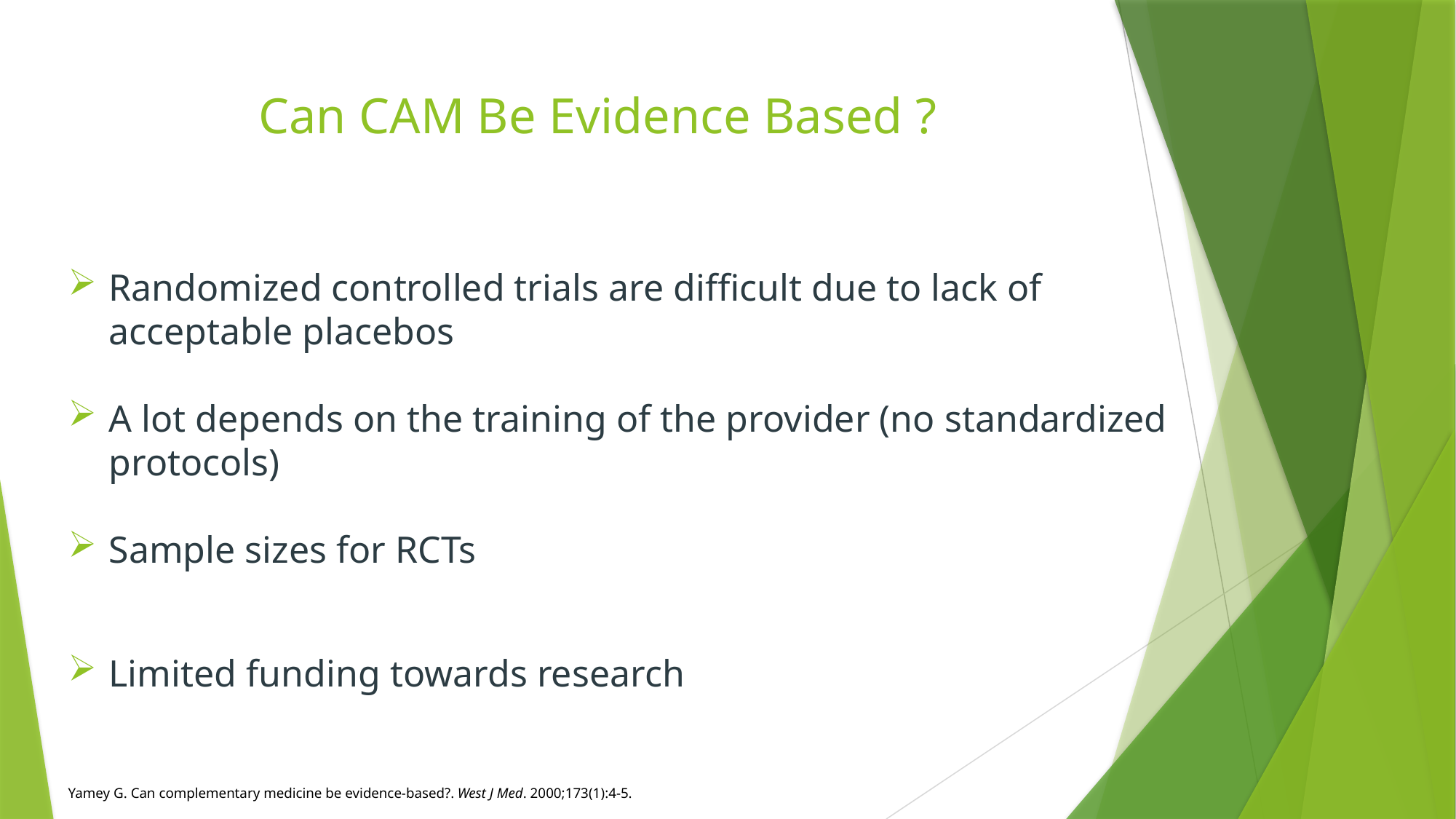

# Can CAM Be Evidence Based ?
Randomized controlled trials are difficult due to lack of acceptable placebos
A lot depends on the training of the provider (no standardized protocols)
Sample sizes for RCTs
Limited funding towards research
Yamey G. Can complementary medicine be evidence-based?. West J Med. 2000;173(1):4-5.

## Slide 14
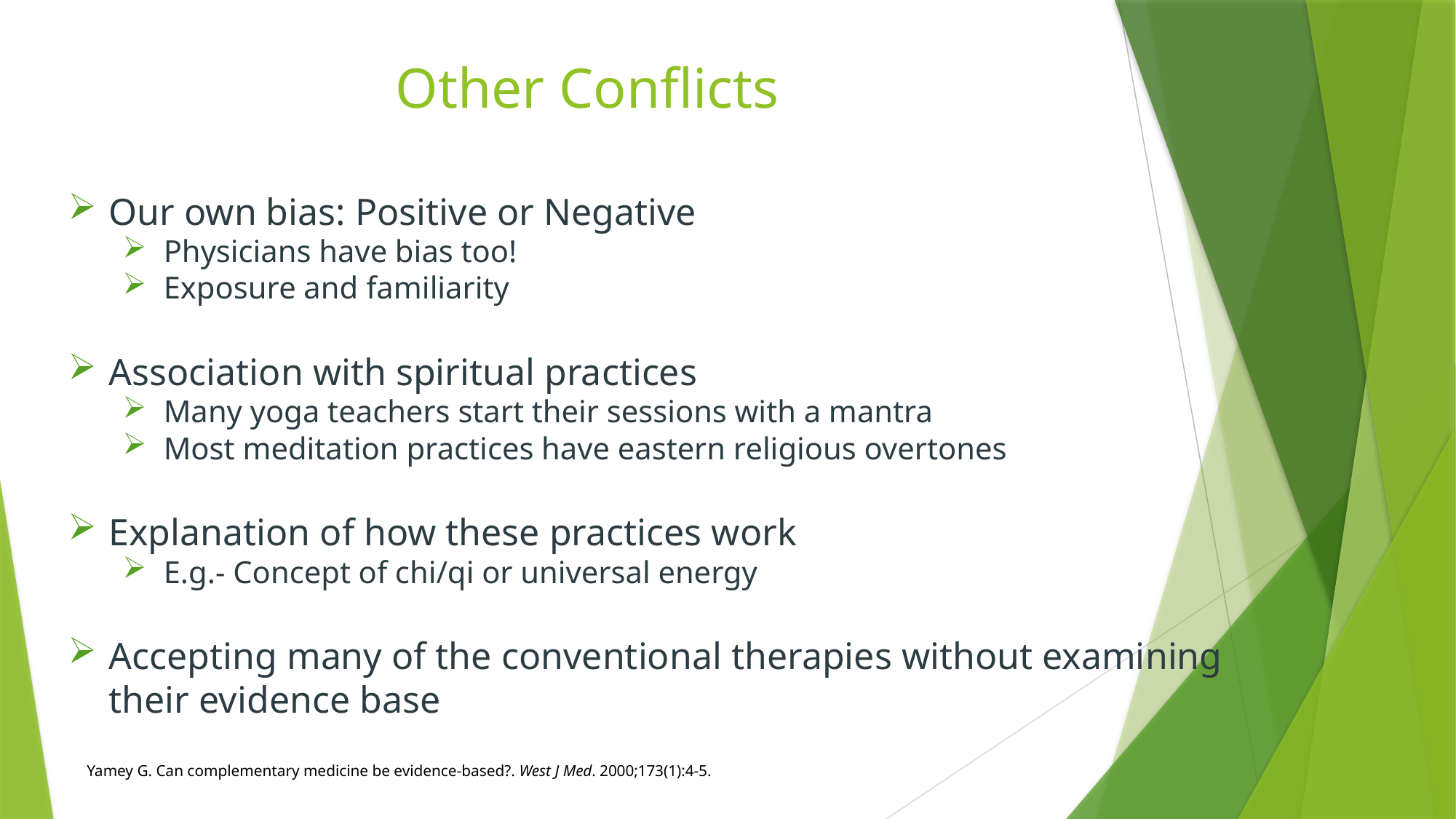

# Other Conflicts
Our own bias: Positive or Negative
Physicians have bias too!
Exposure and familiarity
Association with spiritual practices
Many yoga teachers start their sessions with a mantra
Most meditation practices have eastern religious overtones
Explanation of how these practices work
E.g.- Concept of chi/qi or universal energy
Accepting many of the conventional therapies without examining their evidence base
Yamey G. Can complementary medicine be evidence-based?. West J Med. 2000;173(1):4-5.

## Slide 15
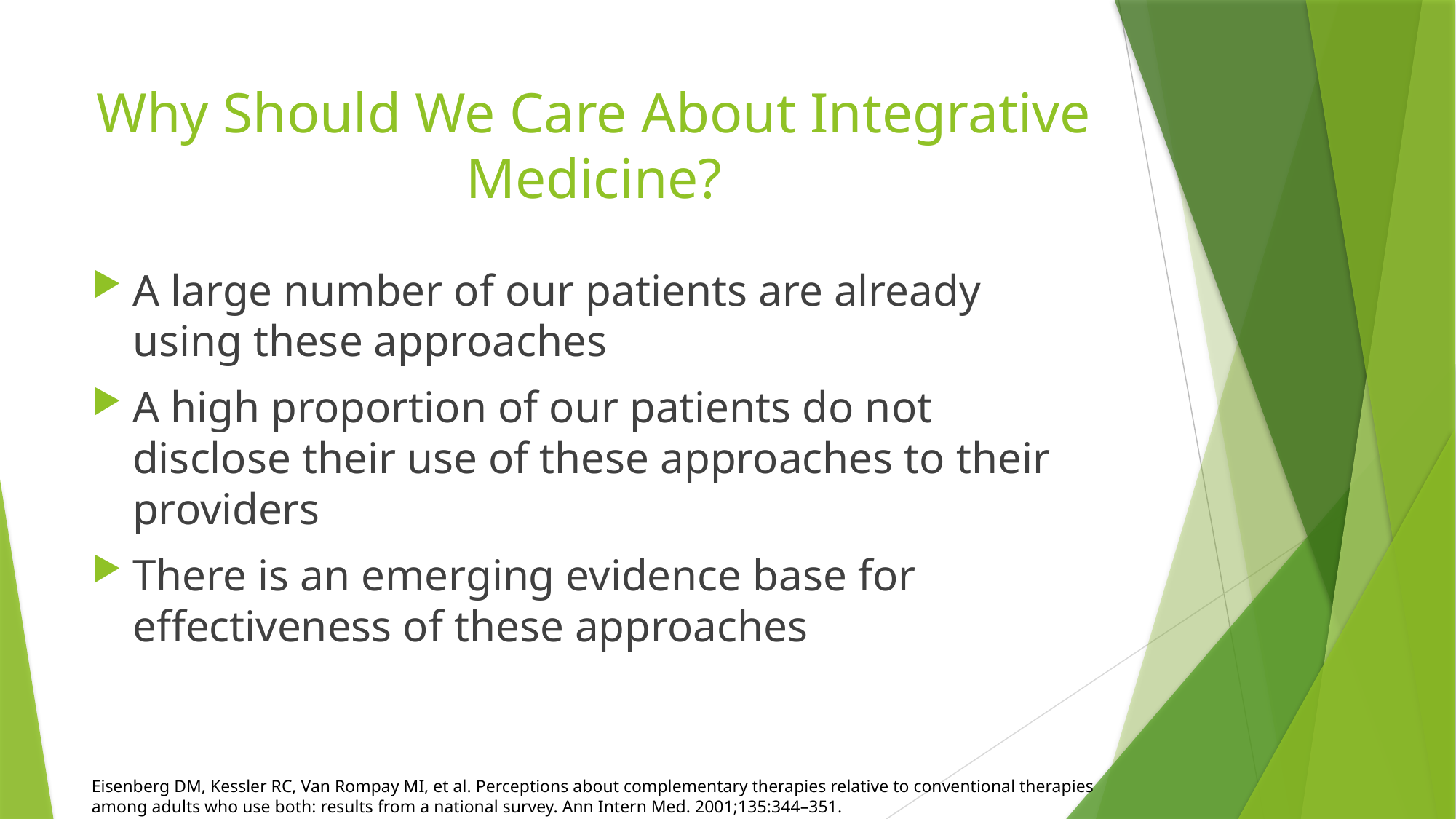

# Why Should We Care About Integrative Medicine?
A large number of our patients are already using these approaches
A high proportion of our patients do not disclose their use of these approaches to their providers
There is an emerging evidence base for effectiveness of these approaches
Eisenberg DM, Kessler RC, Van Rompay MI, et al. Perceptions about complementary therapies relative to conventional therapies among adults who use both: results from a national survey. Ann Intern Med. 2001;135:344–351.

## Slide 16
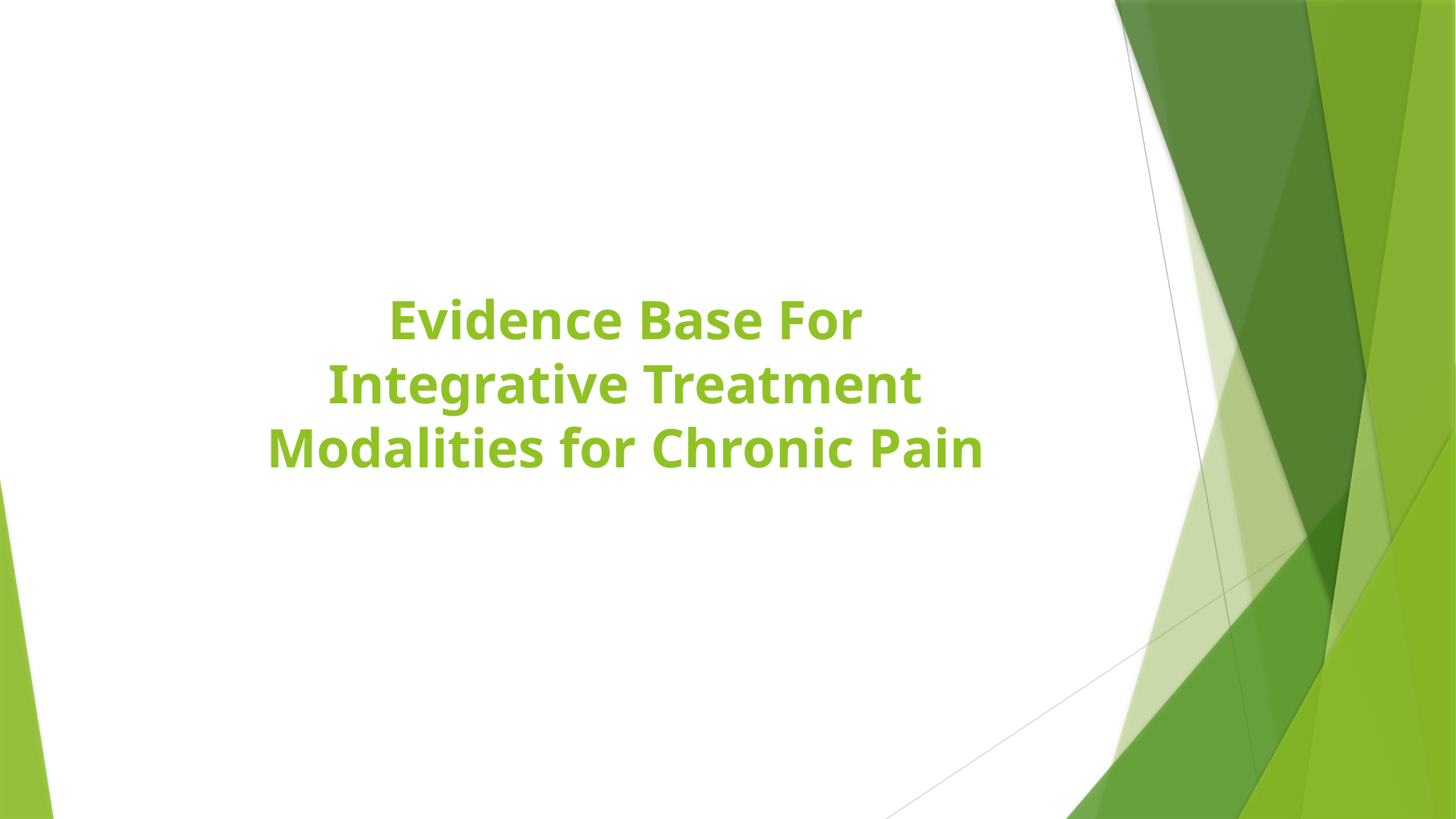

# Evidence Base For Integrative Treatment Modalities for Chronic Pain

## Slide 17
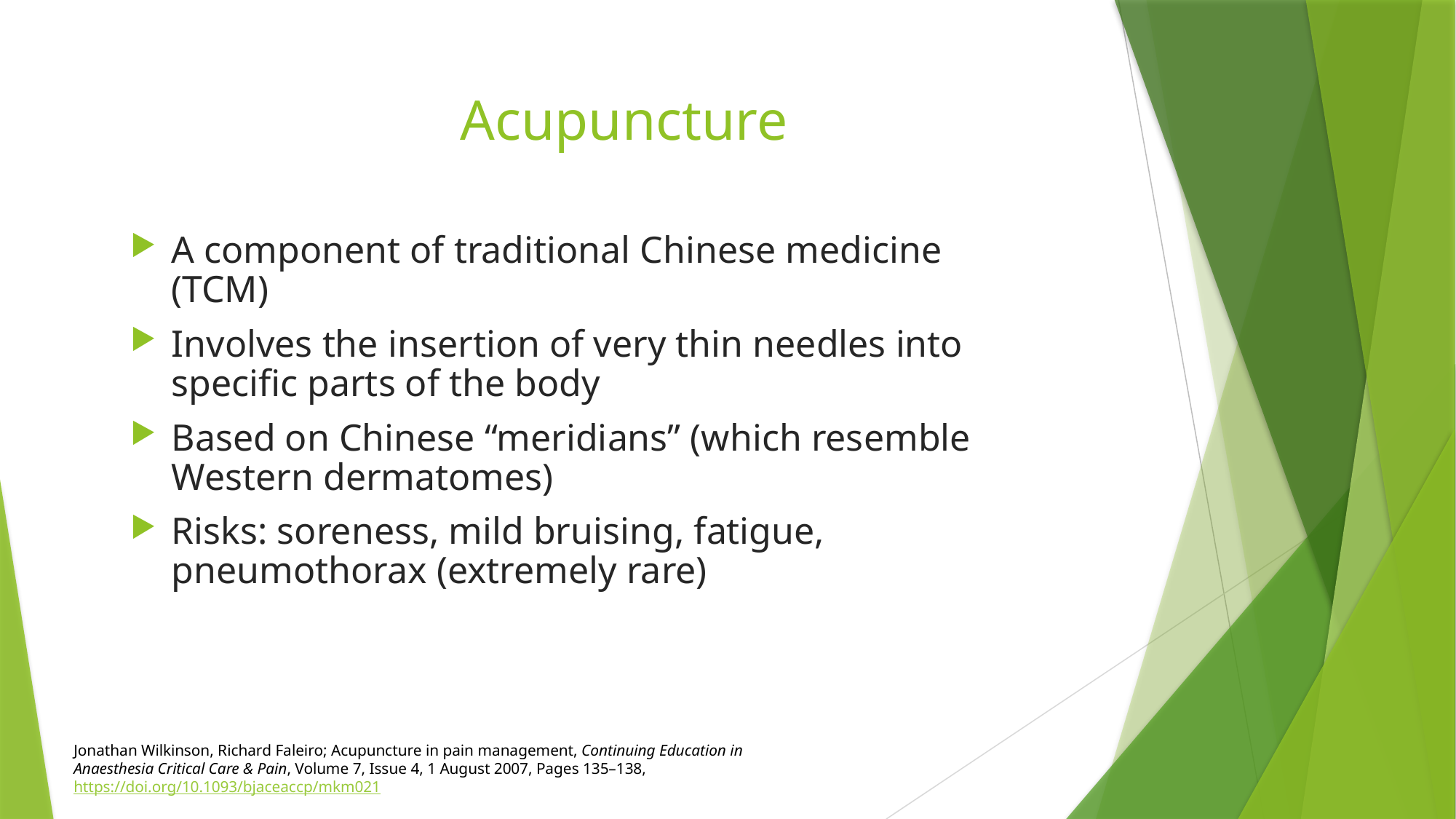

# Acupuncture
A component of traditional Chinese medicine (TCM)
Involves the insertion of very thin needles into specific parts of the body
Based on Chinese “meridians” (which resemble Western dermatomes)
Risks: soreness, mild bruising, fatigue, pneumothorax (extremely rare)
Jonathan Wilkinson, Richard Faleiro; Acupuncture in pain management, Continuing Education in Anaesthesia Critical Care & Pain, Volume 7, Issue 4, 1 August 2007, Pages 135–138, https://doi.org/10.1093/bjaceaccp/mkm021

## Slide 18
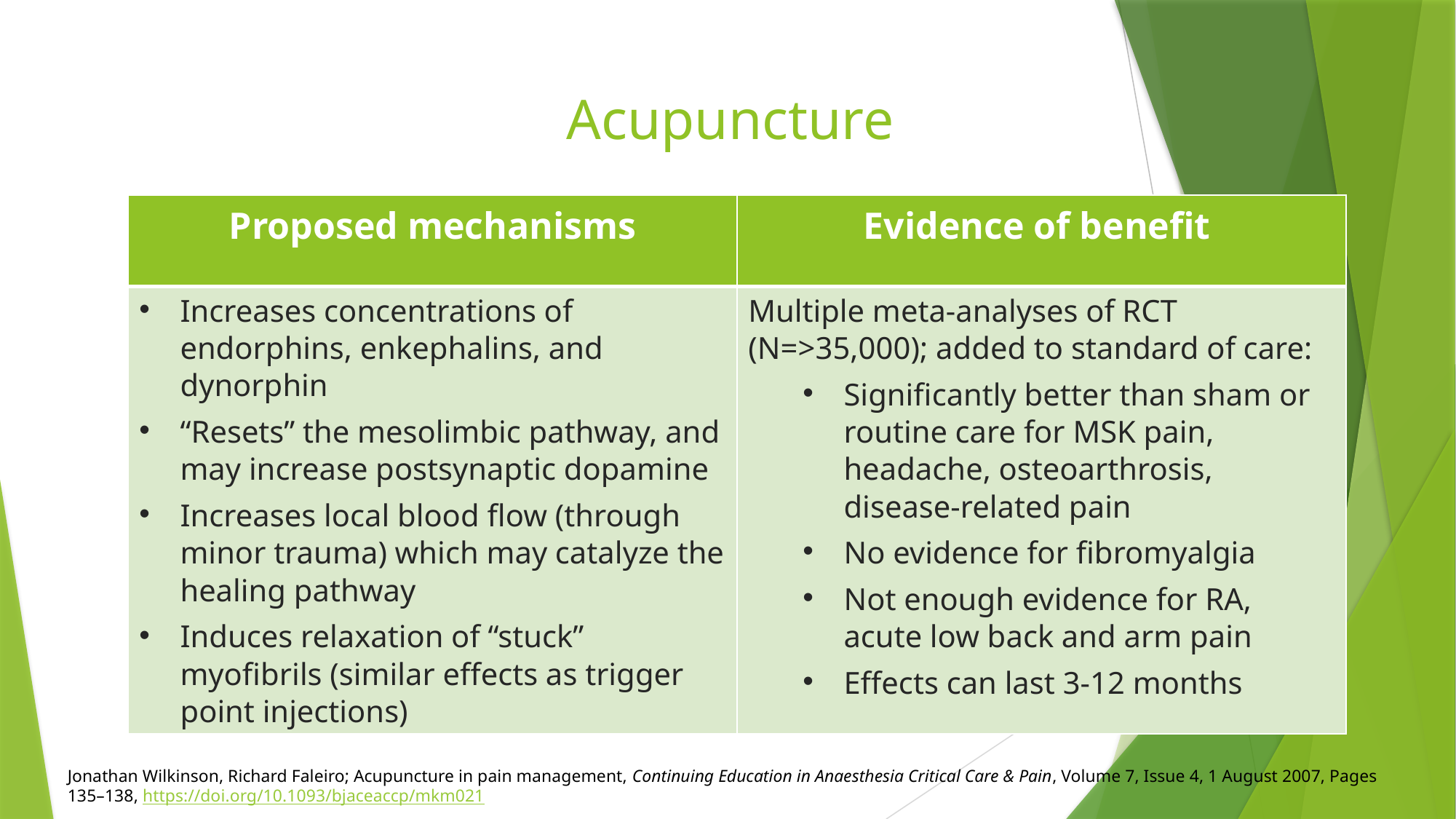

# Acupuncture
| Proposed mechanisms | Evidence of benefit |
| --- | --- |
| Increases concentrations of endorphins, enkephalins, and dynorphin “Resets” the mesolimbic pathway, and may increase postsynaptic dopamine Increases local blood flow (through minor trauma) which may catalyze the healing pathway Induces relaxation of “stuck” myofibrils (similar effects as trigger point injections) | Multiple meta-analyses of RCT (N=>35,000); added to standard of care: Significantly better than sham or routine care for MSK pain, headache, osteoarthrosis, disease-related pain No evidence for fibromyalgia Not enough evidence for RA, acute low back and arm pain Effects can last 3-12 months |
Jonathan Wilkinson, Richard Faleiro; Acupuncture in pain management, Continuing Education in Anaesthesia Critical Care & Pain, Volume 7, Issue 4, 1 August 2007, Pages 135–138, https://doi.org/10.1093/bjaceaccp/mkm021

## Slide 19
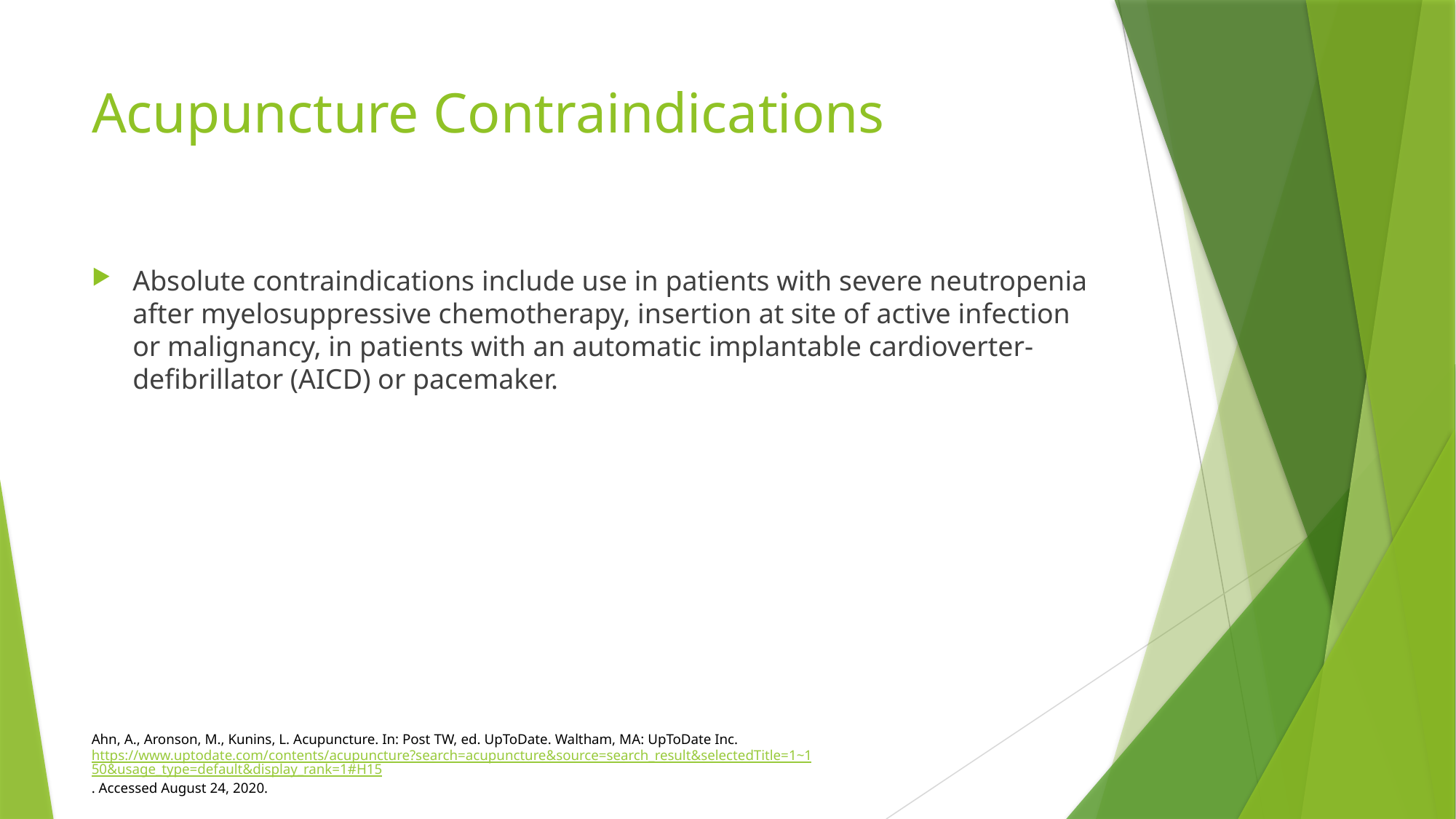

# Acupuncture Contraindications
Absolute contraindications include use in patients with severe neutropenia after myelosuppressive chemotherapy, insertion at site of active infection or malignancy, in patients with an automatic implantable cardioverter-defibrillator (AICD) or pacemaker.
Ahn, A., Aronson, M., Kunins, L. Acupuncture. In: Post TW, ed. UpToDate. Waltham, MA: UpToDate Inc. https://www.uptodate.com/contents/acupuncture?search=acupuncture&source=search_result&selectedTitle=1~150&usage_type=default&display_rank=1#H15. Accessed August 24, 2020.

## Slide 20
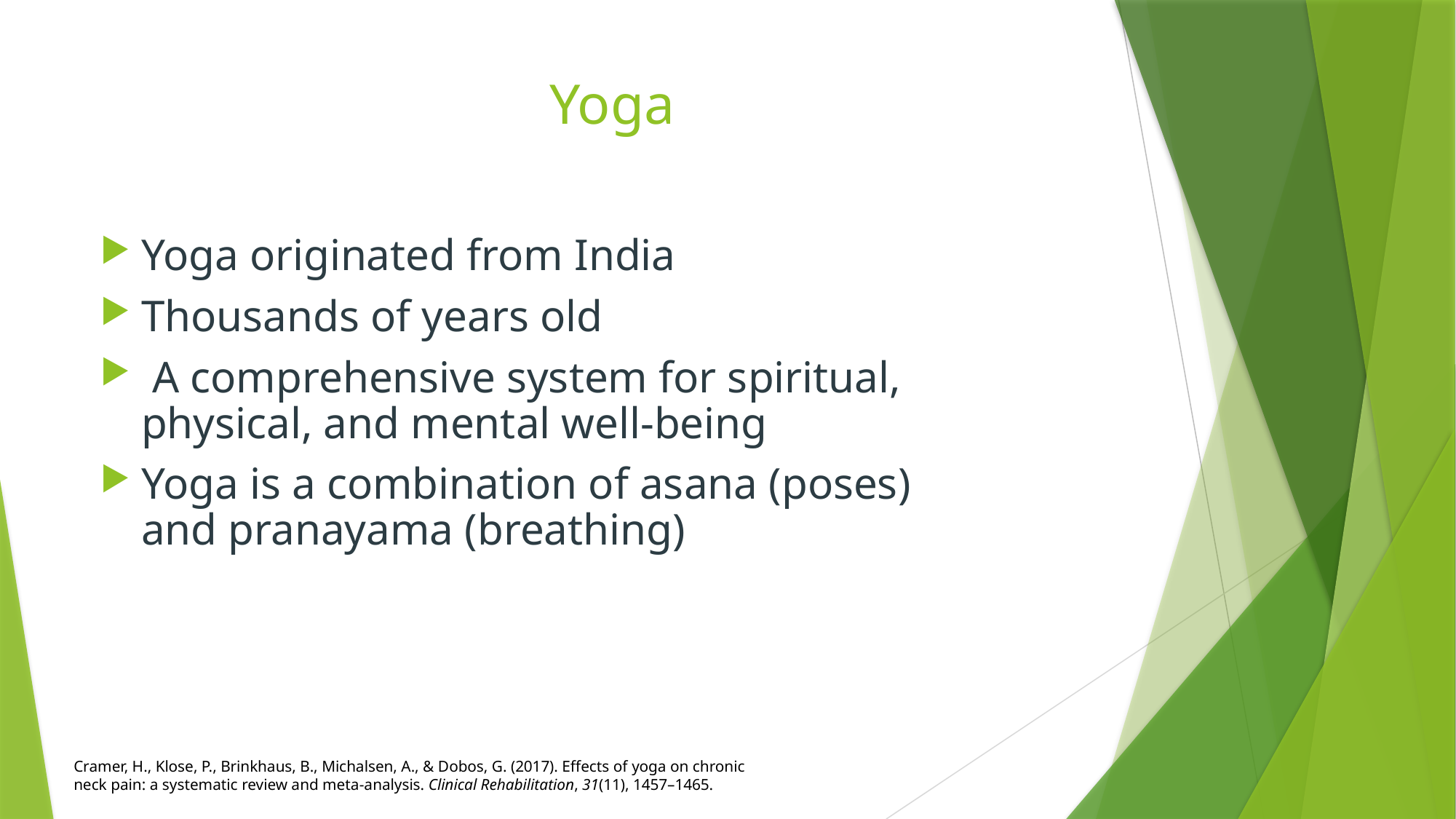

# Yoga
Yoga originated from India
Thousands of years old
 A comprehensive system for spiritual, physical, and mental well-being
Yoga is a combination of asana (poses) and pranayama (breathing)
Cramer, H., Klose, P., Brinkhaus, B., Michalsen, A., & Dobos, G. (2017). Effects of yoga on chronic neck pain: a systematic review and meta-analysis. Clinical Rehabilitation, 31(11), 1457–1465.

## Slide 21
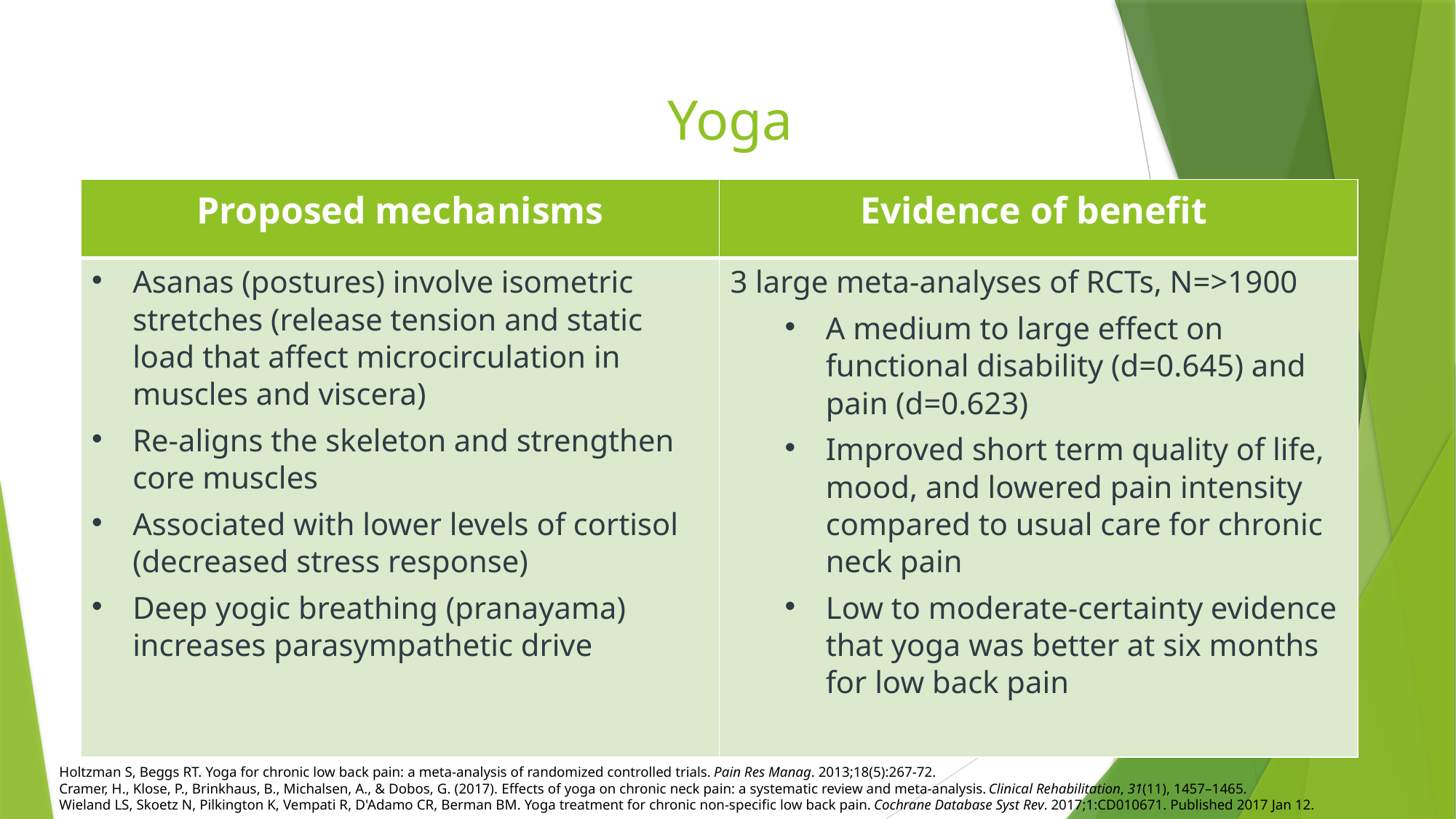

# Yoga
| Proposed mechanisms | Evidence of benefit |
| --- | --- |
| Asanas (postures) involve isometric stretches (release tension and static load that affect microcirculation in muscles and viscera) Re-aligns the skeleton and strengthen core muscles Associated with lower levels of cortisol (decreased stress response) Deep yogic breathing (pranayama) increases parasympathetic drive | 3 large meta-analyses of RCTs, N=>1900 A medium to large effect on functional disability (d=0.645) and pain (d=0.623) Improved short term quality of life, mood, and lowered pain intensity compared to usual care for chronic neck pain Low to moderate-certainty evidence that yoga was better at six months for low back pain |
Holtzman S, Beggs RT. Yoga for chronic low back pain: a meta-analysis of randomized controlled trials. Pain Res Manag. 2013;18(5):267-72.
Cramer, H., Klose, P., Brinkhaus, B., Michalsen, A., & Dobos, G. (2017). Effects of yoga on chronic neck pain: a systematic review and meta-analysis. Clinical Rehabilitation, 31(11), 1457–1465.
Wieland LS, Skoetz N, Pilkington K, Vempati R, D'Adamo CR, Berman BM. Yoga treatment for chronic non-specific low back pain. Cochrane Database Syst Rev. 2017;1:CD010671. Published 2017 Jan 12.

## Slide 22
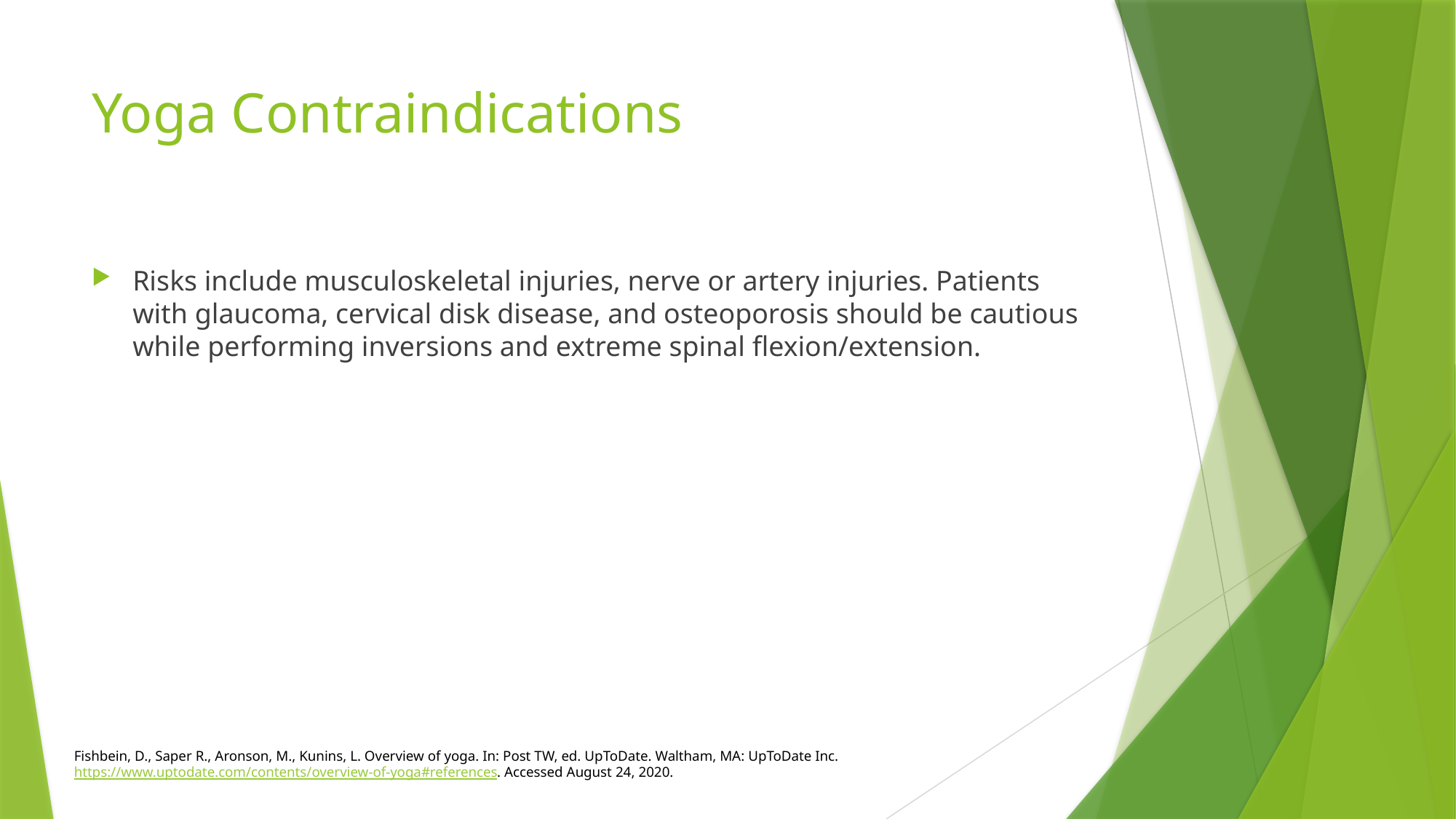

# Yoga Contraindications
Risks include musculoskeletal injuries, nerve or artery injuries. Patients with glaucoma, cervical disk disease, and osteoporosis should be cautious while performing inversions and extreme spinal flexion/extension.
Fishbein, D., Saper R., Aronson, M., Kunins, L. Overview of yoga. In: Post TW, ed. UpToDate. Waltham, MA: UpToDate Inc. https://www.uptodate.com/contents/overview-of-yoga#references. Accessed August 24, 2020.

## Slide 23
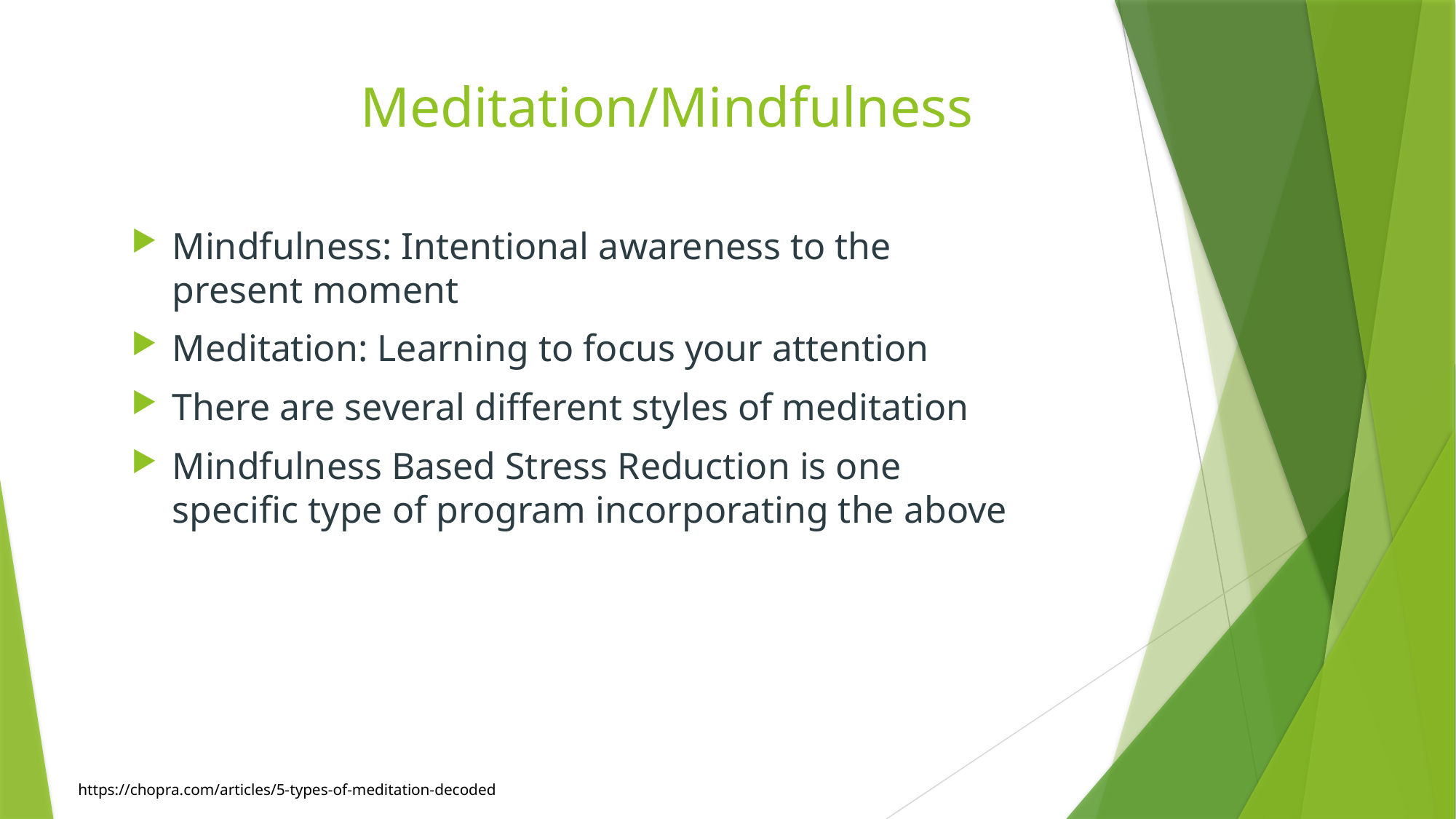

# Meditation/Mindfulness
Mindfulness: Intentional awareness to the present moment
Meditation: Learning to focus your attention
There are several different styles of meditation
Mindfulness Based Stress Reduction is one specific type of program incorporating the above
https://chopra.com/articles/5-types-of-meditation-decoded

## Slide 24
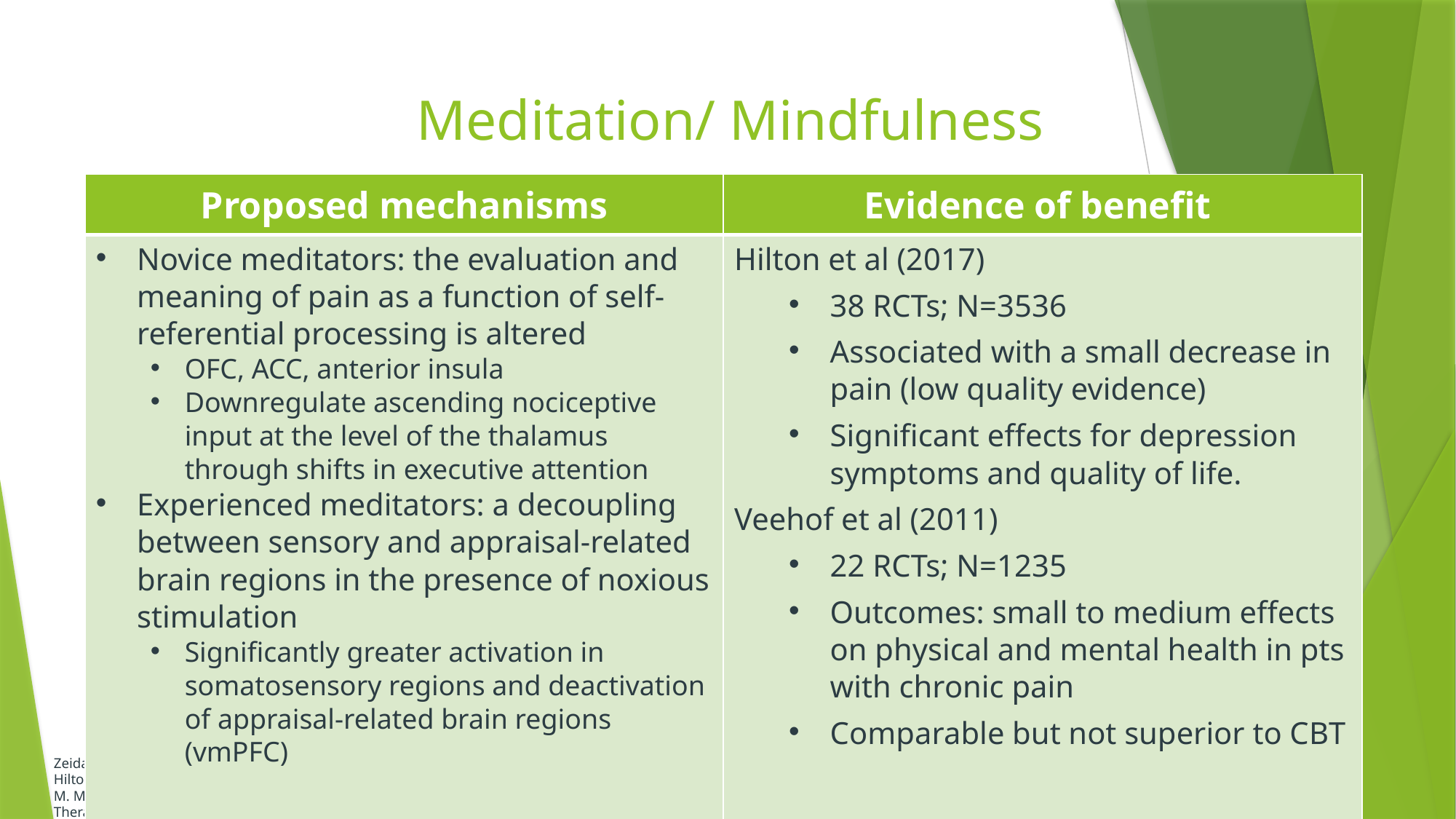

# Meditation/ Mindfulness
| Proposed mechanisms | Evidence of benefit |
| --- | --- |
| Novice meditators: the evaluation and meaning of pain as a function of self-referential processing is altered OFC, ACC, anterior insula Downregulate ascending nociceptive input at the level of the thalamus through shifts in executive attention Experienced meditators: a decoupling between sensory and appraisal-related brain regions in the presence of noxious stimulation Significantly greater activation in somatosensory regions and deactivation of appraisal-related brain regions (vmPFC) | Hilton et al (2017) 38 RCTs; N=3536 Associated with a small decrease in pain (low quality evidence) Significant effects for depression symptoms and quality of life. Veehof et al (2011) 22 RCTs; N=1235 Outcomes: small to medium effects on physical and mental health in pts with chronic pain Comparable but not superior to CBT |
Zeidan F, Vago DR. Mindfulness meditation-based pain relief: a mechanistic account. Ann N Y Acad Sci. 2016;1373(1):114-27.
Hilton L, Hempel S, Ewing BA, et al. Mindfulness Meditation for Chronic Pain: Systematic Review and Meta-analysis. Ann Behav Med. 2016;51(2):199-213.
M. M. Veehof, H. R. Trompetter, E. T. Bohlmeijer & K. M. G. Schreurs (2016) Acceptance- and mindfulness-based interventions for the treatment of chronic pain: a meta-analytic review,Cognitive Behaviour Therapy, 45:1, 5-31.

## Slide 25
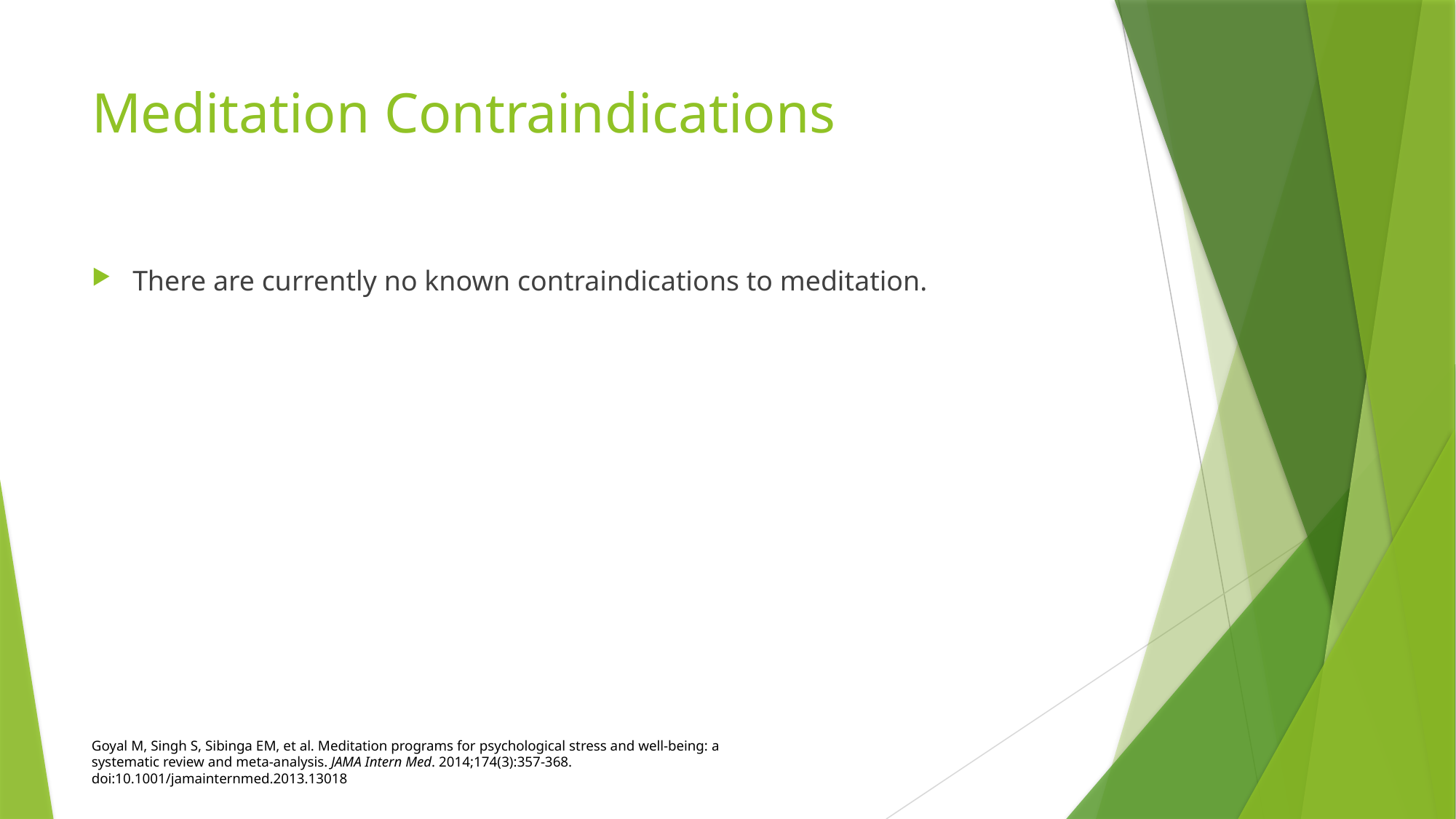

# Meditation Contraindications
There are currently no known contraindications to meditation.
Goyal M, Singh S, Sibinga EM, et al. Meditation programs for psychological stress and well-being: a systematic review and meta-analysis. JAMA Intern Med. 2014;174(3):357-368. doi:10.1001/jamainternmed.2013.13018

## Slide 26
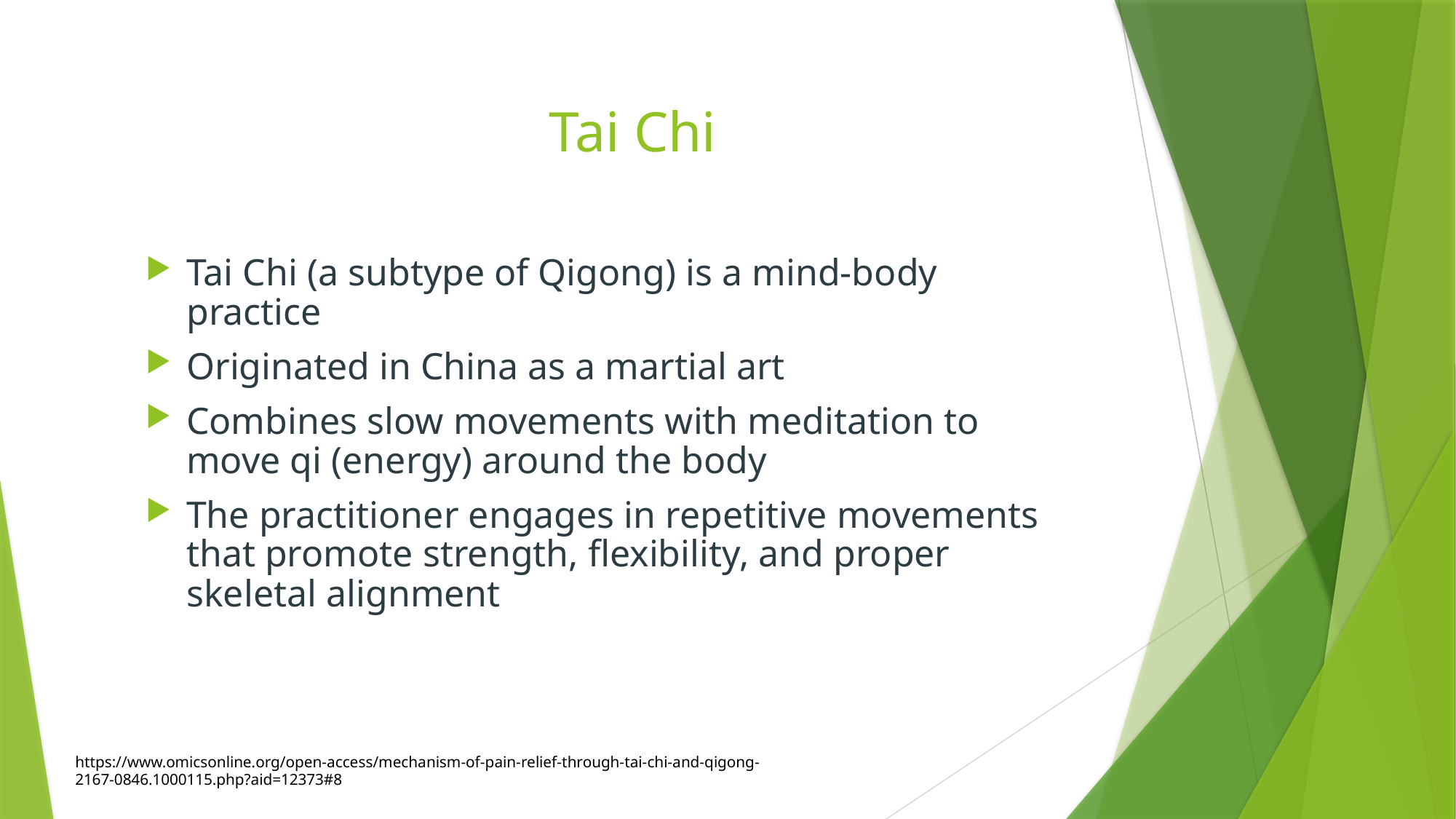

# Tai Chi
Tai Chi (a subtype of Qigong) is a mind-body practice
Originated in China as a martial art
Combines slow movements with meditation to move qi (energy) around the body
The practitioner engages in repetitive movements that promote strength, flexibility, and proper skeletal alignment
https://www.omicsonline.org/open-access/mechanism-of-pain-relief-through-tai-chi-and-qigong-2167-0846.1000115.php?aid=12373#8

## Slide 27
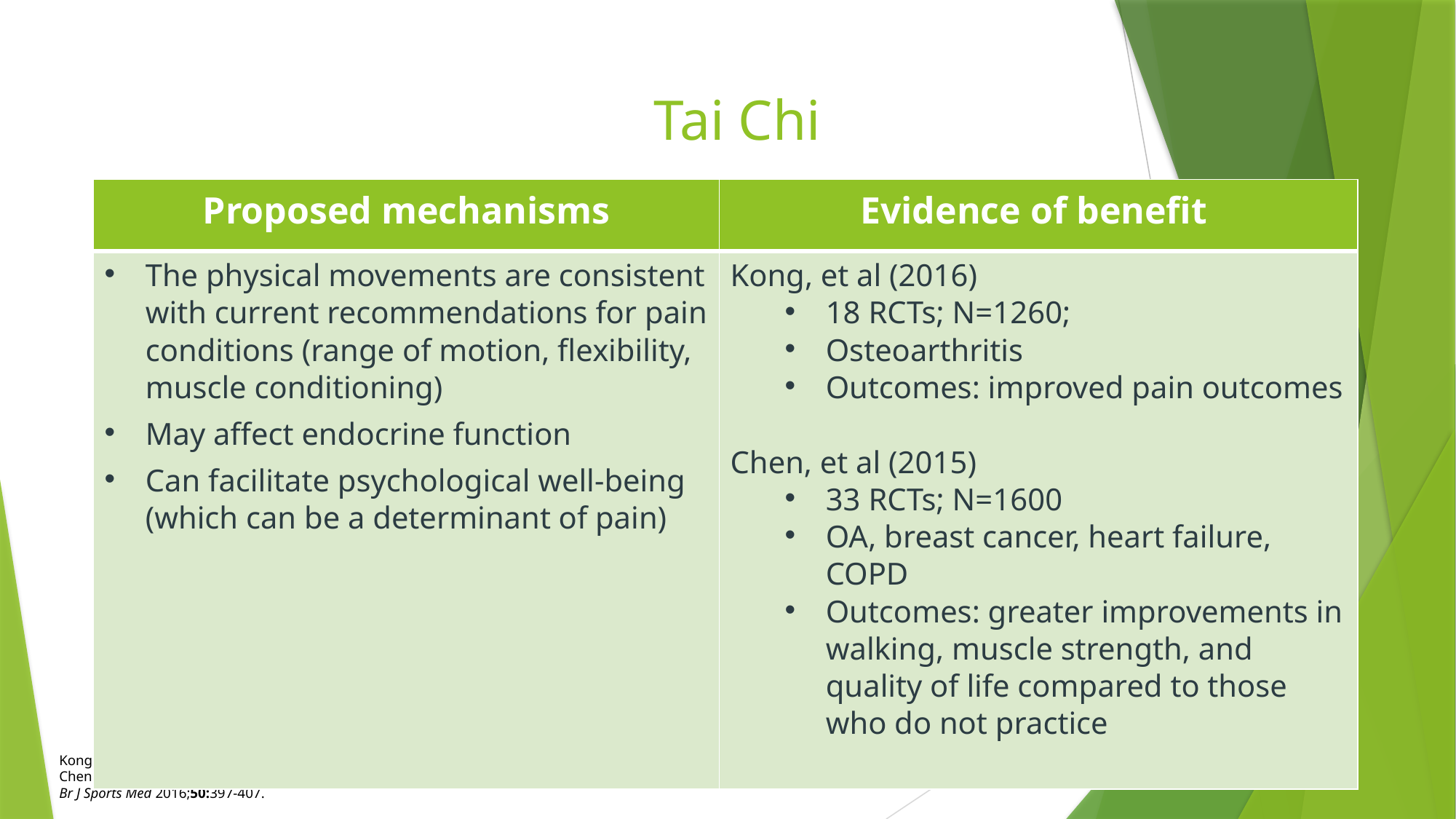

# Tai Chi
| Proposed mechanisms | Evidence of benefit |
| --- | --- |
| The physical movements are consistent with current recommendations for pain conditions (range of motion, flexibility, muscle conditioning) May affect endocrine function Can facilitate psychological well-being (which can be a determinant of pain) | Kong, et al (2016) 18 RCTs; N=1260; Osteoarthritis Outcomes: improved pain outcomes Chen, et al (2015) 33 RCTs; N=1600 OA, breast cancer, heart failure, COPD Outcomes: greater improvements in walking, muscle strength, and quality of life compared to those who do not practice |
Kong LJ, Lauche R, Klose P, et al. Tai Chi for Chronic Pain Conditions: A Systematic Review and Meta-analysis of Randomized Controlled Trials. Sci Rep. 2016;6:25325.
Chen Y, Hunt MA, Campbell KL, et al. The effect of Tai Chi on four chronic conditions—cancer, osteoarthritis, heart failure and chronic obstructive pulmonary disease: a systematic review and meta-analyses
Br J Sports Med 2016;50:397-407.

## Slide 28
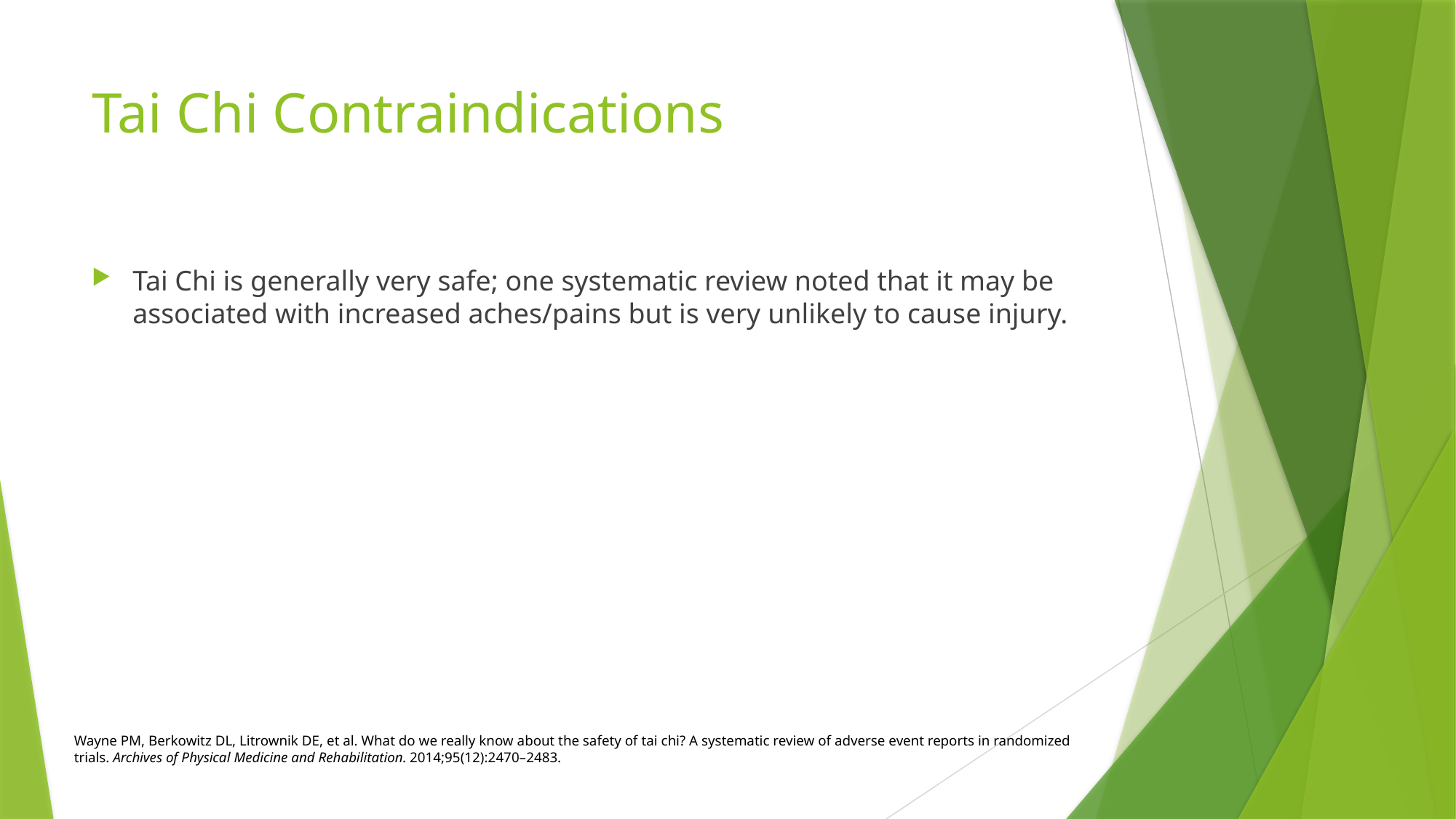

# Tai Chi Contraindications
Tai Chi is generally very safe; one systematic review noted that it may be associated with increased aches/pains but is very unlikely to cause injury.
Wayne PM, Berkowitz DL, Litrownik DE, et al. What do we really know about the safety of tai chi? A systematic review of adverse event reports in randomized trials. Archives of Physical Medicine and Rehabilitation. 2014;95(12):2470–2483.

## Slide 29
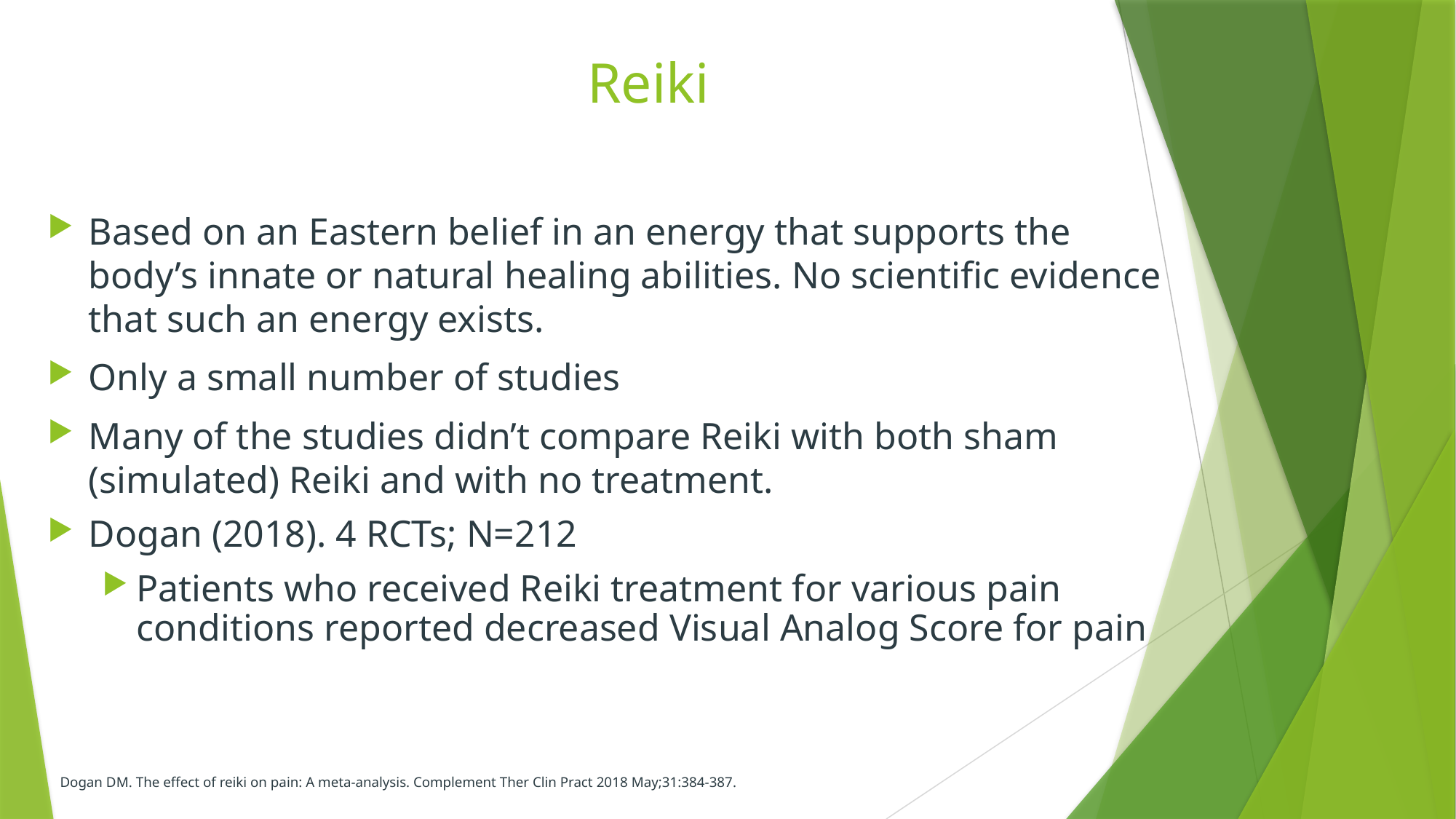

# Reiki
Based on an Eastern belief in an energy that supports the body’s innate or natural healing abilities. No scientific evidence that such an energy exists.
Only a small number of studies
Many of the studies didn’t compare Reiki with both sham (simulated) Reiki and with no treatment.
Dogan (2018). 4 RCTs; N=212
Patients who received Reiki treatment for various pain conditions reported decreased Visual Analog Score for pain
Dogan DM. The effect of reiki on pain: A meta-analysis. Complement Ther Clin Pract 2018 May;31:384-387.

## Slide 30
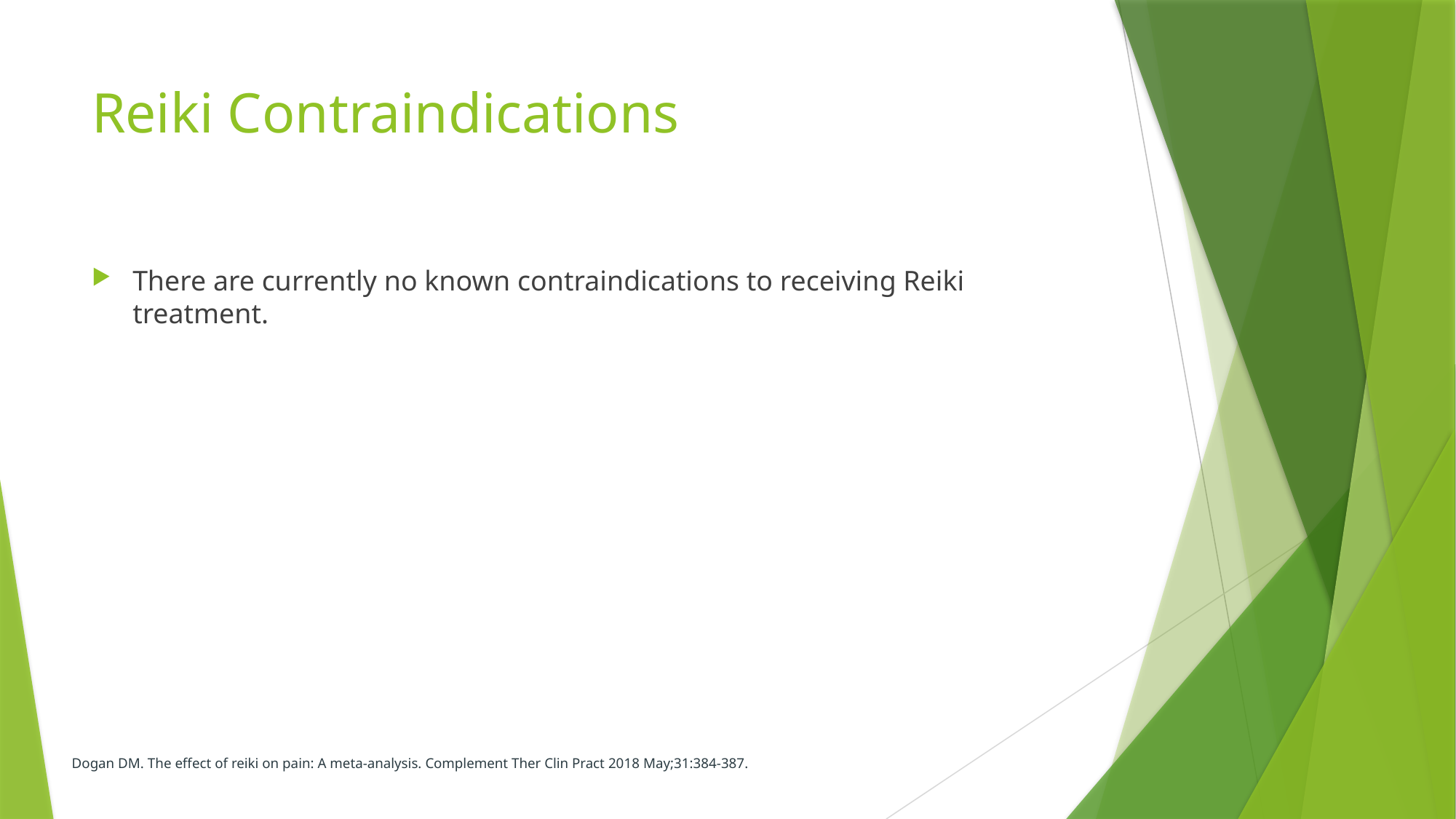

# Reiki Contraindications
There are currently no known contraindications to receiving Reiki treatment.
Dogan DM. The effect of reiki on pain: A meta-analysis. Complement Ther Clin Pract 2018 May;31:384-387.

## Slide 31
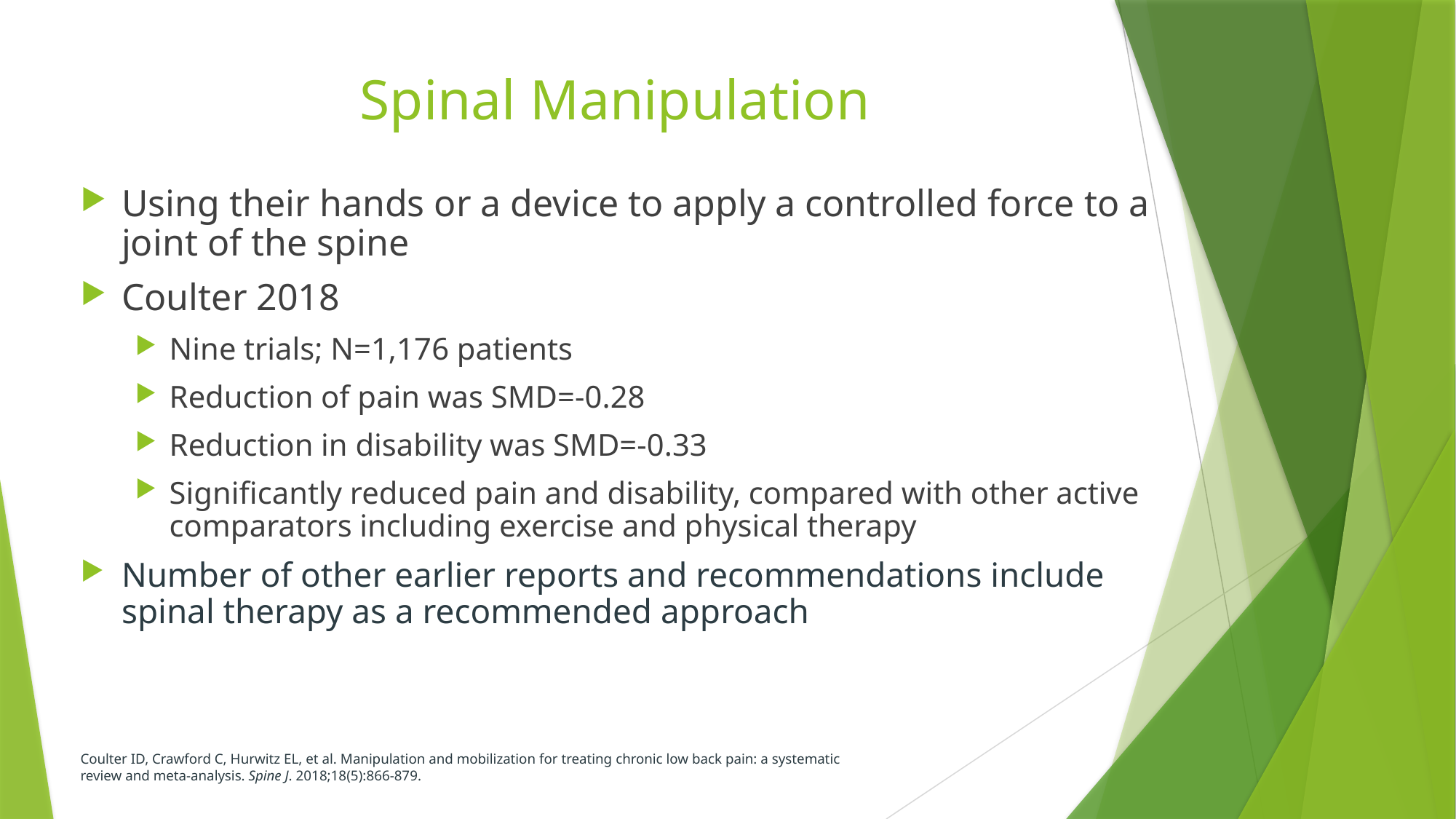

# Spinal Manipulation
Using their hands or a device to apply a controlled force to a joint of the spine
Coulter 2018
Nine trials; N=1,176 patients
Reduction of pain was SMD=-0.28
Reduction in disability was SMD=-0.33
Significantly reduced pain and disability, compared with other active comparators including exercise and physical therapy
Number of other earlier reports and recommendations include spinal therapy as a recommended approach
Coulter ID, Crawford C, Hurwitz EL, et al. Manipulation and mobilization for treating chronic low back pain: a systematic review and meta-analysis. Spine J. 2018;18(5):866-879.

## Slide 32
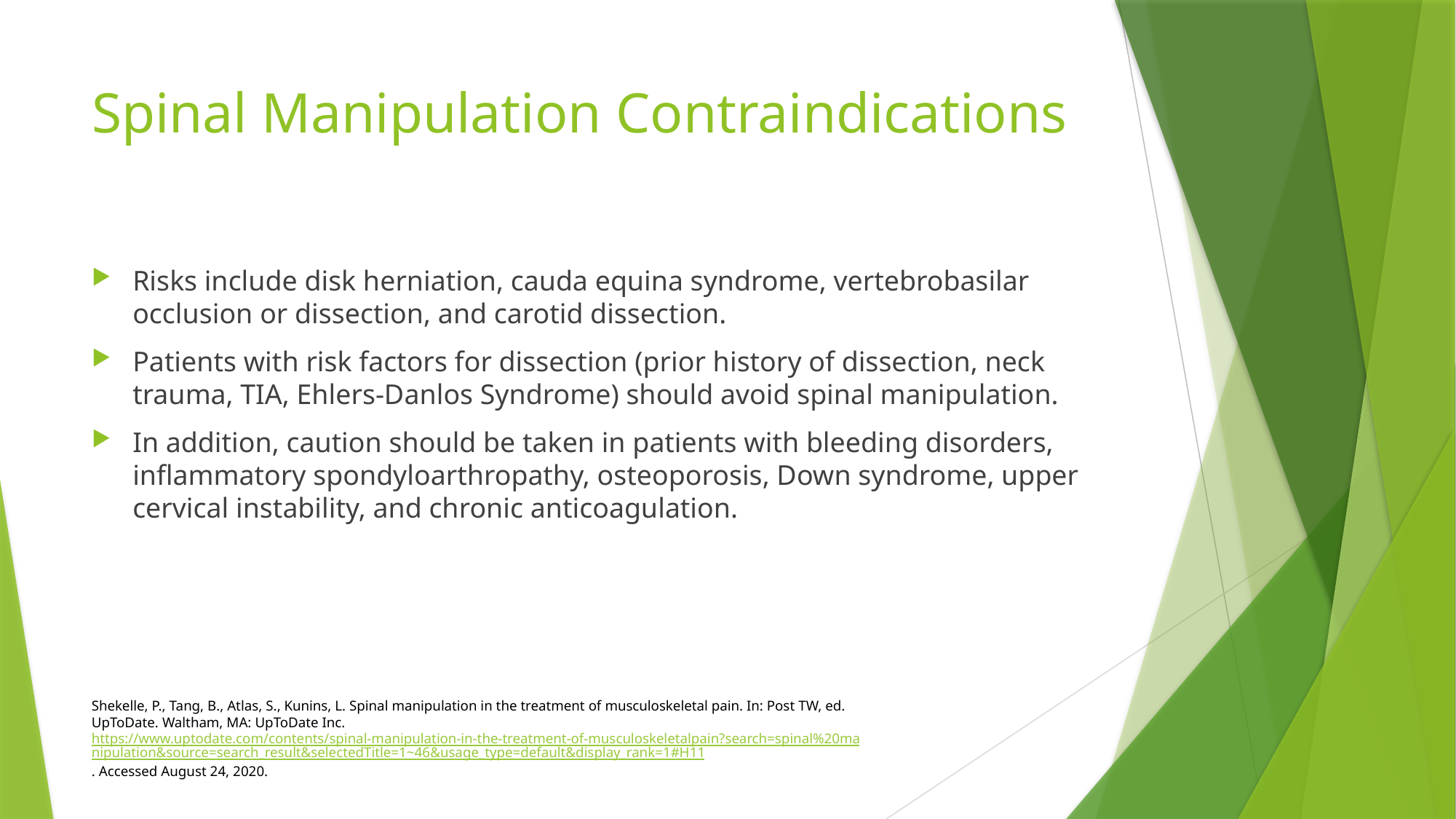

# Spinal Manipulation Contraindications
Risks include disk herniation, cauda equina syndrome, vertebrobasilar occlusion or dissection, and carotid dissection.
Patients with risk factors for dissection (prior history of dissection, neck trauma, TIA, Ehlers-Danlos Syndrome) should avoid spinal manipulation.
In addition, caution should be taken in patients with bleeding disorders, inflammatory spondyloarthropathy, osteoporosis, Down syndrome, upper cervical instability, and chronic anticoagulation.
Shekelle, P., Tang, B., Atlas, S., Kunins, L. Spinal manipulation in the treatment of musculoskeletal pain. In: Post TW, ed. UpToDate. Waltham, MA: UpToDate Inc. https://www.uptodate.com/contents/spinal-manipulation-in-the-treatment-of-musculoskeletalpain?search=spinal%20manipulation&source=search_result&selectedTitle=1~46&usage_type=default&display_rank=1#H11. Accessed August 24, 2020.

## Slide 33
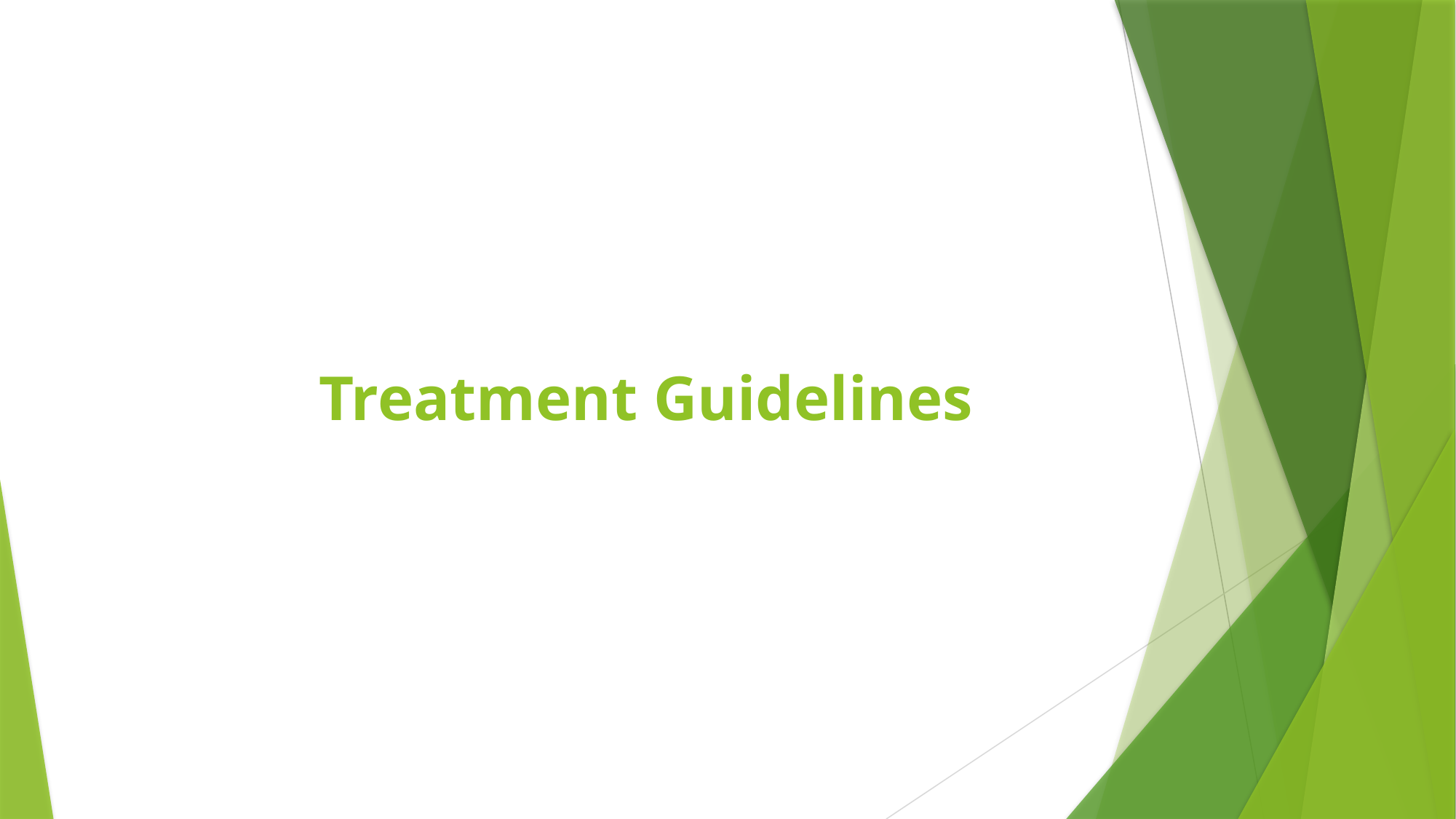

# Treatment Guidelines

## Slide 34
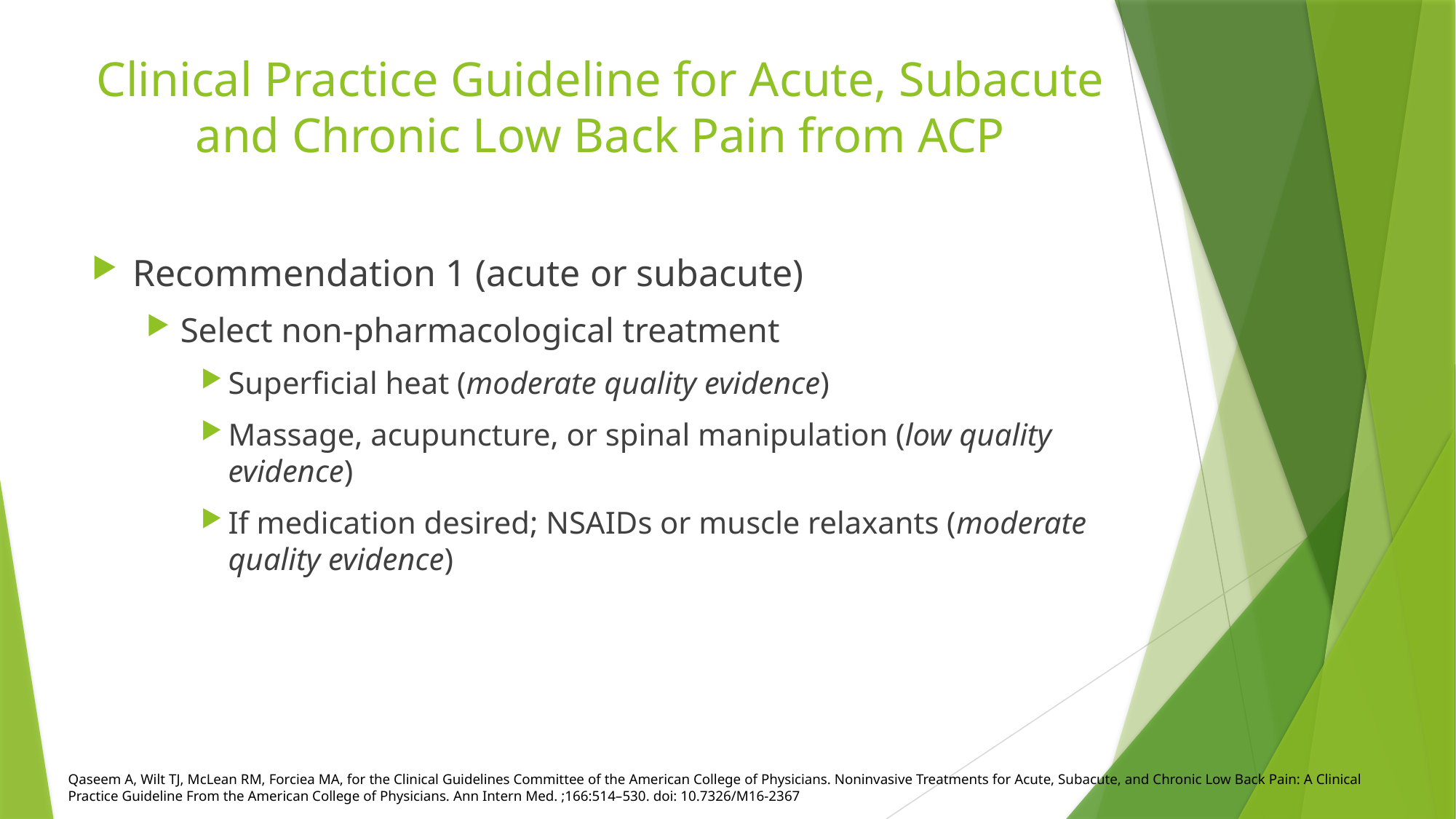

# Clinical Practice Guideline for Acute, Subacute and Chronic Low Back Pain from ACP
Recommendation 1 (acute or subacute)
Select non-pharmacological treatment
Superficial heat (moderate quality evidence)
Massage, acupuncture, or spinal manipulation (low quality evidence)
If medication desired; NSAIDs or muscle relaxants (moderate quality evidence)
Qaseem A, Wilt TJ, McLean RM, Forciea MA, for the Clinical Guidelines Committee of the American College of Physicians. Noninvasive Treatments for Acute, Subacute, and Chronic Low Back Pain: A Clinical Practice Guideline From the American College of Physicians. Ann Intern Med. ;166:514–530. doi: 10.7326/M16-2367

## Slide 35
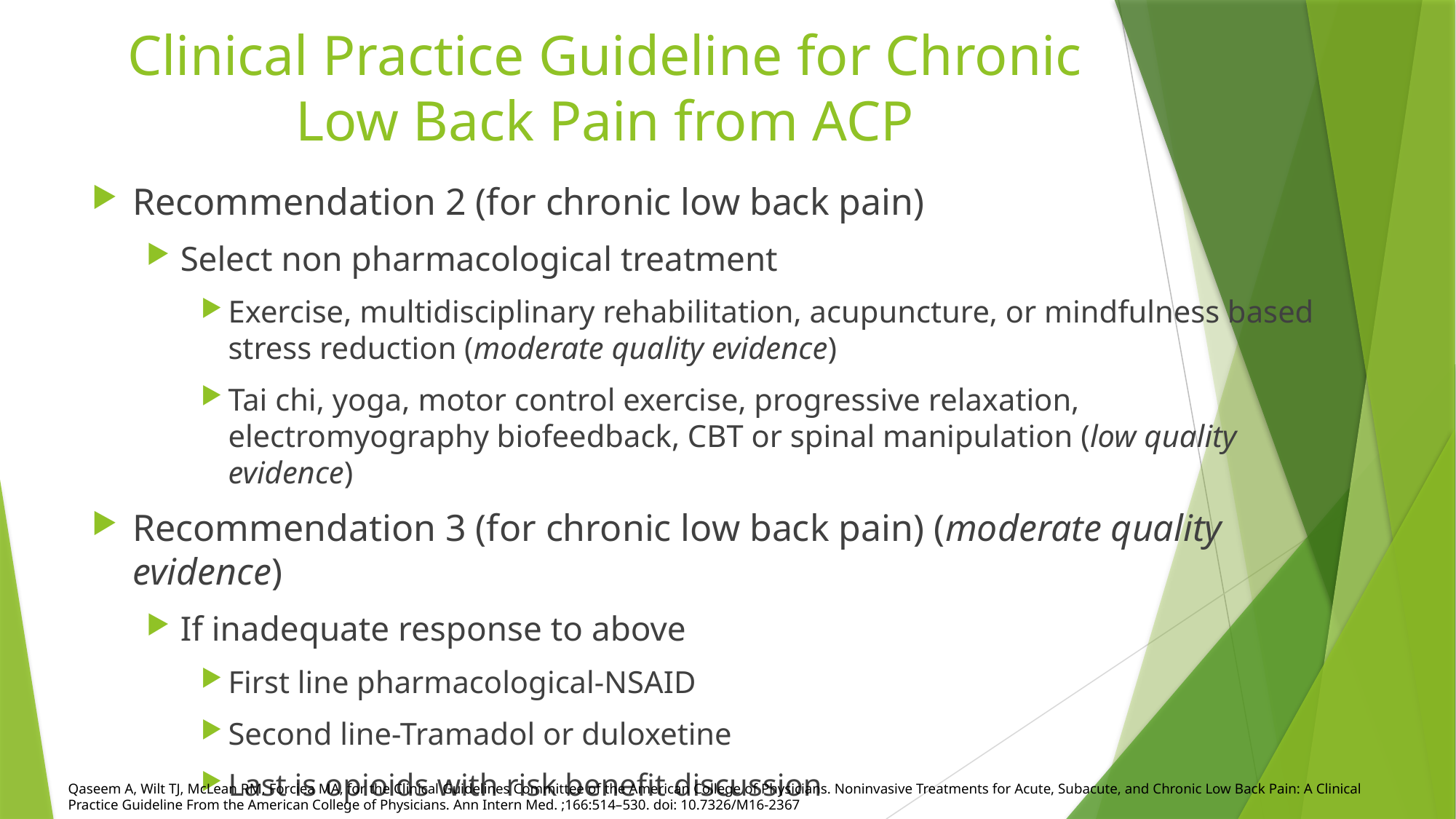

# Clinical Practice Guideline for Chronic Low Back Pain from ACP
Recommendation 2 (for chronic low back pain)
Select non pharmacological treatment
Exercise, multidisciplinary rehabilitation, acupuncture, or mindfulness based stress reduction (moderate quality evidence)
Tai chi, yoga, motor control exercise, progressive relaxation, electromyography biofeedback, CBT or spinal manipulation (low quality evidence)
Recommendation 3 (for chronic low back pain) (moderate quality evidence)
If inadequate response to above
First line pharmacological-NSAID
Second line-Tramadol or duloxetine
Last is opioids with risk benefit discussion
Qaseem A, Wilt TJ, McLean RM, Forciea MA, for the Clinical Guidelines Committee of the American College of Physicians. Noninvasive Treatments for Acute, Subacute, and Chronic Low Back Pain: A Clinical Practice Guideline From the American College of Physicians. Ann Intern Med. ;166:514–530. doi: 10.7326/M16-2367

## Slide 36
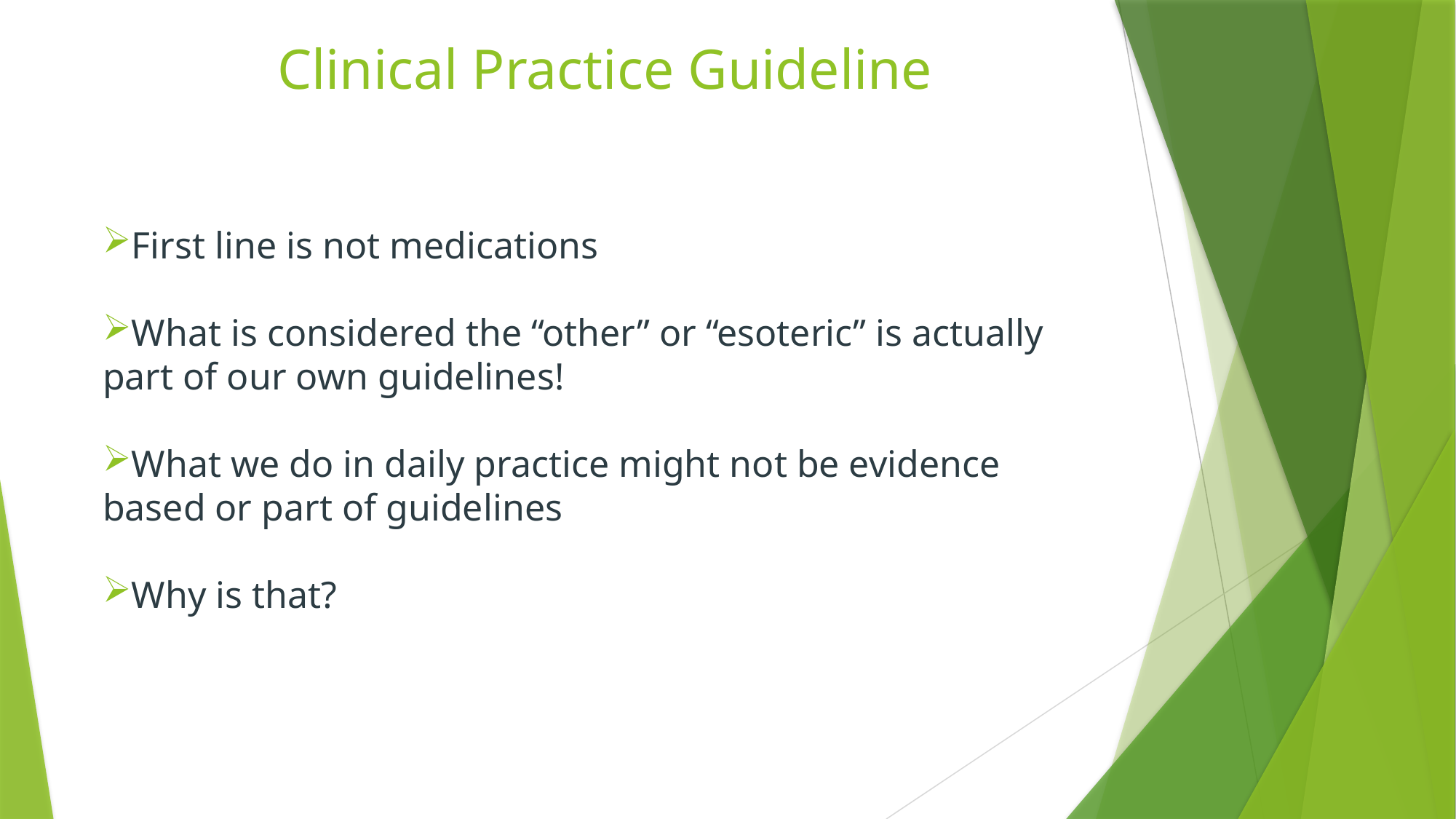

Clinical Practice Guideline
First line is not medications
What is considered the “other” or “esoteric” is actually part of our own guidelines!
What we do in daily practice might not be evidence based or part of guidelines
Why is that?

## Slide 37
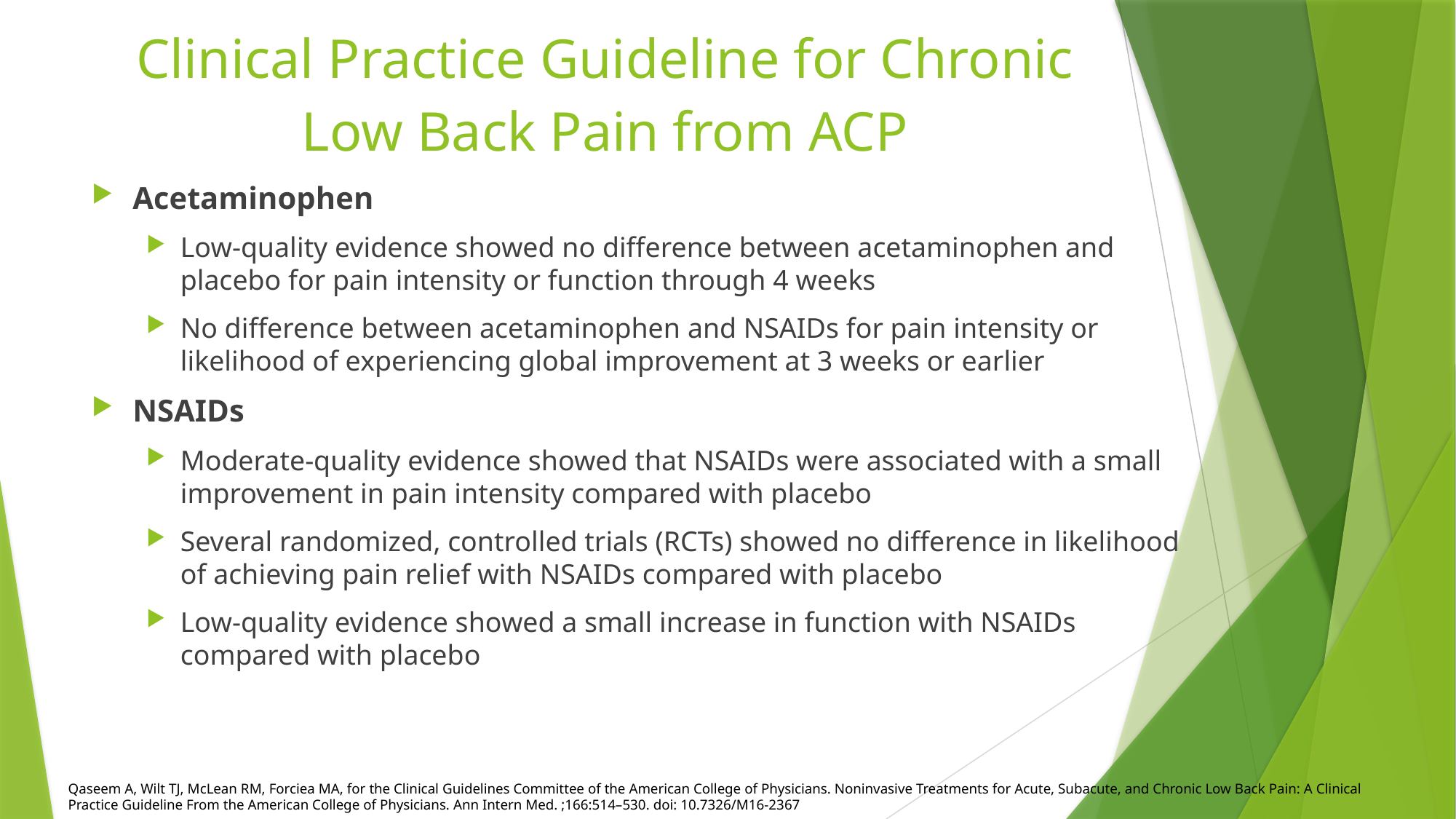

# Clinical Practice Guideline for Chronic Low Back Pain from ACP
Acetaminophen
Low-quality evidence showed no difference between acetaminophen and placebo for pain intensity or function through 4 weeks
No difference between acetaminophen and NSAIDs for pain intensity or likelihood of experiencing global improvement at 3 weeks or earlier
NSAIDs
Moderate-quality evidence showed that NSAIDs were associated with a small improvement in pain intensity compared with placebo
Several randomized, controlled trials (RCTs) showed no difference in likelihood of achieving pain relief with NSAIDs compared with placebo
Low-quality evidence showed a small increase in function with NSAIDs compared with placebo
Qaseem A, Wilt TJ, McLean RM, Forciea MA, for the Clinical Guidelines Committee of the American College of Physicians. Noninvasive Treatments for Acute, Subacute, and Chronic Low Back Pain: A Clinical Practice Guideline From the American College of Physicians. Ann Intern Med. ;166:514–530. doi: 10.7326/M16-2367

## Slide 38
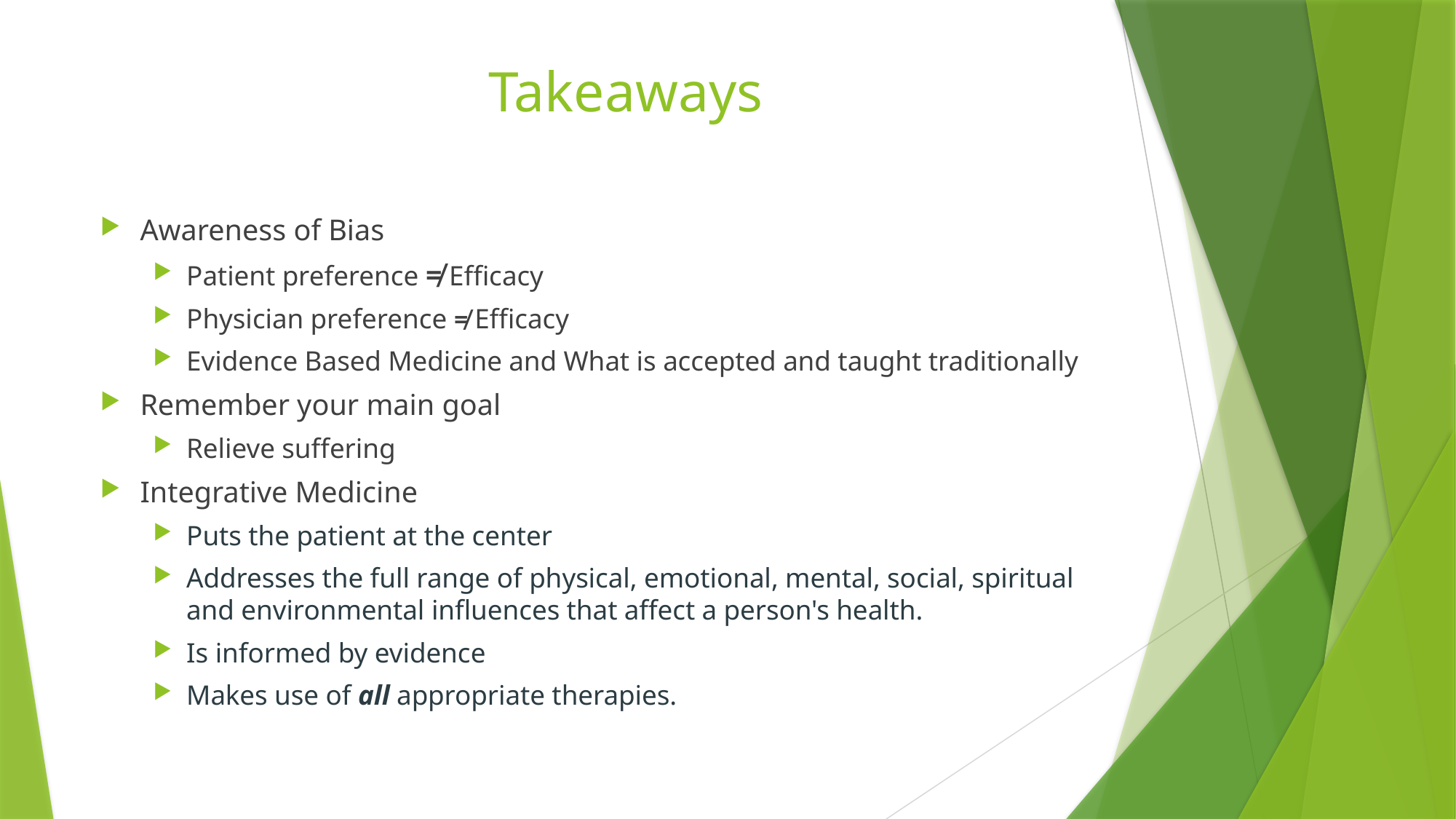

# Takeaways
Awareness of Bias
Patient preference ≠ Efficacy
Physician preference ≠ Efficacy
Evidence Based Medicine and What is accepted and taught traditionally
Remember your main goal
Relieve suffering
Integrative Medicine
Puts the patient at the center
Addresses the full range of physical, emotional, mental, social, spiritual and environmental influences that affect a person's health.
Is informed by evidence
Makes use of all appropriate therapies.

## Slide 39
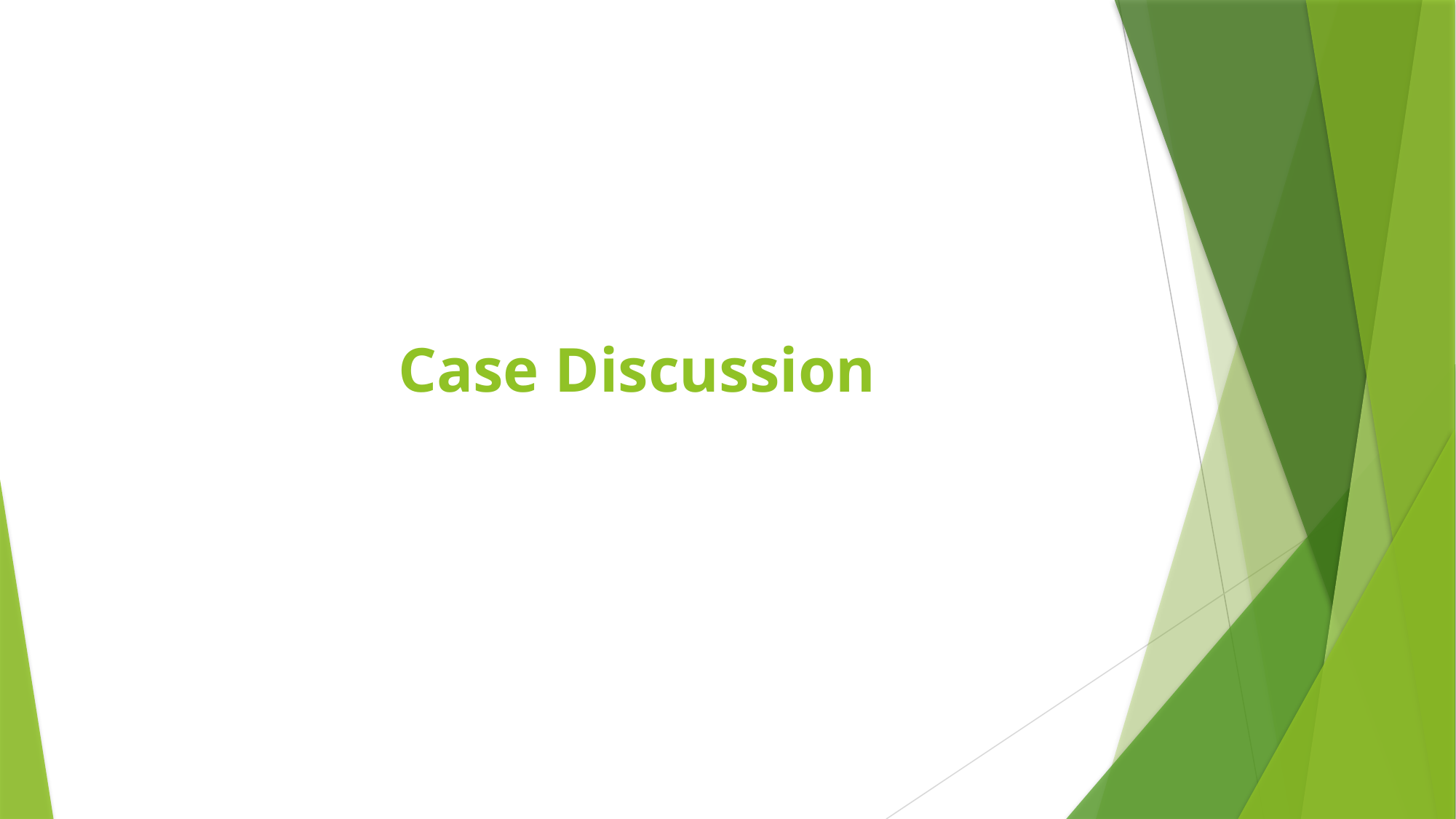

# Case Discussion

## Slide 40
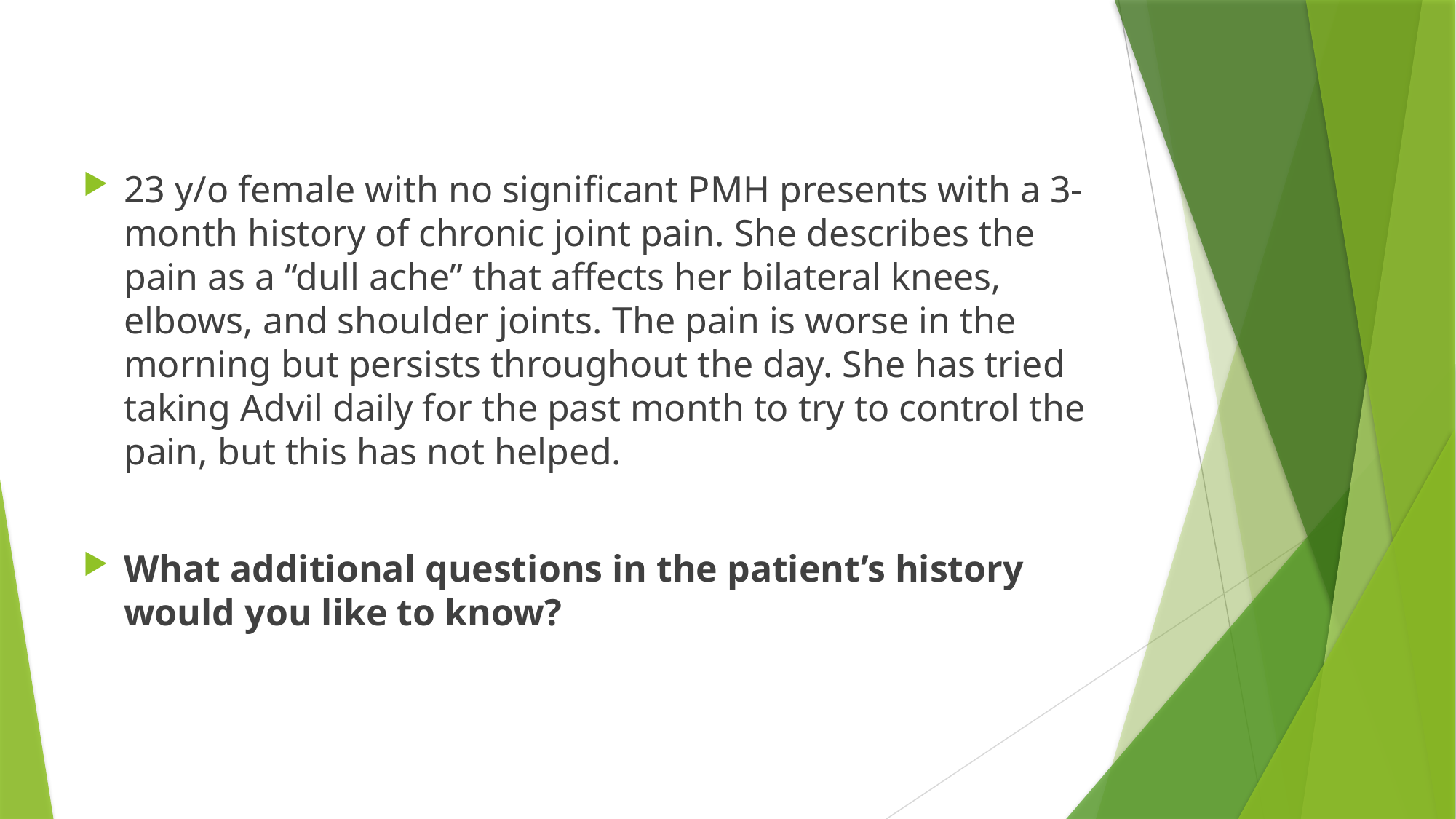

23 y/o female with no significant PMH presents with a 3-month history of chronic joint pain. She describes the pain as a “dull ache” that affects her bilateral knees, elbows, and shoulder joints. The pain is worse in the morning but persists throughout the day. She has tried taking Advil daily for the past month to try to control the pain, but this has not helped.
What additional questions in the patient’s history would you like to know?

## Slide 41
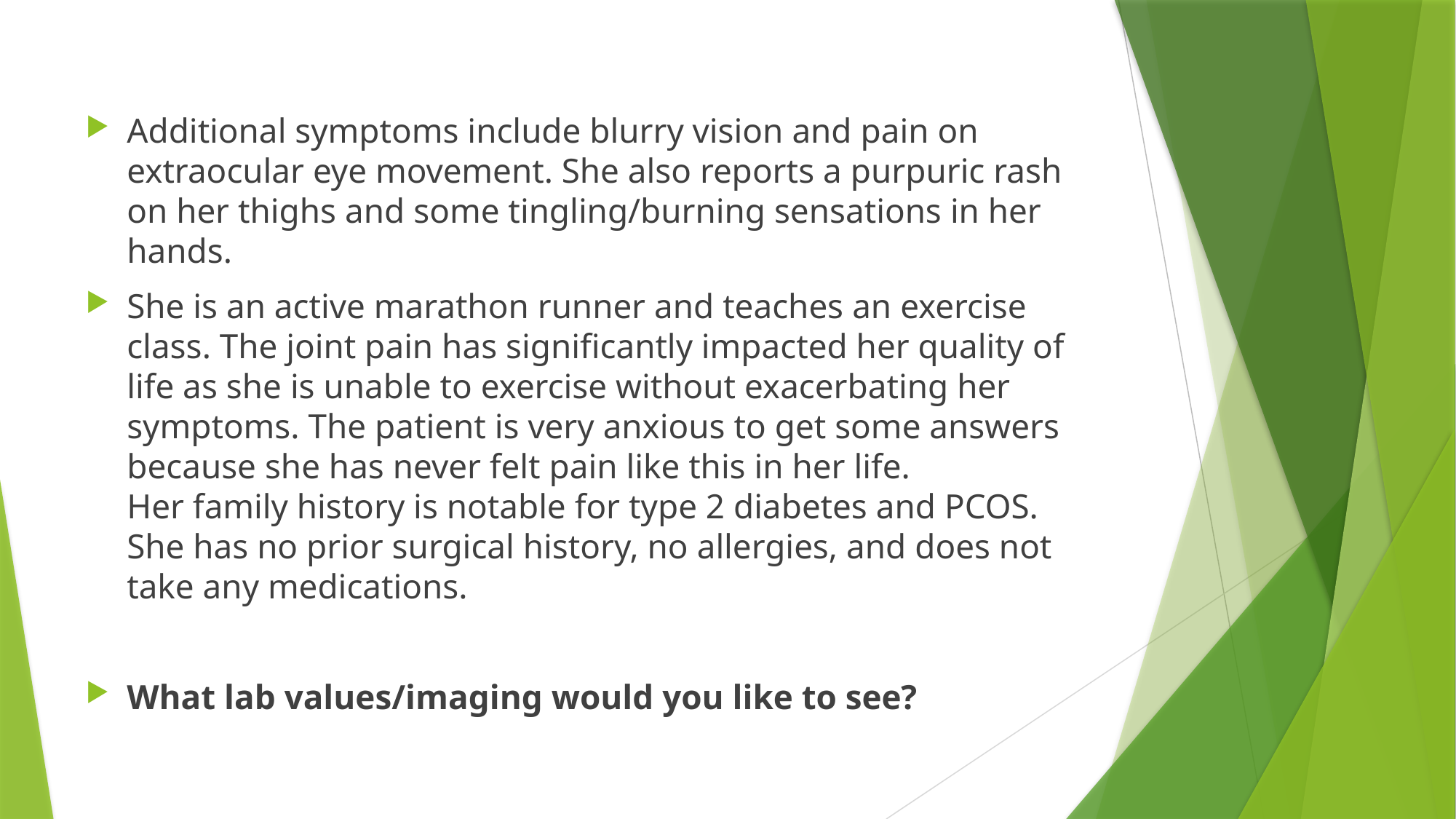

Additional symptoms include blurry vision and pain on extraocular eye movement. She also reports a purpuric rash on her thighs and some tingling/burning sensations in her hands.
She is an active marathon runner and teaches an exercise class. The joint pain has significantly impacted her quality of life as she is unable to exercise without exacerbating her symptoms. The patient is very anxious to get some answers because she has never felt pain like this in her life.
Her family history is notable for type 2 diabetes and PCOS. She has no prior surgical history, no allergies, and does not take any medications.
What lab values/imaging would you like to see?

## Slide 42
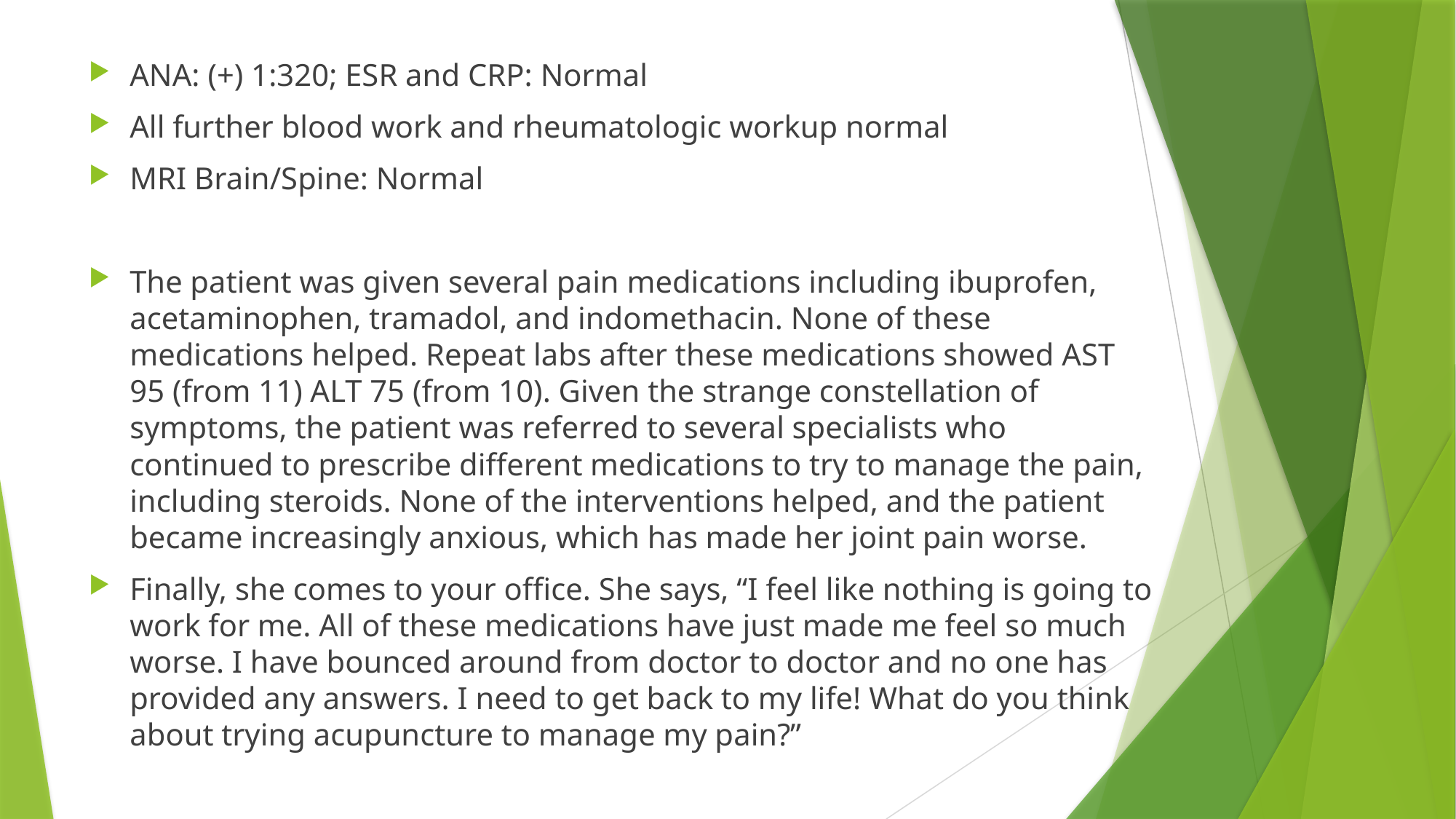

ANA: (+) 1:320; ESR and CRP: Normal
All further blood work and rheumatologic workup normal
MRI Brain/Spine: Normal
The patient was given several pain medications including ibuprofen, acetaminophen, tramadol, and indomethacin. None of these medications helped. Repeat labs after these medications showed AST 95 (from 11) ALT 75 (from 10). Given the strange constellation of symptoms, the patient was referred to several specialists who continued to prescribe different medications to try to manage the pain, including steroids. None of the interventions helped, and the patient became increasingly anxious, which has made her joint pain worse.
Finally, she comes to your office. She says, “I feel like nothing is going to work for me. All of these medications have just made me feel so much worse. I have bounced around from doctor to doctor and no one has provided any answers. I need to get back to my life! What do you think about trying acupuncture to manage my pain?”

## Slide 43
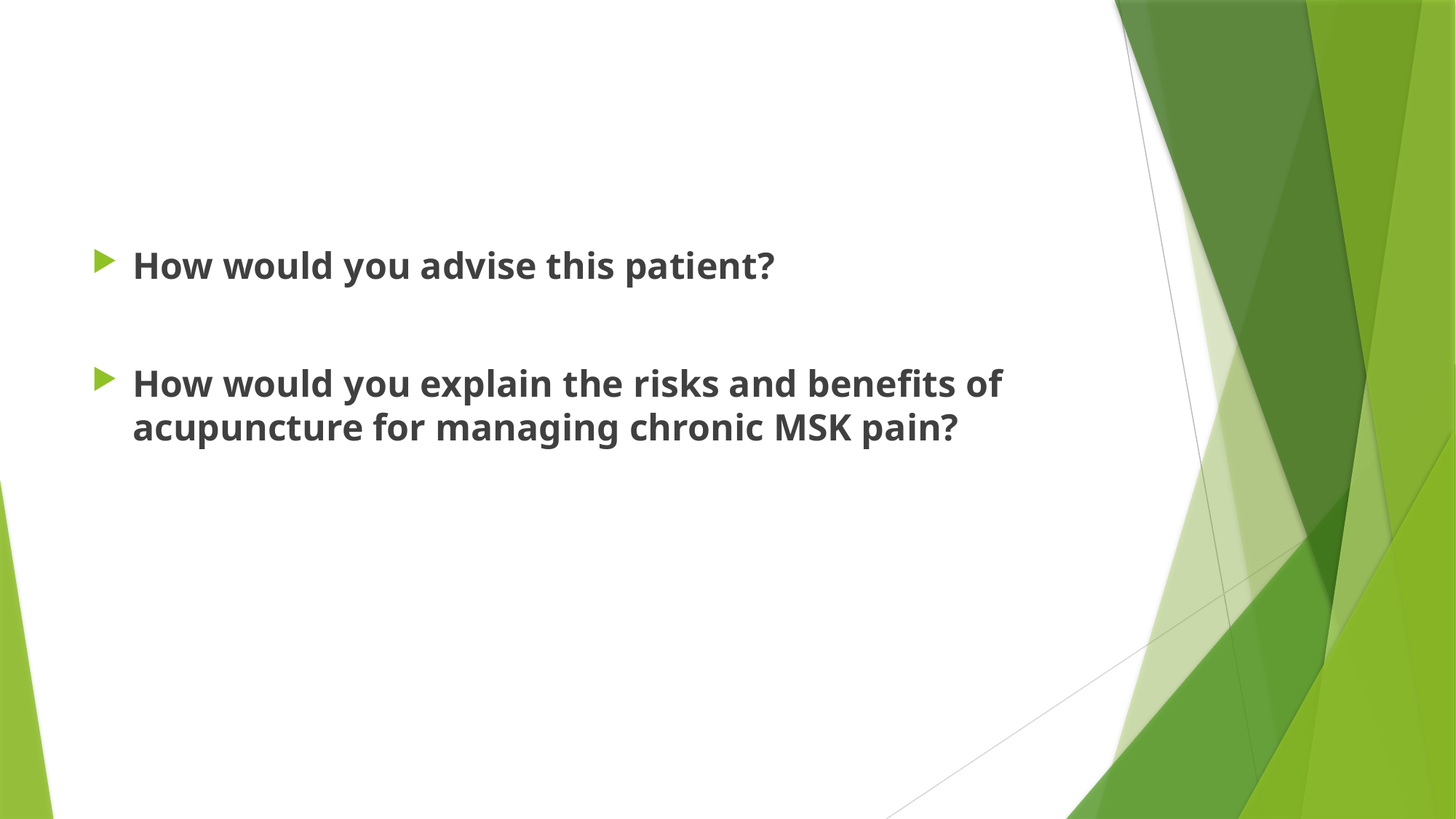

How would you advise this patient?
How would you explain the risks and benefits of acupuncture for managing chronic MSK pain?

## Slide 44
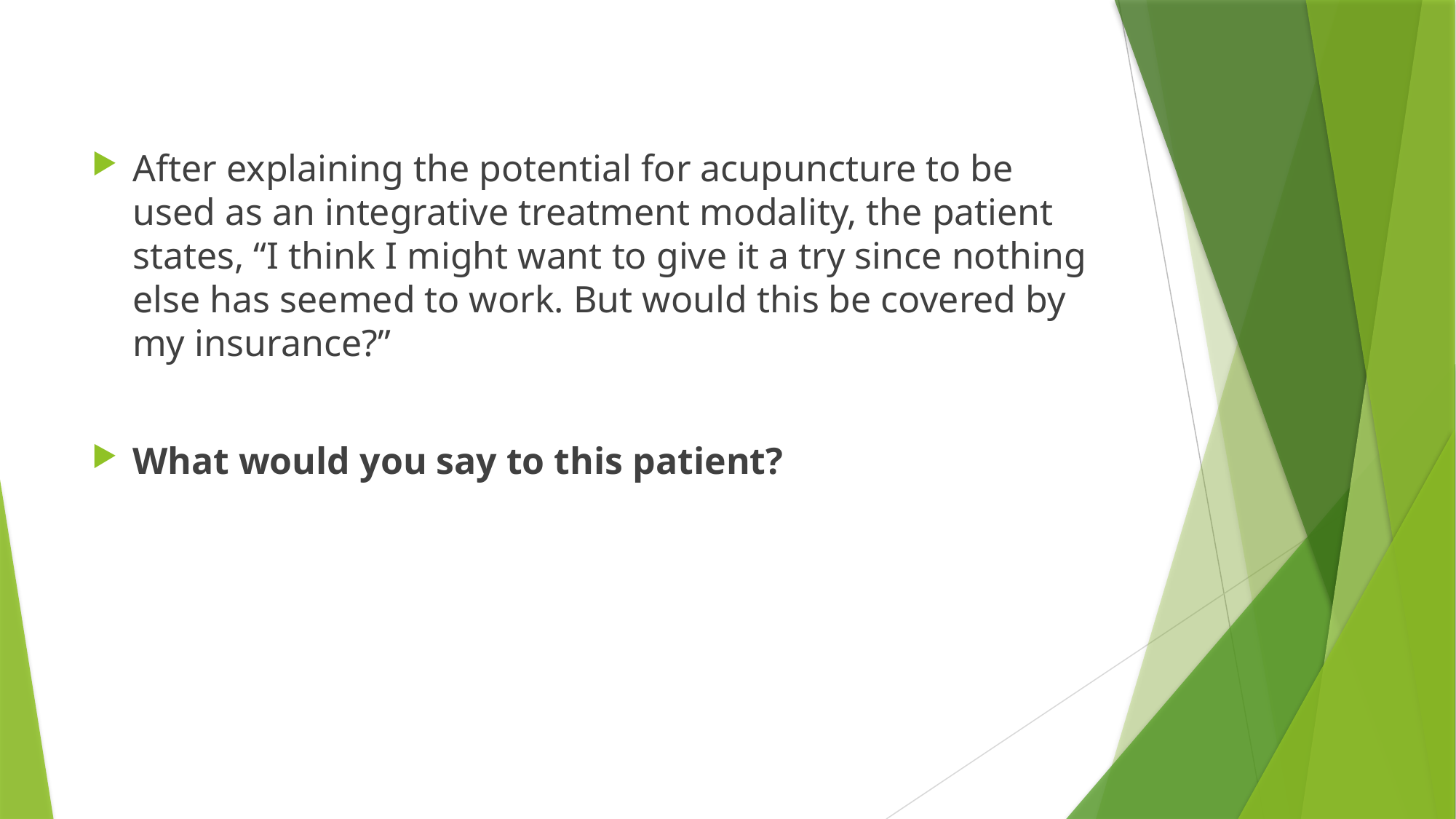

After explaining the potential for acupuncture to be used as an integrative treatment modality, the patient states, “I think I might want to give it a try since nothing else has seemed to work. But would this be covered by my insurance?”
What would you say to this patient?

## Slide 45
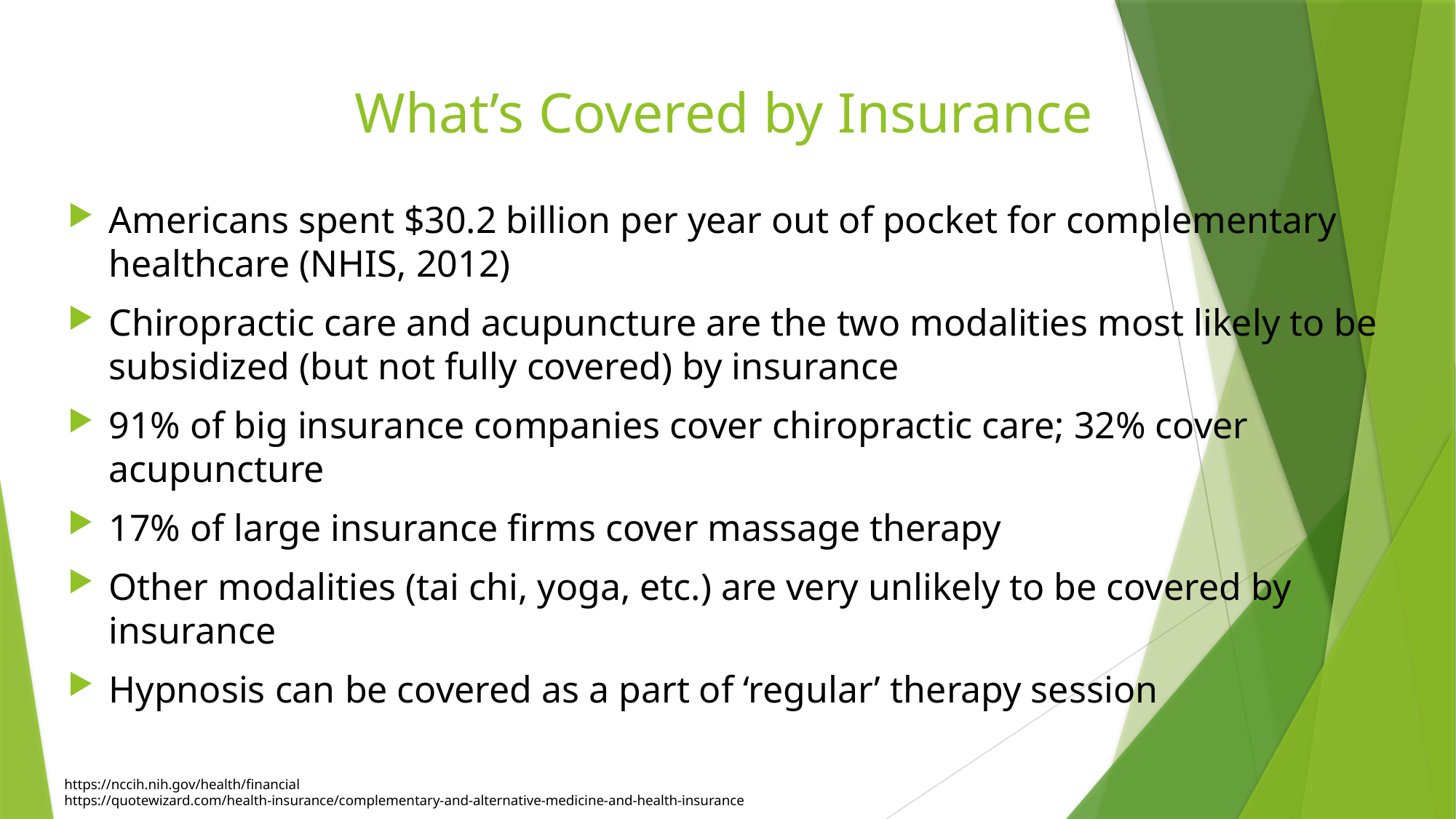

# What’s Covered by Insurance
Americans spent $30.2 billion per year out of pocket for complementary healthcare (NHIS, 2012)
Chiropractic care and acupuncture are the two modalities most likely to be subsidized (but not fully covered) by insurance
91% of big insurance companies cover chiropractic care; 32% cover acupuncture
17% of large insurance firms cover massage therapy
Other modalities (tai chi, yoga, etc.) are very unlikely to be covered by insurance
Hypnosis can be covered as a part of ‘regular’ therapy session
https://nccih.nih.gov/health/financial
https://quotewizard.com/health-insurance/complementary-and-alternative-medicine-and-health-insurance

## Slide 46
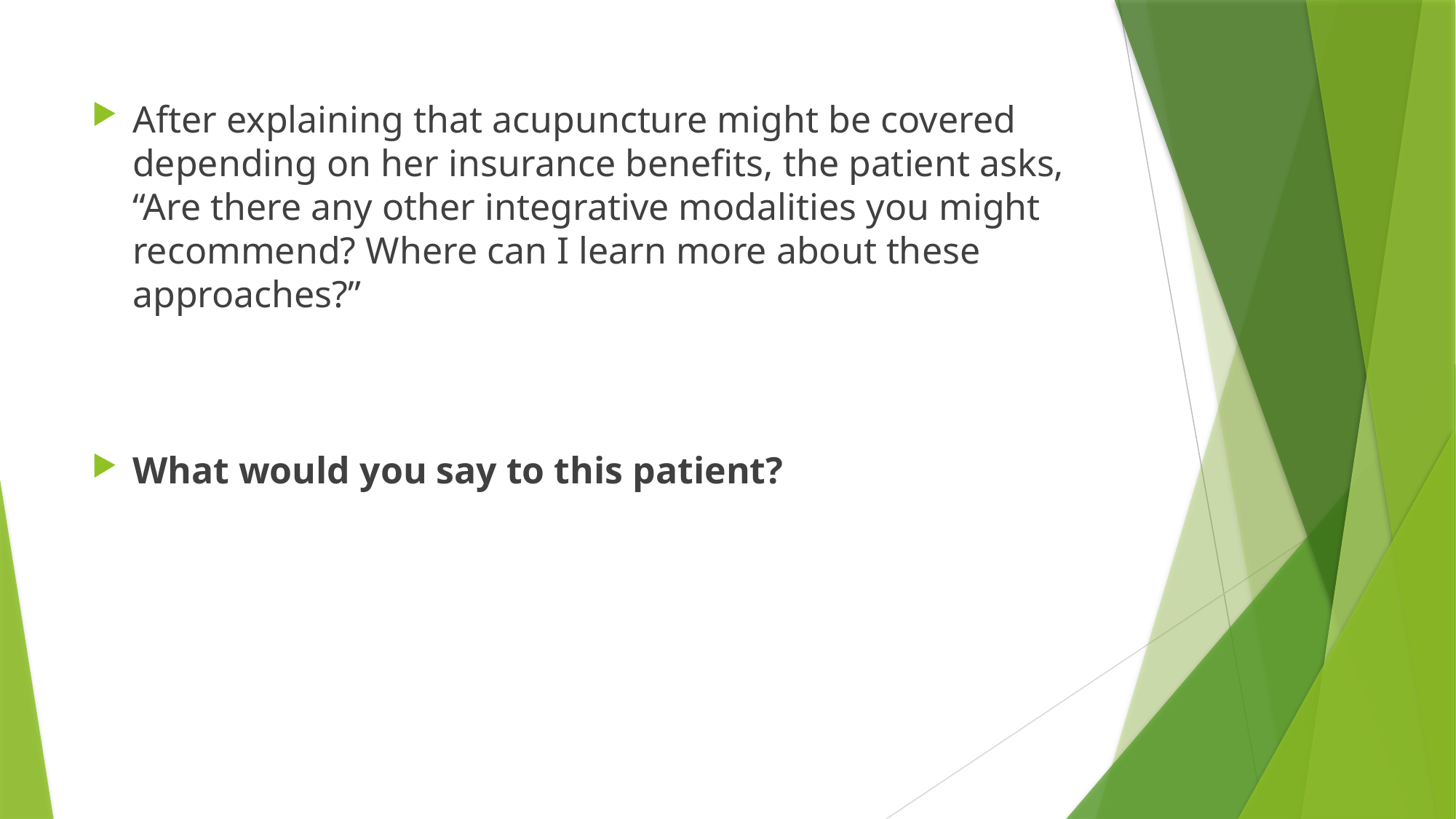

After explaining that acupuncture might be covered depending on her insurance benefits, the patient asks, “Are there any other integrative modalities you might recommend? Where can I learn more about these approaches?”
What would you say to this patient?

## Slide 47
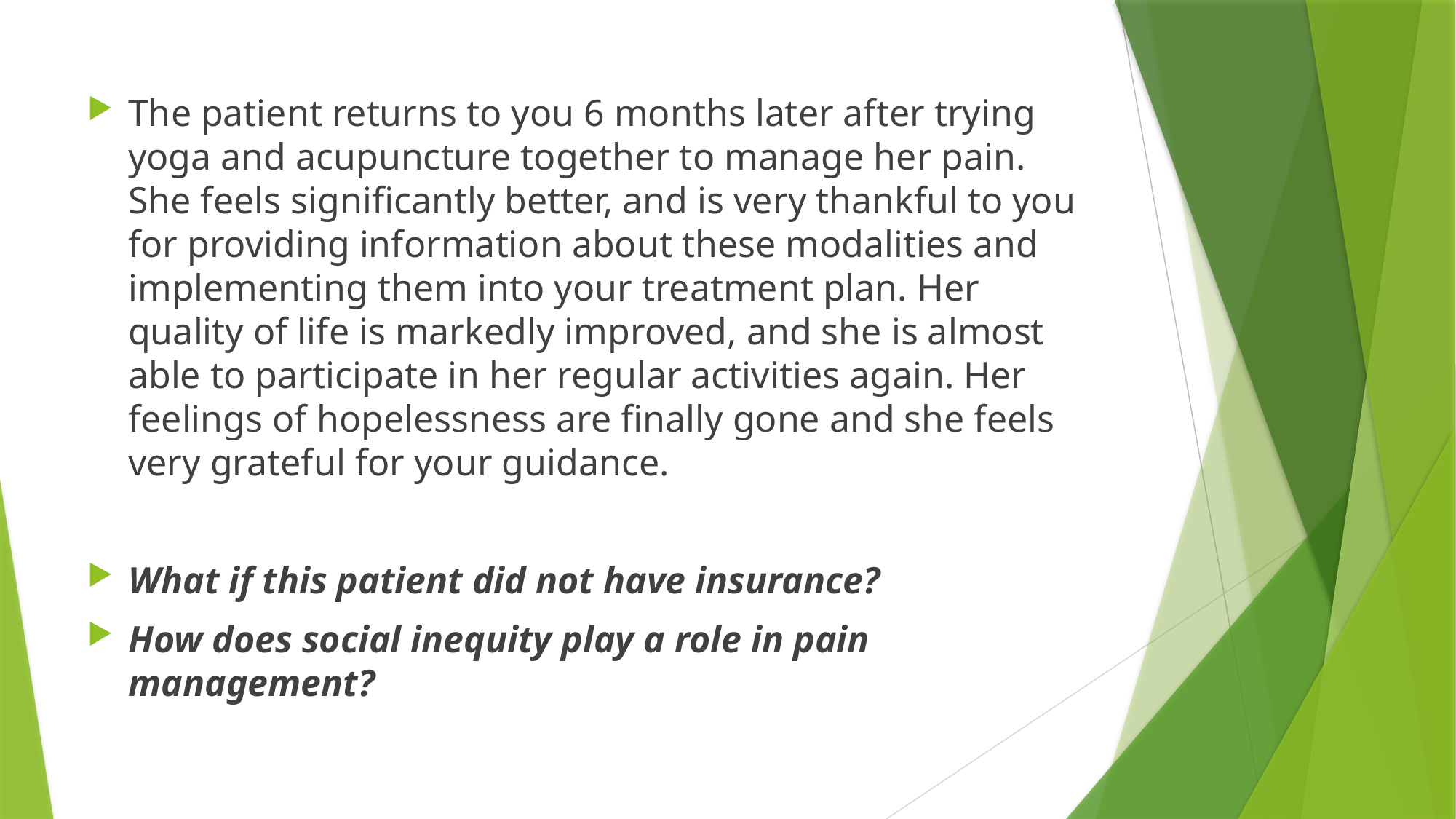

The patient returns to you 6 months later after trying yoga and acupuncture together to manage her pain. She feels significantly better, and is very thankful to you for providing information about these modalities and implementing them into your treatment plan. Her quality of life is markedly improved, and she is almost able to participate in her regular activities again. Her feelings of hopelessness are finally gone and she feels very grateful for your guidance.
What if this patient did not have insurance?
How does social inequity play a role in pain management?

## Slide 48
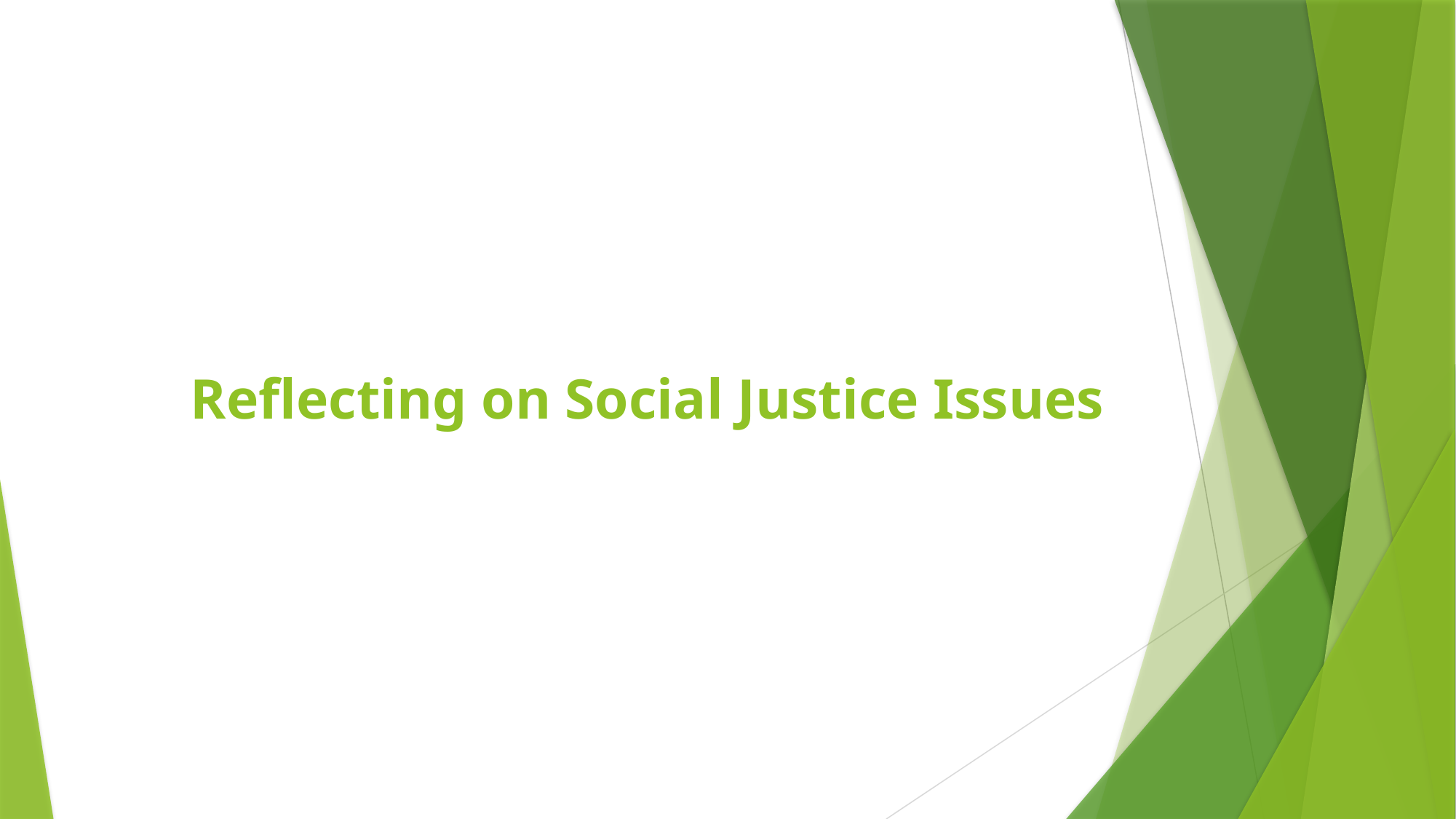

# Reflecting on Social Justice Issues

## Slide 49
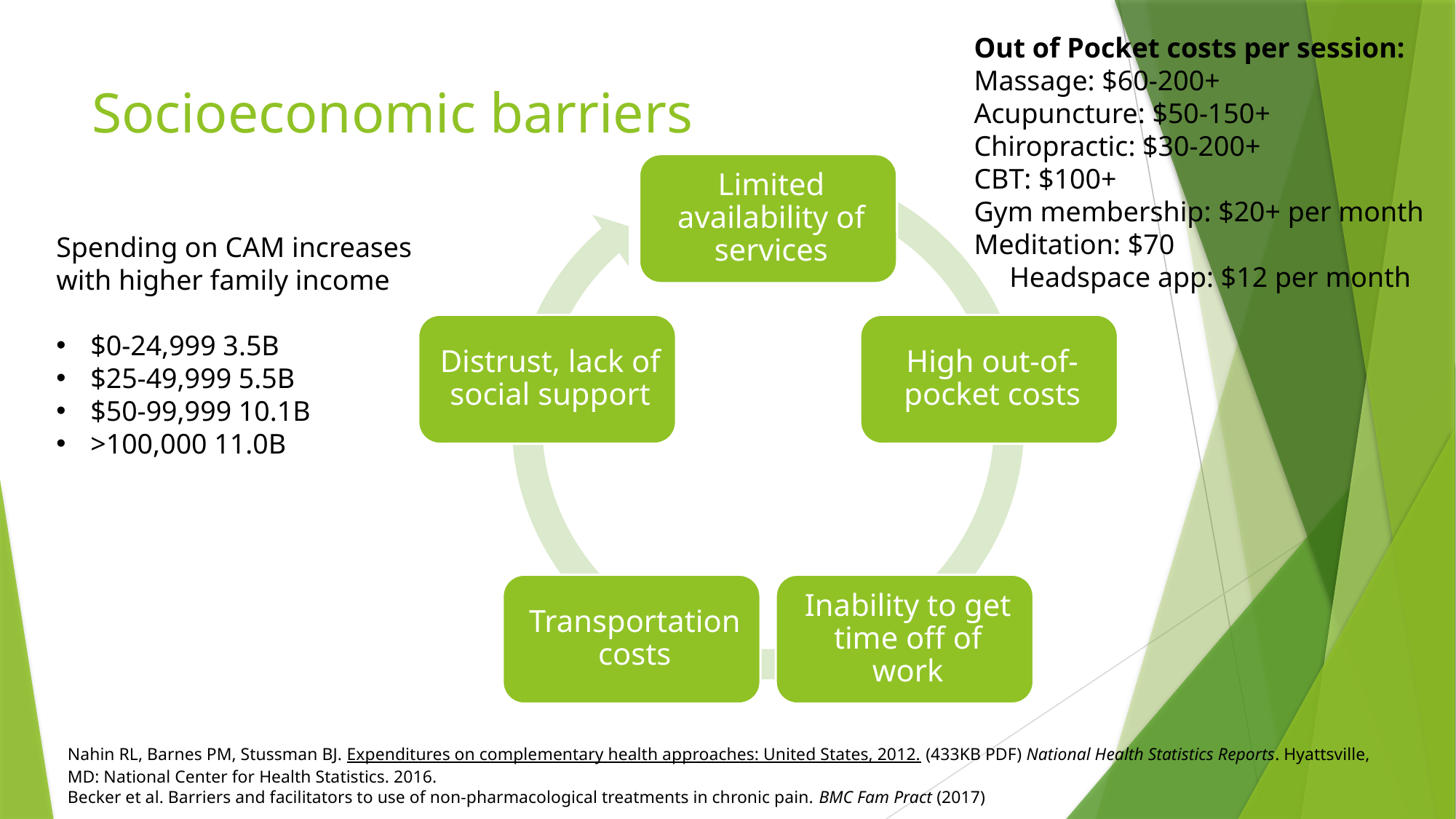

Out of Pocket costs per session:
Massage: $60-200+
Acupuncture: $50-150+
Chiropractic: $30-200+
CBT: $100+
Gym membership: $20+ per month
Meditation: $70
 Headspace app: $12 per month
# Socioeconomic barriers
Spending on CAM increases
with higher family income
$0-24,999 3.5B
$25-49,999 5.5B
$50-99,999 10.1B
>100,000 11.0B
Nahin RL, Barnes PM, Stussman BJ. Expenditures on complementary health approaches: United States, 2012. (433KB PDF) National Health Statistics Reports. Hyattsville, MD: National Center for Health Statistics. 2016.
Becker et al. Barriers and facilitators to use of non-pharmacological treatments in chronic pain. BMC Fam Pract (2017)

## Slide 50
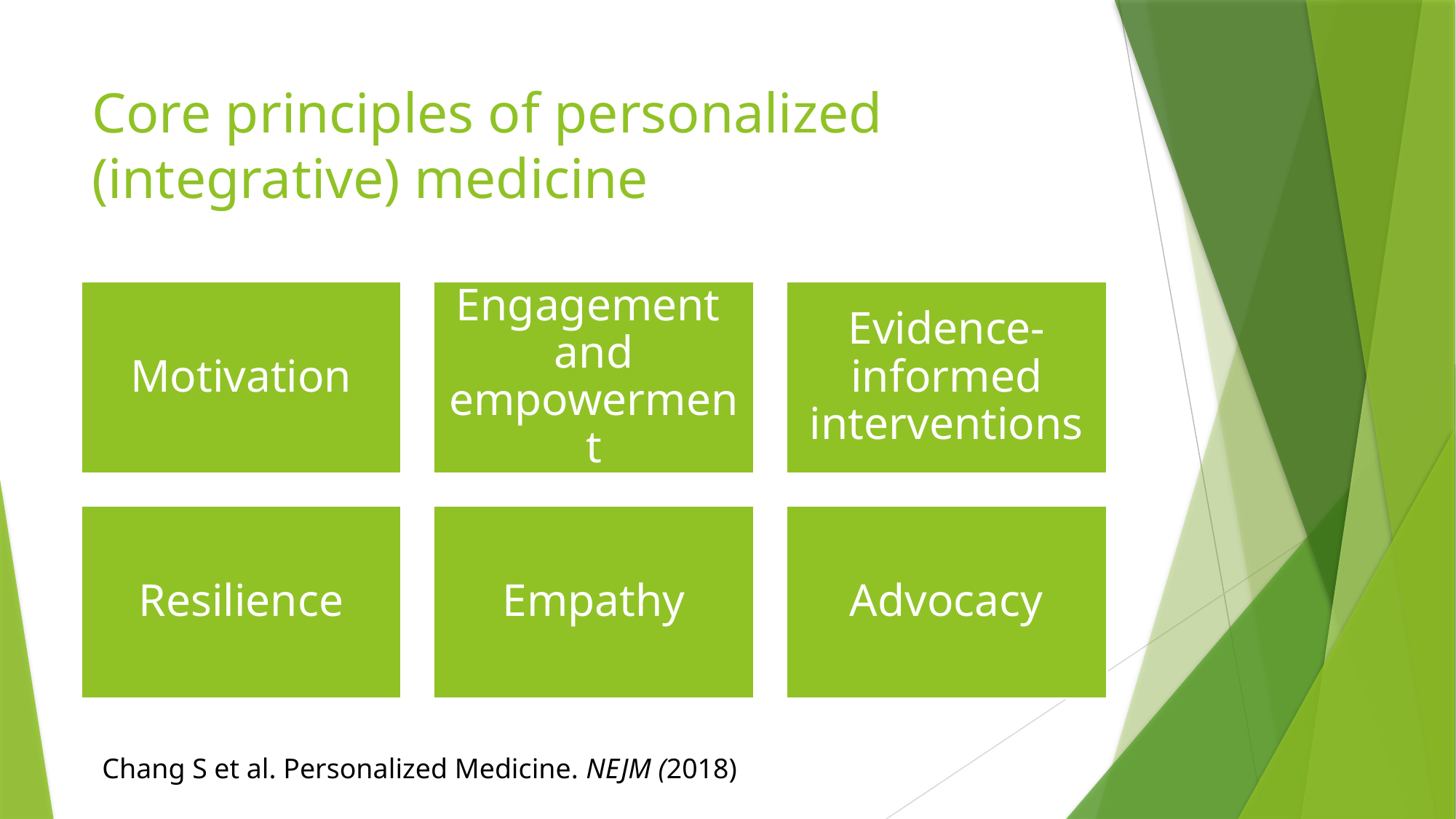

# Core principles of personalized (integrative) medicine
Chang S et al. Personalized Medicine. NEJM (2018)

## Slide 51
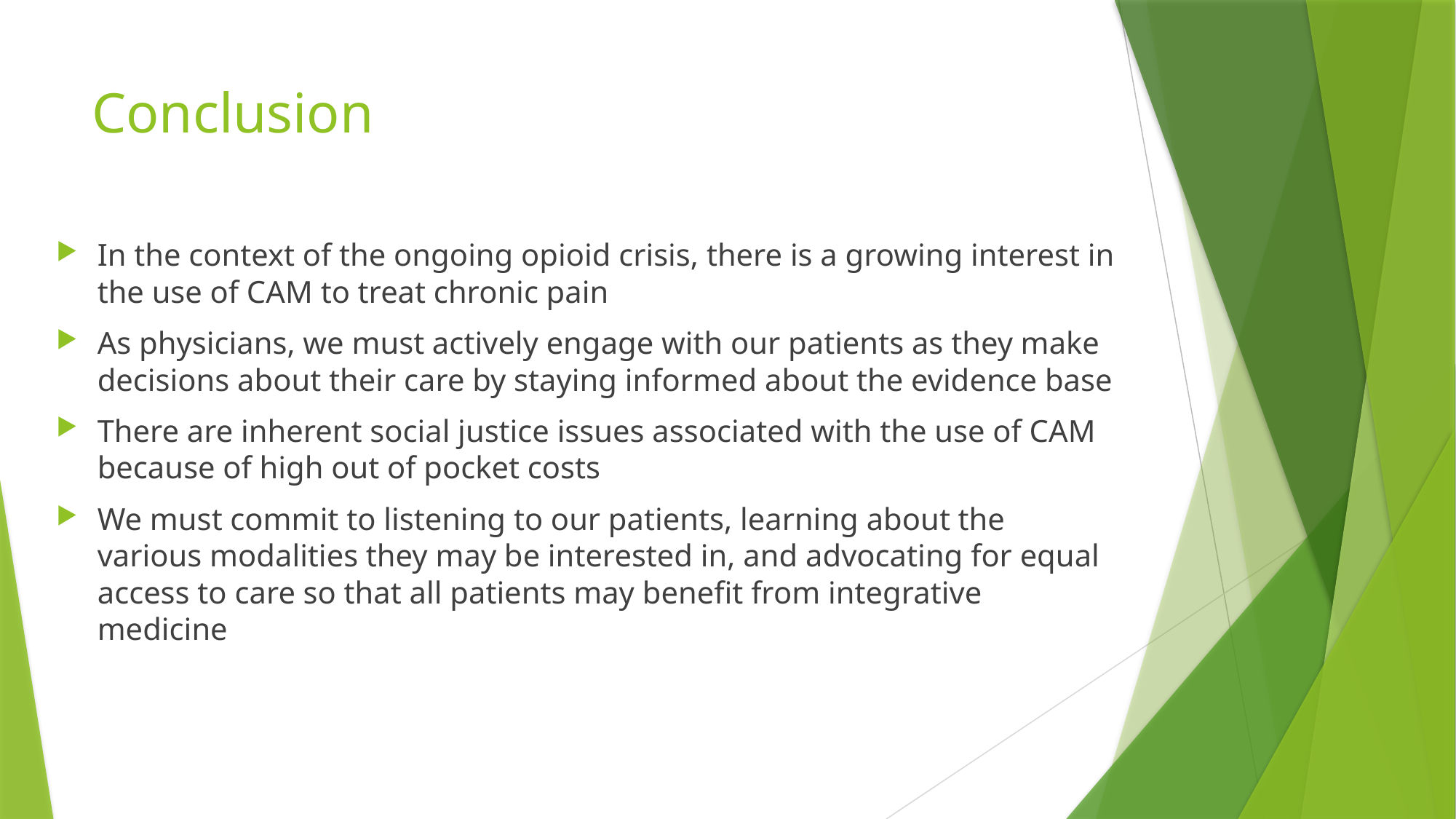

# Conclusion
In the context of the ongoing opioid crisis, there is a growing interest in the use of CAM to treat chronic pain
As physicians, we must actively engage with our patients as they make decisions about their care by staying informed about the evidence base
There are inherent social justice issues associated with the use of CAM because of high out of pocket costs
We must commit to listening to our patients, learning about the various modalities they may be interested in, and advocating for equal access to care so that all patients may benefit from integrative medicine

## Slide 52
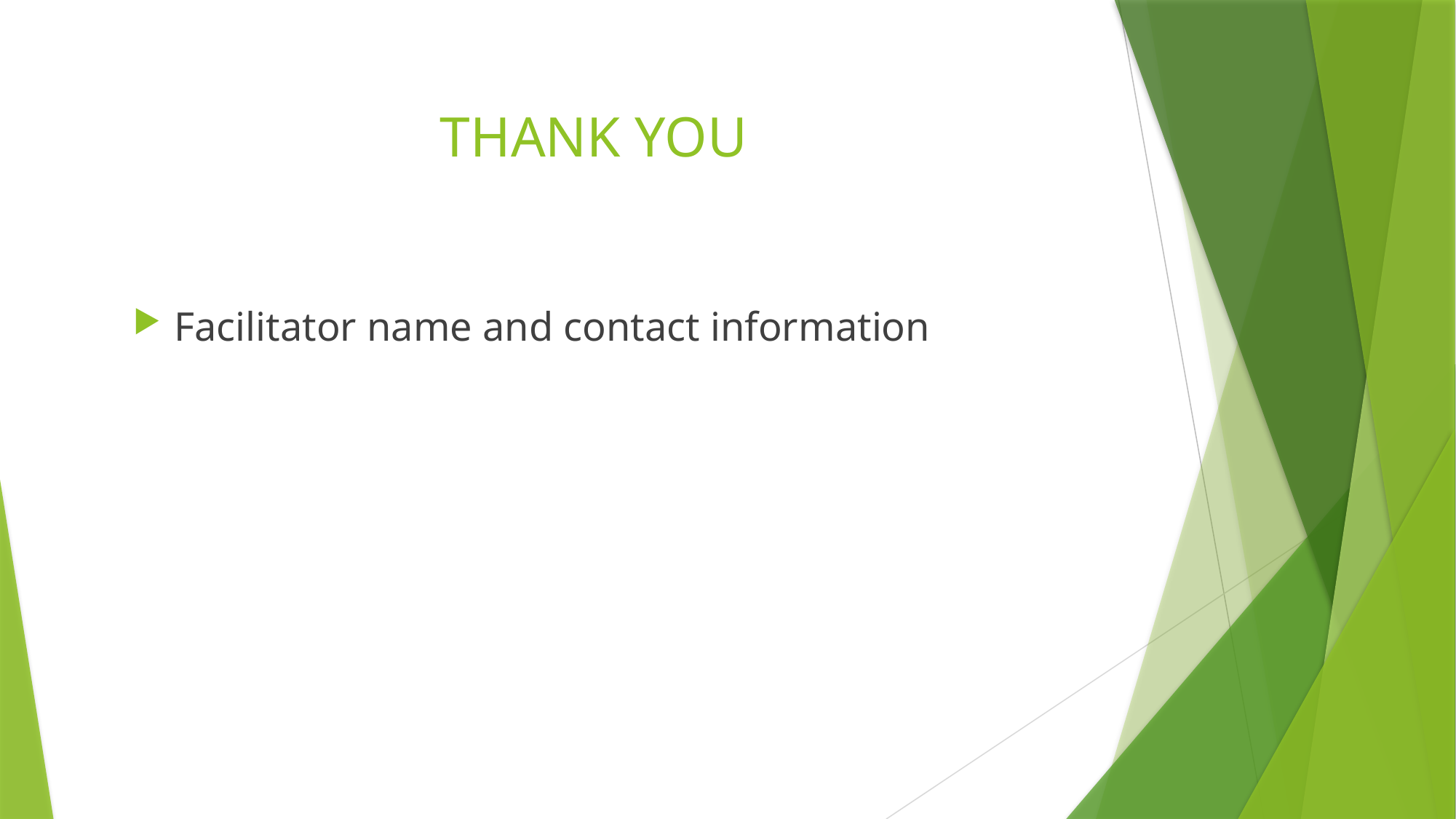

# THANK YOU
Facilitator name and contact information

## Slide 53
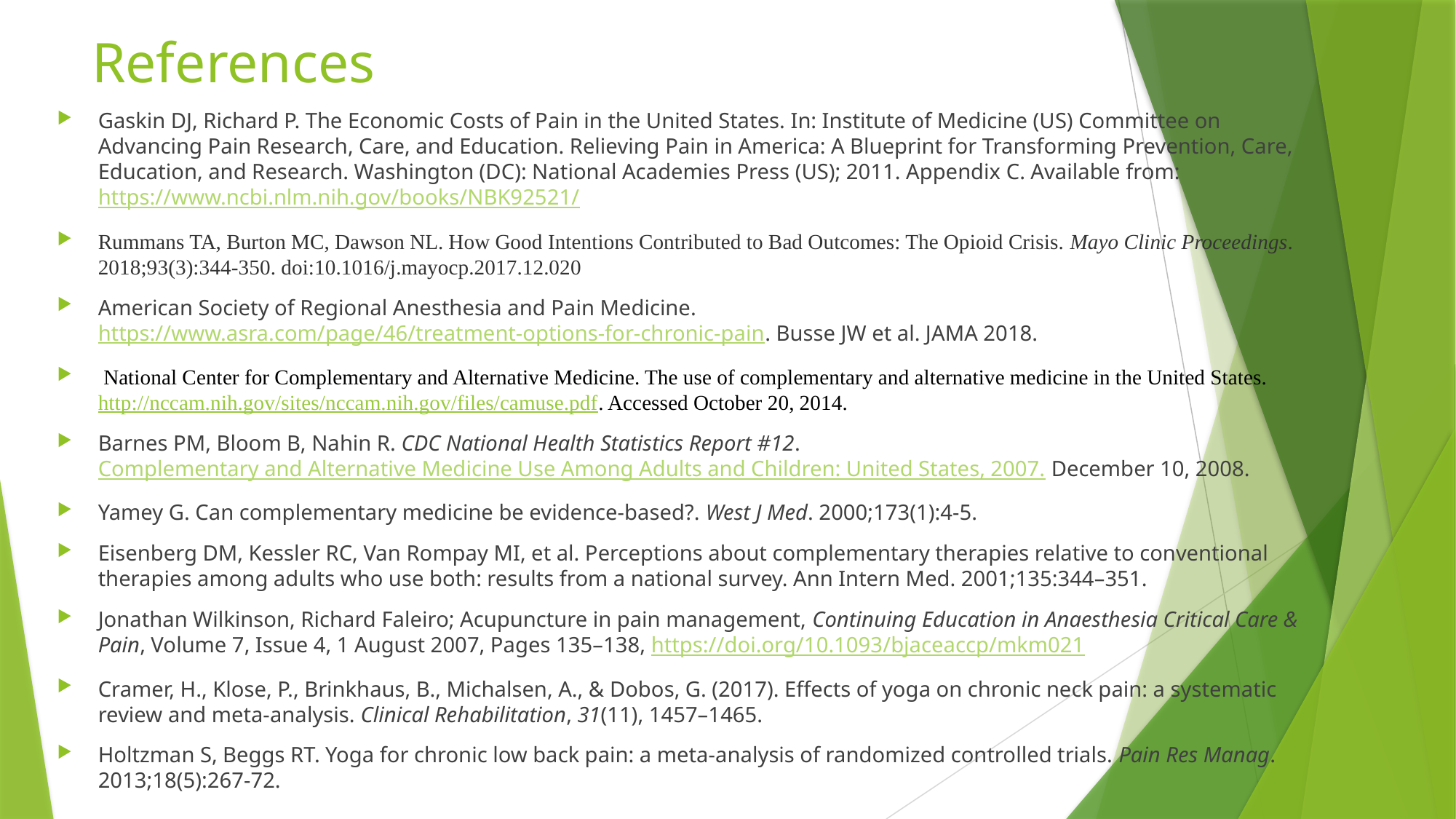

# References
Gaskin DJ, Richard P. The Economic Costs of Pain in the United States. In: Institute of Medicine (US) Committee on Advancing Pain Research, Care, and Education. Relieving Pain in America: A Blueprint for Transforming Prevention, Care, Education, and Research. Washington (DC): National Academies Press (US); 2011. Appendix C. Available from: https://www.ncbi.nlm.nih.gov/books/NBK92521/
Rummans TA, Burton MC, Dawson NL. How Good Intentions Contributed to Bad Outcomes: The Opioid Crisis. Mayo Clinic Proceedings. 2018;93(3):344-350. doi:10.1016/j.mayocp.2017.12.020
American Society of Regional Anesthesia and Pain Medicine. https://www.asra.com/page/46/treatment-options-for-chronic-pain. Busse JW et al. JAMA 2018.
 National Center for Complementary and Alternative Medicine. The use of complementary and alternative medicine in the United States. http://nccam.nih.gov/sites/nccam.nih.gov/files/camuse.pdf. Accessed October 20, 2014.
Barnes PM, Bloom B, Nahin R. CDC National Health Statistics Report #12. Complementary and Alternative Medicine Use Among Adults and Children: United States, 2007. December 10, 2008.
Yamey G. Can complementary medicine be evidence-based?. West J Med. 2000;173(1):4-5.
Eisenberg DM, Kessler RC, Van Rompay MI, et al. Perceptions about complementary therapies relative to conventional therapies among adults who use both: results from a national survey. Ann Intern Med. 2001;135:344–351.
Jonathan Wilkinson, Richard Faleiro; Acupuncture in pain management, Continuing Education in Anaesthesia Critical Care & Pain, Volume 7, Issue 4, 1 August 2007, Pages 135–138, https://doi.org/10.1093/bjaceaccp/mkm021
Cramer, H., Klose, P., Brinkhaus, B., Michalsen, A., & Dobos, G. (2017). Effects of yoga on chronic neck pain: a systematic review and meta-analysis. Clinical Rehabilitation, 31(11), 1457–1465.
Holtzman S, Beggs RT. Yoga for chronic low back pain: a meta-analysis of randomized controlled trials. Pain Res Manag. 2013;18(5):267-72.

## Slide 54
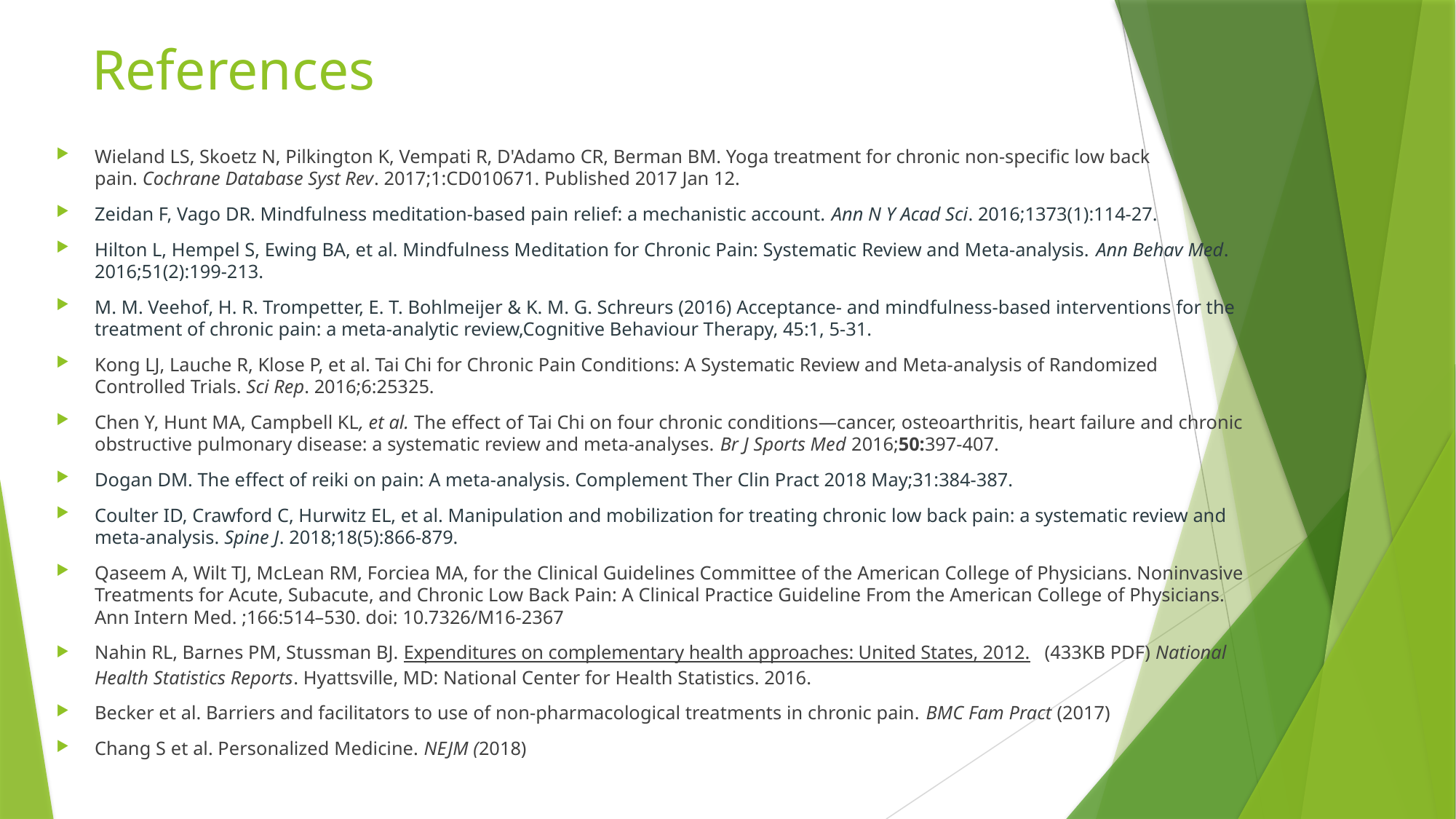

# References
Wieland LS, Skoetz N, Pilkington K, Vempati R, D'Adamo CR, Berman BM. Yoga treatment for chronic non-specific low back pain. Cochrane Database Syst Rev. 2017;1:CD010671. Published 2017 Jan 12.
Zeidan F, Vago DR. Mindfulness meditation-based pain relief: a mechanistic account. Ann N Y Acad Sci. 2016;1373(1):114-27.
Hilton L, Hempel S, Ewing BA, et al. Mindfulness Meditation for Chronic Pain: Systematic Review and Meta-analysis. Ann Behav Med. 2016;51(2):199-213.
M. M. Veehof, H. R. Trompetter, E. T. Bohlmeijer & K. M. G. Schreurs (2016) Acceptance- and mindfulness-based interventions for the treatment of chronic pain: a meta-analytic review,Cognitive Behaviour Therapy, 45:1, 5-31.
Kong LJ, Lauche R, Klose P, et al. Tai Chi for Chronic Pain Conditions: A Systematic Review and Meta-analysis of Randomized Controlled Trials. Sci Rep. 2016;6:25325.
Chen Y, Hunt MA, Campbell KL, et al. The effect of Tai Chi on four chronic conditions—cancer, osteoarthritis, heart failure and chronic obstructive pulmonary disease: a systematic review and meta-analyses. Br J Sports Med 2016;50:397-407.
Dogan DM. The effect of reiki on pain: A meta-analysis. Complement Ther Clin Pract 2018 May;31:384-387.
Coulter ID, Crawford C, Hurwitz EL, et al. Manipulation and mobilization for treating chronic low back pain: a systematic review and meta-analysis. Spine J. 2018;18(5):866-879.
Qaseem A, Wilt TJ, McLean RM, Forciea MA, for the Clinical Guidelines Committee of the American College of Physicians. Noninvasive Treatments for Acute, Subacute, and Chronic Low Back Pain: A Clinical Practice Guideline From the American College of Physicians. Ann Intern Med. ;166:514–530. doi: 10.7326/M16-2367
Nahin RL, Barnes PM, Stussman BJ. Expenditures on complementary health approaches: United States, 2012. (433KB PDF) National Health Statistics Reports. Hyattsville, MD: National Center for Health Statistics. 2016.
Becker et al. Barriers and facilitators to use of non-pharmacological treatments in chronic pain. BMC Fam Pract (2017)
Chang S et al. Personalized Medicine. NEJM (2018)
