# Supplementary material for: Implementing an Interactive Introduction to Complementary Medicine for Chronic Pain Management Into the Medical School Curriculum
Source: MedEdPORTAL. 2020 Dec 29;16:11056. doi: 10.15766/mep_2374-8265.11056 (PMC7780745; doi:10.15766/mep_2374-8265.11056)
Supplement: Supplementary file 1 — CAM Lecture.pptxStudent Perspective Script.docxFacilitator Guide.docxPresession Survey.docxPostSession Survey.docx [file mep_2374-8265.11056-s001.zip › B. Student Perspective Script.docx]

**Patient Perspective Script**

I just wanted to share a little bit of my personal story. Last year I ran into some unexpected health problems. I started experiencing a slew of strange symptoms, and one of those symptoms was pretty bad joint and MSK pain. I ended up being referred to over 15 different doctors and was put on 12 different types of medications over a three-month time period. While doctors were trying to figure out the underlying problem, my pain symptoms persisted, and I found myself struggling.

I was struggling to deal with my new pain. As someone who was always physically active, it was hard to deal with the fact that I couldn’t exercise the way I was used to. It was hard to accept my new identity as a patient, while going to school to learn how to be a physician.

I was struggling with the anxiety of having an unknown illness. Having 15 different doctors poke and prod and come up with their own theories that were inconsistent with each other was mentally taxing.

I was struggling to keep up as a student. Running around to doctor’s appointments during the middle of medical school tests was exhausting and stressful.

Ultimately, it was these relapsing patterns of stress that made my symptoms worse. On a quest to figure out what was going on with me and determined to find the “magic pill” that would take everything away, I lost sight of the importance of taking care of myself and was not reminded to do so by most of my physicians.

As I worked desperately to finish up second year, my emotions were also filled with an overwhelming amount of gratitude: I felt incredibly thankful to have access to great care, thankful to have the resources to physically reach this care, thankful that whatever was happening to me was not life threatening, thankful to have an amazing support system in my friends and family. But despite all of these things going for me, I ultimately felt hopeless. My symptoms persisted and the side effects of medications were doing more harm than good.

After finally finishing second year, I was at my lowest point. I realized I didn’t have a choice but to take some time off school to focus on my health. It was at this point that I was referred to the Mayo clinic in Minnesota—and it was there that I received some sort of closure about what was going on with me. But more importantly, it was there that I was first presented information that made me feel like I had some control over my situation. I realized that this was actually just as important to me as finding a diagnosis—having some power and autonomy to take care of myself gave me strength. Doctors showed me studies about the evidence behind modalities like yoga and meditation for dealing with chronic pain. I had always practiced yoga, but it was the first time it was presented to me as a therapeutic strategy. I was stunned that it had taken me over 6 months of doctor’s visits before someone even mentioned these types of strategies, and even more stunned by the amount of data available.

I came back from Minnesota with a new resolve—I had tried over a dozen different medications that did nothing to improve my quality of life, but this was one thing I had not tried. I integrated targeted yoga and meditation therapies into my life. I used acupuncture to help manage my pain. I read about the evidence behind anti-inflammatory diets to reduce pain. I made a conscious effort to change my daily routine, my diet, my life. And I noticed a difference—my pain levels began to reduce. I felt my energy levels increasing, and my ability to call on my resilience stores increasing. I started to feel well enough to take STEP and come back to school and am now going through rotations. If you had told me this is where I’d be at this time last year, I wouldn’t have believed you.

As a future physician, whether you go into surgery or psychiatry, your day in and day out will be about coming up with solutions for your patients. Learning about the evidence base behind different practices that your patients may be interested in trying is one way to offer support to your patients who may be struggling. It is my hope that we go on to be the kinds of physicians that are not only well trained to help save people’s lives, but who make a conscious effort to help people truly live.
