# Supplementary material for: Implementing an Interactive Introduction to Complementary Medicine for Chronic Pain Management Into the Medical School Curriculum
Source: MedEdPORTAL. 2020 Dec 29;16:11056. doi: 10.15766/mep_2374-8265.11056 (PMC7780745; doi:10.15766/mep_2374-8265.11056)
Supplement: Supplementary file 1 — CAM Lecture.pptxStudent Perspective Script.docxFacilitator Guide.docxPresession Survey.docxPostSession Survey.docx [file mep_2374-8265.11056-s001.zip › C. Facilitator Guide.docx]

**Complementary and Alternative Medicine Small Group Facilitator Guide**

- Define and describe the CAM modality
- What is the origin and history of the CAM modality?
- How is the CAM modality practiced today?
- (Optional) NCCIH Video on CAM Therapies: <https://www.youtube.com/watch?time_continue=2&v=oUCEyZYBf_o&feature=emb_logo>
- (For CAM facilitators with expertise in modality) discuss real life experiences involving the improvement of chronic pain while practicing the CAM modality. This can be personal experiences or experiences of clients/patients.
- Discuss contraindications for each CAM modality in regards for chronic pain
  - Yoga: risks include musculoskeletal injuries, nerve or artery injuries. Patients with glaucoma, cervical disk disease, and osteoporosis should be cautious while performing inversions and extreme spinal flexion/extension.
  - Acupuncture: absolute contraindications include use in patients with severe neutropenia after myelosuppressive chemotherapy, insertion at site of active infection or malignancy, in patients with an automatic implantable cardioverter-defibrillator (AICD) or pacemaker.
  - Meditation: there are no contraindications to meditation.
  - Tai Chi: generally very safe; one systematic review noted that it may be associated with increased aches/pains but is very unlikely to cause injury.
  - Reiki: there are no known risks for receiving reiki treatment.
  - Spinal Manipulation: risks include disk herniation, cauda equina syndrome, vertebrobasilar occlusion or dissection, and carotid dissection. Patients with risk factors for dissection (prior history of dissection, neck trauma, TIA, Ehlers-Danlos Syndrome) should avoid spinal manipulation. In addition, caution should be taken in patients with bleeding disorders, inflammatory spondyloarthropathy, osteoporosis, Down syndrome, upper cervical instability, and chronic anticoagulation.
- Active introduction of CAM modality. This is a 40-50-minute live practice in which the facilitator teaches and performs the basics of the CAM modality while students follow instructions and attempt the practice.
  - For CAM facilitators who are not experts in the CAM modality or not able to teach, find below instructional videos which are at an introductory level
    - Yoga: <https://www.youtube.com/watch?v=BFaXfQpxnps>
    - Tai Chi: <https://www.youtube.com/watch?v=B0QDRqHNNE8>
    - Meditation: <https://www.youtube.com/watch?v=vhTcG2POu20>
- Emphasize that humanism should be at the center of patient care, particularly in terms of discussing CAM options with different patient populations who may or may not have access to the same levels of care. The principles of “advocacy” and “engagement and empowerment” are essential to providing personalized (and integrative) medicine.
- Take any questions and open the discussion for any medical students who may have personal experiences and thoughts.

*** Note that each CAM modality will vary greatly due to specific poses, exercises, and sequences taught in each session. The goal of the sessions is to expose students to the basics of the CAM modality, its relationship to alleviating chronic pain, and to allow students to physically experience the modality in order to be better informed when suggesting these practices to future patients.

Sources:

1. NCCIH. CAM Therapies: Meditation, Yoga and Cognitive Behavioral Therapies [Video]. YouTube. <https://www.youtube.com/watch?time_continue=2&v=oUCEyZYBf_o&feature=emb_logo> Published August 26, 2013. Accessed July 28, 2020.
2. Fishbein, D., Saper R., Aronson, M., Kunins, L. Overview of yoga. In: Post TW, ed. UpToDate. Waltham, MA: UpToDate Inc. <https://www.uptodate.com/contents/overview-of-yoga#references>. Accessed August 24, 2020.
3. Ahn, A., Aronson, M., Kunins, L. Acupuncture. In: Post TW, ed. UpToDate. Waltham, MA: UpToDate Inc. <https://www.uptodate.com/contents/acupuncture?search=acupuncture&source=search_result&selectedTitle=1~150&usage_type=default&display_rank=1#H15>. Accessed August 24, 2020.
4. Goyal M, Singh S, Sibinga EM, et al. Meditation programs for psychological stress and well-being: a systematic review and meta-analysis. *JAMA Intern Med*. 2014;174(3):357-368. doi:10.1001/jamainternmed.2013.13018
5. Wayne PM, Berkowitz DL, Litrownik DE, et al. What do we really know about the safety of tai chi? A systematic review of adverse event reports in randomized trials. *Archives of Physical Medicine and Rehabilitation*. 2014;95(12):2470–2483.
6. Demir Doğan M. The effect of reiki on pain: A meta-analysis. *Complement Ther Clin Pract*. 2018;31:384-387. doi:10.1016/j.ctcp.2018.02.020
7. Shekelle, P., Tang, B., Atlas, S., Kunins, L. Spinal manipulation in the treatment of musculoskeletal pain. In: Post TW, ed. UpToDate. Waltham, MA: UpToDate Inc. <https://www.uptodate.com/contents/spinal-manipulation-in-the-treatment-of-musculoskeletal-pain?search=spinal%20manipulation&source=search_result&selectedTitle=1~46&usage_type=default&display_rank=1#H11>. Accessed August 24, 2020.
8. Adriene Mishler. Yoga for Chronic Pain | Yoga with Adriene [Video]. YouTube. <https://www.youtube.com/watch?v=BFaXfQpxnps> Published November 2, 2019. Accessed July 28, 2020.
9. Chris Pei. Tai Chi for Beginners – Best instructional video: Lesson 1 Tai Chi [Video]. YouTube. <https://www.youtube.com/watch?v=B0QDRqHNNE8> Published December 2, 2019. Accessed July 28, 2020.
10. Sheena Sharma. Guided Meditation for Chronic Pain & Fibromyalgia Pain Relief, Relaxation, Sleep Aid, Anxiety [Video]. YouTube. <https://www.youtube.com/watch?v=vhTcG2POu20> Published March 12, 2020. Accessed July 28, 2020.
