# Supplementary material for: Implementing an Interactive Introduction to Complementary Medicine for Chronic Pain Management Into the Medical School Curriculum
Source: MedEdPORTAL. 2020 Dec 29;16:11056. doi: 10.15766/mep_2374-8265.11056 (PMC7780745; doi:10.15766/mep_2374-8265.11056)
Supplement: Supplementary file 1 — CAM Lecture.pptxStudent Perspective Script.docxFacilitator Guide.docxPresession Survey.docxPostSession Survey.docx [file mep_2374-8265.11056-s001.zip › D. Presession Survey.docx]

HESJ Complementary Medicine Pre-Survey

Start of Block: Default Question Block

Research from the NIH shows that 38% of Americans use at least one form of complementary and alternative medicine. The most common complementary therapies used are meditation and yoga, and Americans are most likely to use these types of practices to help treat chronic musculoskeletal pain. In light of the current opioid epidemic, learning about the evidence base behind these therapies for the management of chronic pain takes on even greater importance. As part of a broader curriculum on the opioid epidemic, an interactive HESJ session will be held on January 10th. These therapies may also provide important modalities for self-care, an essential part of physician wellness. In preparation for the session, please fill out the following questionnaire.

Q1 Enter your unique identifier: Last 2 letters of your undergraduate university name (e.g. RS for Rutgers University), last 2 numbers of your cell phone number, and first 2 letters of your birthplace town in the format XY00AB.

________________________________________________________________

Q2 Rate your interest level in learning more about common complementary medicine practices for managing chronic pain (yoga, meditation, tai chi, acupuncture, hypnosis, cupping etc.)?

|  | No Interest | Very Interested |
| --- | --- | --- |

|  | 1 | 2 | 3 | 4 | 5 |
| --- | --- | --- | --- | --- | --- |

| Level of Interest | 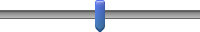 |
| --- | --- |

Q3 How familiar are you with common complementary medicine practices?

|  | Not Familiar | Somewhat Familiar | Familiar | Familiar and Somewhat Practice | Familiar and Routinely Practice |
| --- | --- | --- | --- | --- | --- |

|  | 1 | 2 | 3 | 4 | 5 |
| --- | --- | --- | --- | --- | --- |

| Yoga | 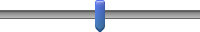 |
| --- | --- |
| Tai Chi | 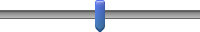 |
| Acupuncture | 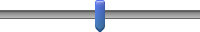 |
| Meditation/Mindfulness | 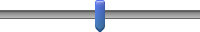 |
| Hypnosis | 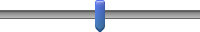 |
| Other | 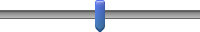 |

Q4 Rate your knowledge about the evidence base behind common complementary medicine practices for treating chronic pain?

|  | Minimal | Average | Superior |
| --- | --- | --- | --- |

|  | 1 | 2 | 3 | 4 | 5 |
| --- | --- | --- | --- | --- | --- |

| Yoga | 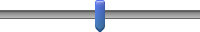 |
| --- | --- |
| Tai Chi | 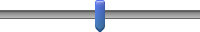 |
| Acupuncture | 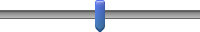 |
| Meditation/Mindfulness | 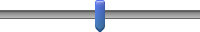 |
| Hypnosis | 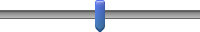 |
| Other | 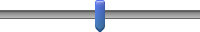 |

Q5 How comfortable do you feel counseling your patients about these practices for treating chronic pain?

|  | Not Comfortable at All | Very Comfortable |
| --- | --- | --- |

|  | 1 | 2 | 3 | 4 | 5 |
| --- | --- | --- | --- | --- | --- |

| Yoga | 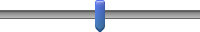 |
| --- | --- |
| Tai Chi | 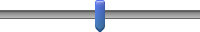 |
| Acupuncture | 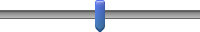 |
| Meditation/Mindfulness | 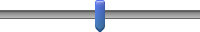 |
| Hypnosis | 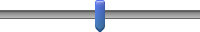 |
| Other | 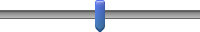 |

Q6 On a scale of 1-5, how important do you think it is for physicians to be familiar with complementary medicine practices?

|  | 1 | 2 | 3 | 4 | 5 |
| --- | --- | --- | --- | --- | --- |

| Importance | 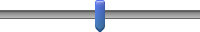 |
| --- | --- |

Q7 How often have you witnessed a bias against patients who practice a complementary medicine discipline by a member of the health care team in the past year?

- Never
- Once or twice
- 3-5 times
- More than 5 times

Q8 Rate the degree to which you agree with the following statement: I am aware of having a personal bias against patients who practice a complementary medicine discipline.

- Not at all
- To a small degree
- To a moderate degree
- To a great degree
- To a considerable degree

Q9 How familiar are you with the financial costs associated with these practices?

|  | Not familiar at all | Very familiar |
| --- | --- | --- |

|  | 1 | 2 | 3 | 4 | 5 |
| --- | --- | --- | --- | --- | --- |

| Yoga | 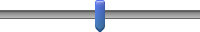 |
| --- | --- |
| Tai Chi | 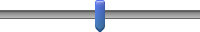 |
| Acupuncture | 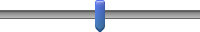 |
| Meditation | 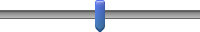 |
| Hypnosis | 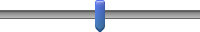 |
| Other | 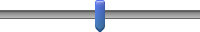 |

Q10 Rate the degree to which you feel able to accomplish the following objectives based on your baseline knowledge.

|  | Hardly at all | To a small degree | To a moderate degree | To a great degree | To a considerable degree |
| --- | --- | --- | --- | --- | --- |
| Describe the methods and evidence base for common complementary approaches to chronic pain management. |  |  |  |  |  |
| Explain the importance of open communication between patients and physicians in discussing complementary therapies. |  |  |  |  |  |
| Examine how social inequity impacts patient access to pain management resources and complementary care. |  |  |  |  |  |
| Engage with a commonly employed complementary medicine modality for chronic pain management. |  |  |  |  |  |

Q11 List 2 barriers you anticipate a patient may face in seeking complementary medicine practices for managing chronic pain.

________________________________________________________________

Q12 What additional topics would you like to learn about with respect to complementary and integrative medicine approaches to chronic pain management?

________________________________________________________________

________________________________________________________________

________________________________________________________________

________________________________________________________________

________________________________________________________________

End of Block: Default Question Block
