# Supplementary material for: Implementing an Interactive Introduction to Complementary Medicine for Chronic Pain Management Into the Medical School Curriculum
Source: MedEdPORTAL. 2020 Dec 29;16:11056. doi: 10.15766/mep_2374-8265.11056 (PMC7780745; doi:10.15766/mep_2374-8265.11056)
Supplement: Supplementary file 1 — CAM Lecture.pptxStudent Perspective Script.docxFacilitator Guide.docxPresession Survey.docxPostSession Survey.docx [file mep_2374-8265.11056-s001.zip › E. Postsession Survey.docx]

HESJ Complementary Medicine Post-Survey

Start of Block: Default Question Block

Q1 Enter your unique identifier: Last 2 letters of your undergraduate university name (e.g. RS for Rutgers University), last 2 numbers of your cell phone number, and first 2 letters of your birthplace town in the format XY00AB.

________________________________________________________________

Q2 In which experiential activity did you participate?

- Yoga
- Tai Chi
- Meditation

Q3 Rate your knowledge about the evidence base behind common complementary medicine practices for treating chronic pain?

|  | Minimal | Average | Superior |
| --- | --- | --- | --- |

|  | 1 | 2 | 3 | 4 | 5 |
| --- | --- | --- | --- | --- | --- |

| Yoga | 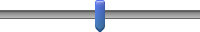 |
| --- | --- |
| Tai Chi | 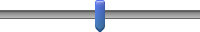 |
| Acupuncture | 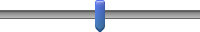 |
| Meditation/Mindfulness | 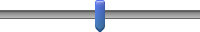 |
| Hypnosis | 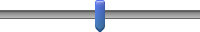 |

Q4 How comfortable do you feel counseling your patients about these practices for treating chronic pain?

|  | Not comfortable at all | Very Comfortable |
| --- | --- | --- |

|  | 1 | 2 | 3 | 4 | 5 |
| --- | --- | --- | --- | --- | --- |

| Yoga | 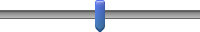 |
| --- | --- |
| Tai Chi | 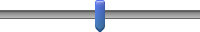 |
| Acupuncture | 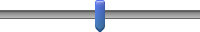 |
| Meditation/Mindfulness | 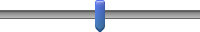 |
| Hypnosis | 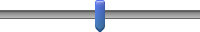 |

Q5 On a scale of 1-5, how important do you think it is for physicians to be familiar with complementary medicine practices?

|  | 1 | 2 | 3 | 4 | 5 |
| --- | --- | --- | --- | --- | --- |

| Importance | 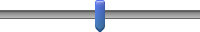 |
| --- | --- |

Q6 How familiar are you with the financial costs associated with these practices?

|  | Not familiar at all | Very familiar |
| --- | --- | --- |

|  | 1 | 2 | 3 | 4 | 5 |
| --- | --- | --- | --- | --- | --- |

| Yoga | 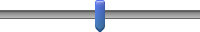 |
| --- | --- |
| Tai Chi | 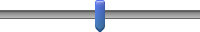 |
| Acupuncture | 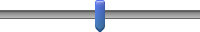 |
| Meditation/Mindfulness | 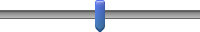 |
| Hypnosis | 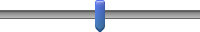 |

Q7 List 2 barriers patients may face in seeking complementary medicine practices for managing chronic pain.

________________________________________________________________

Q8 Rate your ability to accomplish the following after completing this educational session:

|  | Hardly at all | To a small degree | To a moderate degree | To a great degree | To a considerable degree |
| --- | --- | --- | --- | --- | --- |
| Describe the methods and evidence base for common complementary approaches to chronic pain management. |  |  |  |  |  |
| Explain the importance of open communication between patients and physicians in discussing complementary therapies. |  |  |  |  |  |
| Examine how social inequity impacts patient access to pain management resources and complementary care. |  |  |  |  |  |
| Engage with a commonly employed complementary medicine modality for chronic pain management. |  |  |  |  |  |

Q9 Rate the degree to which you agree with the following statement: Additional training on this topic will be beneficial to my training to become a doctor.

- Hardly at all
- To a small degree
- To a moderate degree
- To a great degree
- To a considerable degree

Q10 Please comment on the strengths of this session.

________________________________________________________________

________________________________________________________________

________________________________________________________________

________________________________________________________________

________________________________________________________________

Q11 Please include your suggestions for how the session may be improved.

________________________________________________________________

________________________________________________________________

________________________________________________________________

________________________________________________________________

________________________________________________________________

End of Block: Default Question Block
